# Quantifying Molecular Properties of Hexagonal Water Clusters

**DOI:** 10.1002/open.202500149

**Published:** 2025-06-01

**Authors:** Giuseppe Lanza

**Affiliations:** ^1^ Dipartimento di Scienze del Farmaco e della Salute Università di Catania Viale A. Doria 6 95125 Catania Italy

**Keywords:** DFT calculations, hexagonal clusters, ice, stability, vibrational spectra

## Abstract

Density functional theory and polarizable continuum model (DFT/M06‐2X/PCM) calculations, which make use of Gaussian basis sets, have been carried out to study the molecular properties of large hexagonal water clusters as a model for ice Ih. The series of (H_2_O)_*n*_ (*n* = 96–332) clusters has been designed to reduce dangling hydrogen bonds on the surface and to preserve hexagonal crystallinity as much as possible. Large cluster structures are very stable, and computed electronic energy and entropy show asymptotic behavior with data astonishingly close to experimental thermodynamics. An excellent computation/experimental comparison has also been obtained for the neutron and X‐ray diffraction structural data, as well as for infrared, Raman, inelastic neutron, and hyper‐Raman fundamental vibrational frequencies. The overall results suggest that the use of large‐size clusters, together with reliable and standard quantum chemical methods, is a highly promising and safe methodology to investigate experimentally challenging phenomena in water science.

## Graphical abstract

Standard quantum chemical calculations on large hexagonal water clusters allow us to analyze the fundamental properties of ice‐Ih. Computed geometrical structure, energies, and vibrational spectra are astonishingly close to experimental data without the introduction of any external empirical parameters. The proposed methodology is a highly promising and safe approach to investigate experimentally challenging phenomena in water science.

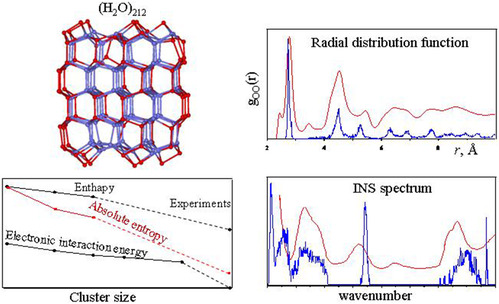

## 1 Introduction

Water clusters have attracted increasing attention from both experimental and computational chemists in recent years.^[^ ^1^, ^2^, ^3^, ^4^, ^5^, ^6^, ^7^, ^8^, ^9^, ^10^, ^11^, ^12^ ^]^ They are considered as a link between gas and bulk phases, and large clusters have properties that vary, smoothly increasing the size and converge to those of the liquid or solid water phases. The transition itself can only be addressed and corroborated by studying molecular properties of clusters of increasing size in order to clarify hydrogen‐bonded (HB) network changes that ultimately lead to different macroscopic behaviors.

From a computational point of view, the cluster approach seems to be a sustainable strategy to examine condensed states of water. The finite size of the systems does not require the use of periodic boundary conditions that can introduce artificial correlations; however, some imperfection could appear at the interface of the cluster with a vacuum. If the size of the cluster is large, the limitations become less critical, and the structure and dynamics of various and interesting behaviors at the microscopic level can be studied. On the other hand, the isolated cluster makes theoretical treatments easier than those required for the condensed states, thus, the plethora of accurate quantum chemical methods available can be applied routinely. The need to use accurate and unbiased methods of electronic structure theory is dictated by the fact that, at the microscopic level, bulky water molecules exhibit unpredictable and unusual behaviors that give rise to the extraordinary macroscopic properties. For example, by changing the temperature, pressure, and preparation procedure in the solid state, there are 19 ice structures known so far, as well as four families of amorphous ice, namely low‐, medium‐, high‐, and very high‐density, with different local organization and interactions between molecules.^[^ ^5^, ^13^ ^]^

In water science, the solid state is of particular importance given that, the low mobility, limited to vibrational motions, allows for the attainment of experimental pieces of information that can be a guideline in the development of suitable empirical models^[^ ^14^, ^15^, ^16^ ^]^ and can also serve as a reference point for testing accuracy and reliability of ab initio quantum chemical methodologies free of external parametrization.^[^ ^17^ ^]^ Among the different allotropic states, the hexagonal ice, Ih, is of paramount importance, and the description of the physical processes occurring in this solid region is particularly important in the physical–chemical field as a starting point for the study of the properties of various water phases. There have been many researches that make use of empirical‐derived potentials^[^ ^18^, ^19^, ^20^, ^21^, ^22^ ^]^ and also some ad hoc quantum chemical^[^ ^23^, ^24^, ^25^, ^26^, ^27^, ^28^, ^29^ ^]^ studies from which to derive specific structural, thermodynamic, and spectroscopic information. However, the cluster approach combined with high‐level quantum chemical computations, which make use of Gaussian basis functions, allows for a new all‐encompassing setup covering many topics of ice Ih.

In the present study, using a bottom‐up approach,^[^ ^30^, ^31^, ^32^, ^33^, ^34^ ^]^ a large series of hexagonal ice‐like clusters (H_2_O)_*n*_ (*n* = 96, 102, 160, 172, 212, 254, and 332) have been studied to examine a vast range of fundamental molecular properties of ice Ih, some of which have been and are still a matter of debate.^[^ ^23^, ^24^, ^25^, ^26^, ^27^, ^28^, ^29^ ^]^ Among them, we mentioned the ice Ih formation electronic energy, enthalpy, and entropy at 273 K, neutron and X‐ray diffraction results, as well as infrared (IR), Raman, inelastic neutron, and hyper‐Raman spectra. Regarding the vibrational spectra, there have been several computations, also using quantum chemical methodologies,^[^ ^25^, ^26^, ^27^, ^28^, ^29^ ^]^ while for the thermodynamic property computations, there is a paucity of information in using quantum chemical methodologies.^[^ ^19^, ^20^, ^21^, ^22^, ^23^, ^24^ ^]^

The novelties of the present approach are its reliability‐generality, simplicity, expandability, and reproducibility, which have not normally been present altogether in previous studies: 1) Full quantum chemical computations guarantee predictive accuracy in all regions of the water phase diagram, in solutions and reactive events. This feature allows us to compute many properties with the same reliability. Some of them (interaction energy and zero‐point energy (ZPE)) have only been studied using classical potentials or plane‐wave‐based quantum methods. Other properties, as the absolute entropy, have never been studied; 2) The large size of clusters and the chemical structure intuition for the minimal basal face reconstruction preserve Ih ice crystallinity and allow for a realistic description of molecular properties; 3) Although the construction has been halted at the 332 cluster size, the procedure can be extended easily to clusters of any dimension. Even though the reported structures are not the global minima for large clusters (low value of the surface‐area‐to‐volume ratio), they become very stable; 4) As far as vibrational spectra interpretation, a 192‐mer cluster has been used by Bowman et al.^[^ ^29^ ^]^ However, the surface is disordered (87 molecules) and only a portion (105 molecules) preserves crystallinity; thus, data interpretation/comparison requires a screening of normal modes. Several studies derive the vibrational frequencies using periodic 64 (or less) molecules as a unit cell of ice Ih.^[^ ^25^, ^26^, ^27^, ^28^ ^]^ The small size of the unit cell could introduce some artificial correlations in proton positions, and for example, there are difficulties in reproducing the sharp acoustic peak (60 cm^−1^) in the experimental spectra. The absence of periodic conditions and the large proton random orientation, which instead is possible to reach in present isolated large clusters, could better represent the ice Ih disordered proton configuration.

Overall, the present study indicates an astonishingly computation/experimental comparison that is particularly interesting because the methodology uses standard quantum chemical procedures available to all researchers, hence fully reproducible, and further expandable to many other specific issues in the water science.

## 2 Calculation Methods

The geometries were optimized using the DFT/M06‐2X electronic structure method,^[^ ^35^ ^]^ employing the 6‐31+G* basis set and including implicitly solvent effects.^[^ ^36^ ^]^ Minima were characterized by evaluating the Hessian matrix and the associated harmonic vibrational frequencies. Implicit solvent effects were modeled using the polarized continuum method (PCM), adopting a 78.36 dielectric constant for water as implemented in the Gaussian16 program.^[^ ^37^ ^]^

To improve energetics and to reduce intermolecular basis set superposition error, single‐point energy evaluation at the optimized geometries was performed using the more accurate aug‐cc‐pVDZ basis set. The electronic energies were corrected for zero‐point vibrational and thermal energies to obtain enthalpy and entropy at 273 K. To calculate the entropy, *S*°_273_, the different contributions to the partition function were evaluated by using the standard expressions for an ideal gas in the canonical ensemble, the particle in a 3D box, the harmonic oscillator, and the rigid rotor approximations.

The Minnesota functionals M05‐Class and M06‐Class include the medium‐range dispersion effects,^[^ ^36^ ^]^ and the reliability of present M06‐2X‐based computations has been previously evaluated using extensive traditional ab initio MP2 (and in some cases MP4‐SDQ) calculations with the 6‐31+G* basis set for optimization/Hessian followed by the single‐point aug‐cc‐pVTZ energy.^[^ ^30^, ^31^, ^32^ ^]^

The IR absorption intensities have been obtained from the molecular dipole moment derivatives with respect to the nuclear Cartesian coordinates. Raman intensities have been obtained from dipole derivatives with respect to the electric field and nuclear Cartesian coordinates. The IR and Raman spectra have been generated with the GaussView 5.0 software.^[^ ^38^ ^]^ The inelastic neutron scattering (INS) spectrum has been simulated through the convolution of Gaussians of the density of states (the histogram) with a FWHM of 40 cm^−1^.^[^ ^39^ ^]^

Stiff intramolecular modes are characterized by a deep potential well, thus, the harmonic oscillator is a reliable approximation. Anharmonic corrections are modest, and simple scaling factors are used to evaluate vibrational frequencies without computational cost. For low‐frequency modes, the shallow potential well presents various minima, the scaling factors are doubtful, and rigorous computational treatments are necessary.^[^ ^40^, ^41^ ^]^ However, they are affordable only for systems with less than 20 atoms.^[^ ^40^ ^]^ Presently, vibrational spectra interpretation is based on harmonic frequencies without any scaling factor.

## 3 Results and Discussion

### 3.1 Structure

The global minimum searches for water clusters show that the most stable structures of small‐sized clusters are amorphous and dominated by four‐ and five‐membered rings.^[^ ^6^, ^7^, ^8^, ^9^, ^10^, ^11^, ^12^ ^]^ The first formation of a fully‐hydrated six‐membered ring occurs for the (H_2_O)_52_ cluster.^[^ ^6^ ^]^ Low energy structures (not global minima) reported by Buch et al. show a more extended six‐membered partially ordered core for the (H_2_O)_123_ cluster but the appearance of a (strained) crystal core with six‐membered rings occurs for the (H_2_O)_293_.^[^ ^10^ ^]^ For larger clusters (*n* = 600 and 931) a well‐crystallized interior core with more than 80% of the molecules in the Ih arrangement are formed together with a somewhat disordered surface.^[^ ^11^ ^]^ The growth of crystallinity, as the cluster size increases, makes it easier to find the global minimum or some structures close to it. Both experimental and computational studies suggested a nearly spherical shape with an amorphous surface followed by a partially ordered subsurface and then the crystalline Ih core.^[^ ^9^ ^]^ These configurations decrease the surface‐area/energy reducing the dangling HBs and oxygen lone pairs at the expense of crystallinity. Nevertheless, the aims of the present study are the ice Ih molecular properties; the best comparison to bulk experimental data could be reached considering clusters as large as possible with an Ih‐core with some “customized” surface reconstruction to reduce dangling HBs. To reduce heavy surface reconstruction, we avoided considering spherical shapes and tedious global minima searches; thus, present clusters appear as cuboids, i.e., ice nanocrystals cut along the basal and prismatic faces. In this simple approach, the minimal area criterion in tiling surface reconstruction is not satisfied, and energetically more stable structures are present for small and medium clusters.^[^ ^32^ ^]^ For large clusters (*n* > 250), surface amorphization is less important with respect to the Ih core. Therefore, there will be structures slightly more stable than those presently considered, but we cannot give a reasonable estimation. The reliability of the minimal surface reconstruction becomes evident when looking at the asymptotic behavior of the electronic interaction energy (vide infra).

The present nanocrystal building offers three advantages: i) a wide selection of clusters with low energy structures can be derived without tedious global minimum searches; ii) a reliable reference for many molecular properties such as bond distance, energy, vibrational frequencies, and so on; iii) allowing for a vis‐à‐vis comparison between computational results and the plethora of experimental results on ice Ih.

**Figure 1:**
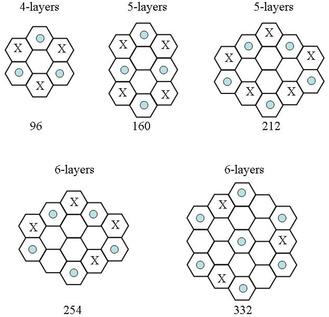
Schematic structures of hexagonal clusters (view from the c‐axis). The blue circles indicate molecules capping surface chairs, while X indicates the formation of pentagons on basal surfaces.

**Figure 2:**
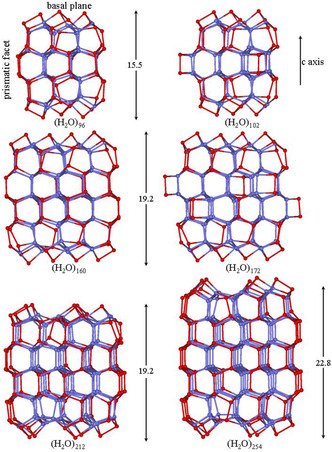
M06‐2X/6‐31+G*/PCM optimized structures of hexagonal, (H_2_O)_*n*_ *n* = 96–254, clusters viewed from a prismatic face. Hydrogen atoms have been omitted for clarity, and the sticks represent the O—H bonds involved in hydrogen bonding. The surface tricoordinate molecules are in red, while tetracoordinate molecules are in blue.

The simplest (H_2_O)_96_ cluster consists of four layers of 24 water molecules hexagonally arranged along the “c‐axis” (**Figure** **1**), while hydrogen atoms are randomly oriented within the constraint of network completeness. Few preliminary geometry optimizations, with a smaller basis set, 6‐31G*, for different proton configurations on the constructed surface, have been performed to choose the most favorable structure. The (H_2_O)_96_ cluster presents 12 bicoordinate water molecules that are highly energetically unfavorable, thus, three of them on each hexagonal faces have been removed to form three pentagons and have been positioned above the adjacent hexamer chairs to saturate their dangling hydrogen bonds. The basal surfaces of the cluster terminate by pentagons and capped hexagons (**Figure** **2**). The prismatic surfaces present only tri‐ and tetracoordinated molecules and no alterations are strictly necessary to generate energetically favorable geometries. The hydrogen atoms are randomly oriented and the tricoordinated molecules are equally distributed among double acceptor (AAD) and double donor (DDA) configurations. The (H_2_O)_160_ cluster was built stacking five layers of 32 chair‐form water hexamer molecules (Figure 1). The 16 bicoordinate molecules were repositioned to form four pentagons and four capped hexagons on both hexagonal surfaces (Figure 1 and 2). The average coordination number of water molecules increases on expanding the cluster size and the electronic interaction energy per molecule becomes more negative (**Table** **1**).

**Table 1:** Average coordination number (c.n.) of water, interaction electronic energy, ZPE variation, enthalpy (kcal mol^−1^), and entropy (cal mol^−1^ K^−1^) per water molecule in water deposition.

	c.n.	E_int_	ΔZPE	D_0_	ΔH°_273_	S°_273_
(H_2_O)_96_	3.50	–12.63	2.71	–9.92	–10.86	10.04
(H_2_O)_102_	3.59	–12.63				
(H_2_O)_160_	3.56	–12.85	2.75	–10.10	–11.04	9.60
(H_2_O)_172_	3.66	–12.90				
(H_2_O)_212_	3.59	–12.99	2.76	–10.23	–11.18	9.43
(H_2_O)_254_	3.60	–13.05				
(H_2_O)_332_	3.63	–13.20		*–*10.44		
Ice‐experiments	4.00	–14.07a)	2.75a)	–11.31	–12.2b)	8.3c)

a) See ref. [43].

b) See ref. [44].

c) See ref. [45].

Some test calculations have been carried out to reduce the number of tricoordinate molecules on prismatic faces of the (H_2_O)_96_ and (H_2_O)_160_ clusters, thus, three and six water dimers (HO—H···OH_2_) have been added to hexamer boats, respectively, to form the (H_2_O)_102_ and (H_2_O)_172_ clusters (Figure 2). In spite of the average coordination number of water molecules increasing, there is no energy gain for the (H_2_O)_102_ cluster and a modest gain for the (H_2_O)_172_ cluster (Table 1). Even though the quantity of HB count increases, the strong distortions from tetrahedral bonding in four‐membered rings do not result in a significant energetic improvement. Considering these results, we overlooked the quantity reduction of tricoordinate molecules on prismatic faces in the cluster size expansion.

The (H_2_O)_212_ and (H_2_O)_254_ clusters consist of five and six stacks of 42 molecule chair‐form hexamers. To reduce undercoordination on the basal facets, half of the bicoordinate molecules have been repositioned to form pentagons and capped chairs (Figure 1 and 2). For both clusters, two additional molecules have been added to saturate three dangling HBs on two chair‐hexamer. The coordination number slightly increases and the clustering becomes progressively more exoenergetic (Table 1).

**Figure 3:**
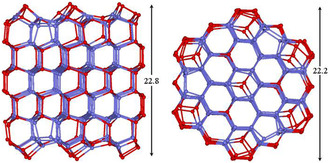
M06‐2X/6‐31+G*/PCM optimized structures of hexagonal (H_2_O)_332_ clusters view from a prismatic (left) and the hexagonal (right) faces. Hydrogen atoms have been omitted for clarity, and the sticks represent the O—H bonds involved in hydrogen bonding. The surface tricoordinate molecules are in red, while tetracoordinate molecules are in blue.

The (H_2_O)_332_ consists of six stacks of 54 molecule chair‐form hexamers with the bicoordinate molecules on basal facets rearranged in the usual way to form pentagons and capped hexagons (**Figure** 1 and **3**). Furthermore, eight molecules have been added to saturate a large part of the remaining dangling HBs of the basal facets. As expected, both the average coordination number and interaction energy per molecule increased (Table 1). Overall, the cluster shows 122 tricoordinate molecules (61 AAD and 61 DDA) and 210 tetracoordinate molecules.

### 3.2 Energies

**Figure 4:**
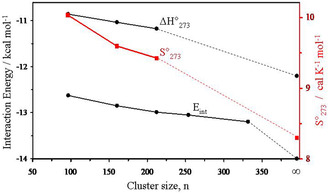
M06‐2X/aug‐cc‐pVDZ/PCM interaction electronic energy and enthalpy per water molecule (*E*_int_ and Δ*H*°_273_, black curve, scale on the left). M06‐2X/6‐31+G*/PCM absolute entropy per water molecule (*S*°_273_ red curve, scale on the right).

The electronic interaction energy curve (**Figure** **4**) shows that a large part of the asymptotic behavior is captured, however, the coordination number for the (H_2_O)_332_ cluster is far from bulky ice (3.63 vs 4) and some further gain in energy (roughly of −0.6 kcal mol^−1^) could be anticipated in cluster enlargement. For instance, in the TIP4P global optimization of the (H_2_O)_293_, (H_2_O)_600_, and (H_2_O)_931_ clusters, Buch et al.^[^ ^11^ ^]^ reported an exoenergeticity increase of −0.3 and −0.1 kcal mol^−1^ on size enlargement, respectively.

Accurate thermal measurements on the gas‐phase water molecules assembling in the hypothetical Ih ice at 0 K release −11.3 kcal mol^−1^ of heat.^[^ ^42^, ^43^ ^]^ Considering the change in the vibrational state of the molecules, i.e., subtracting the ZPE variation, the electronic energy has been estimated by Whalley,^[^ ^43^ ^]^ −14.07 kcal mol^−1^ (Table 1). The computed electronic energy for the (H_2_O)_332_ cluster, −13.20 kcal mol^−1^, is close enough to the experimental data, and the small difference could be related both to the limited size of the cluster and to the presently adopted electronic structure method. Using molecular dynamics simulations and the TTM and TTM2‐R water models, Xantheas et al. obtained interaction energies at the 0 K limit, more exoenergetic, −15.56 and −14.69 kcal mol^−1^, respectively.^[^ ^19^, ^20^, ^21^ ^]^ The TIP4P value (−13.18 kcal mol^−1^) is comparable to the present value, emphasizing the critical role of the model potential in classical molecular dynamics. Very good computational/experimental comparisons have been obtained using quantum chemical methodology with a plane‐wave basis set.^[^ ^22^, ^23^ ^]^ Thus, the absolute lattice energies (without the ZPE effects) of ice Ih are −13.95 kcal mol^−1^, using the diffusion quantum Monte Carlo methods, and −14.04 and −13.86 kcal mol^−1^, using the embedded many‐body expansion of MP2 and CCSD(T) wavefunction‐based methods, respectively.

The presently computed overall ZPE is 16.2 kcal mol^−1^ per molecule and it is higher than that reported for the ice (15.27 kcal mol^−1^) using molecular dynamics simulations.^[^ ^19^, ^20^, ^21^ ^]^ The difference is due to the overestimation of the stiff frequencies in the M06‐2X/6‐31+G*/PCM theory level (vide infra), however, the effect is present in both gas phase and ice molecules, therefore, the ZPE variations on cluster formation (2.76 kcal mol^−1^) are identical to those estimated experimental values for water deposition (Table 1). Singling out the computed ΔZPE in its intra‐ and intermolecular contributions (−1.00 and 3.76 kcal mol^−1^, respectively), they have opposite signs and the intramolecular variation mainly comes from the perturbations induced to the stretching modes (ν_1_ and ν_3_) while the contribution of the bending (ν_2_) is rather small. Present data are almost identical to those obtained for amorphous clusters, (H_2_O)_55_ and (H_2_O)_123_, at the same level of theory^[^ ^36^ ^]^ and they are in excellent agreement with those estimated by Whalley (−1.19 and 3.94 kcal mol^−1^)^[^ ^43^ ^]^ and by Xantheas et al.^[^ ^19^, ^20^, ^21^ ^]^ for only the intermolecular contribution (3.9 kcal mol^−1^).

Assuming the same ΔZPE for the (H_2_O)_332_ cluster, the calculated deposition enthalpy at 0 K is −10.45 kcal mol^−1^ close enough to experiment (−11.3 kcal mol^−1^).^[^ ^44^ ^]^ On increasing the temperature to 273 K, both computations and experiments indicate an increment of exothermicity (−0.9 kcal mol^−1^, Table 1); thus, the simpler statistical thermodynamic approach adopted here reproduces the features introduced by the thermal motion in vibrations.

The absolute standard entropy at 273.15 K per molecule, S°_273_, of the analyzed hexagonal clusters shows a progressive reduction increasing the size (Table 1 and Figure 4). As the number of molecules forming the cluster increases, the efficiency of the packing improves, the average coordination number of water molecules increases, and the system tends toward a more ordered state with a lower absolute entropy value. In spite of the simplicity of the statistical thermodynamic treatment and limitations of the cluster approach, the experiment and computation are numerically comparable, 8.3 versus 9.43 cal K^−1^ mol^−1^.^[^ ^45^ ^]^

### 3.3 Geometrical Parameters

The covalent O—H bond length in ice Ih has been a matter of debate for a long time; however, nowadays, the 0.985 ± 0.006 Å value is widely accepted (see discussion on reference^[^ ^28^ ^]^). The computed O—H bond length distribution for the (H_2_O)_332_ cluster shows a narrow distribution centered at 0.990 Å, very close to the experimental value (Figure S1, Supporting Information). The peak at 0.970 Å in Figure S1 (Supporting Information) is due to the 61 O—H bonds not involved in hydrogen bonding on the surface. The H—O—H bond angle distribution shows a curve centered at 106.5°, which spreads in the 105°–108° range (Figure S1, Supporting Information) in full agreement with the value experimentally assessed (106.6 ± 1.5° and 107 ± 1°, see reference^[^ ^28^ ^]^). Few angles are out of this interval, and they are due to some strained molecules on the basal surfaces. The metrical parameters for a water isolated molecule (0.966 Å and 105.8°) are close to the “equilibrium” geometry experimentally derived (0.9584 Å and 104.45°).^[^ ^46^ ^]^ Present computations correctly predict the O—H bond lengthening and the widening of the H—O—H bond angle, once the isolated water molecules are embedded in the ice lattice.^[^ ^47^ ^]^ For the H—O—H bond angle enlargement, there has been some discussion in the literature because many empirical potentials are not able to reproduce this trend.^[^ ^47^ ^]^

The computed HB O···O and OH···O contacts spread in the 2.65–2.90 and 1.70–1.90 Å ranges, with the maximum values (2.73 and 1.75 Å, Figure S1, Supporting Information) close to the experimental ones (2.75 and 1.75 Å) quoted in ref. [48]. The low temperature at which the experiments were carried out implies small thermal effects on metrical parameters, thus, the thermal average bond length and bond angle from neutron, X‐ray, and electron diffraction studies are close enough to the equilibrium values, and the computation/experiment comparison is excellent.

The statistical distribution of the computed O···O···O angles of HB molecules falls mainly in the 100°–120° range, with the maximum at 110° very close to tetrahedrality (Figure S1, Supporting Information). The several peaks that are out of this range are due to the capping molecules of the hexagonal surfaces that form squares and strained pentagons. Along the same line, the O—H···O angles of HB atoms show only small deviations (a few degrees) from linearity, except for the strained four‐ and five‐membered rings (Figure S1, Supporting Information).

**Figure 5:**
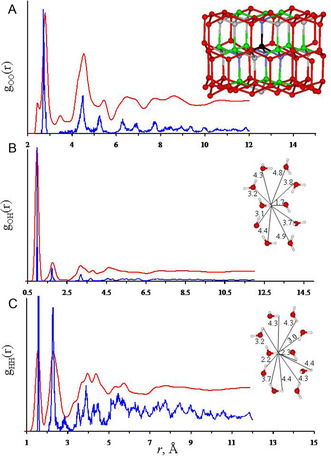
Experimental A) O—O, B) O—H, and C) H—H PDFs for ice Ih at 220 K (red)^[^ ^50^ ^]^ and computed distributions for the optimized geometry of the (H_2_O)_332_ cluster. The computed data refer to the static motion‐less minimum at 0 K. The inset in A shows a portion of the (H_2_O)_332_ cluster, which are the first (blue), the second (green), and the third (gray) neighbors of the oxygen reference atom (black). Inserts in B and C show some computed distance in the hexagonal‐chair.

In the ice Ih structure, each oxygen atom has four HB molecules (the first‐neighbor O···O distance is 2.72 Å, insert in **Figure** **5**A) and 12 s‐neighbors (≈4.5 Å). In the basal plane, each oxygen atom is involved in three chairs and the contact with distal oxygen is ≈5.3 Å (the third‐neighbors). Along the prismatic planes, each oxygen atom is involved in nine boat hexamers. Six of these boats (those that contain the HB along the c‐axis) give rise to the contact with distal oxygen of ≈5.3 Å (the third‐neighbors), while the remaining three boats have a common distal oxygen at 4.6 Å. The latter O···O contact is closer to the second‐neighbors than the third‐ones, even though the oxygen occupies a distal position in the hexagonal boat. Thus, the ratio of oxygen numbers between first and second peaks in the pair distribution function (PDF) is expected to be 4:13. Similar arguments for successive coordination shells allow us to find that the expected area ratio for the ideal ice Ih is of 4:(12 + 1):9:12:(9 + 2):18.^[^ ^5^ ^]^

Two sets of neutron diffraction of powder ice Ih in the form of OO, OH, and HH partial structure factors at 220 and 258 K have been reported by Soper.^[^ ^49^, ^50^, ^51^ ^]^ The latter measurements, using H_2_O, D_2_O, and two mixtures of these molecules, show an enlargement of bands and less detail with respect to the former, probably due to the increased thermal motion. Experimental PDFs are compared with those derived from the (H_2_O)_332_ cluster in Figure 5. There is a satisfactory agreement between experimental and calculated data. Thus, the main features of the experimental g_OO_(r) at 2.78, 4.53, 5.46, 5.8–7.2 envelope, 7.77, 8.61, and the flat peak at 10.77 Å found counterparts in the O···O distance distribution in the cluster (Figure 5A).

The first peak is presently computed at a shorter distance of 2.72 Å. This region has been also studied through extended X‐ray absorption fine structure spectroscopy at 256.4 K, which is very sensitive in this region, and the probable distance (peak position) 2.71 Å is in better agreement with present data.^[^ ^52^ ^]^ The second and the third peaks, computed at 4.5 and 5.3 Å, correspond to the remaining oxygen in the same hexagon. The peaks at 6.4, 6.9, and 7.7 Å correlate with oxygen of adjacent hexagons, i.e., the first hexagon coordination. The computed peak area normalized to the average number of formed HBs for the first three peaks (3.63:9.6:5.9) are compatible to those expected for the ideal ice Ih structure (4:13:9), while for the successive coordination shell, the comparison gets worse because of the reduced cluster size.

The experimental g_OH_(r) function shows five peaks well‐defined at 0.96 (covalent O—H bond), 1.77 (HB), 3.18, 3.78, and 4.62 Å (oxygen of the hexagon). All features match very well with the computed O···H PDF (0.99, 1.75, 3.2, 3.7, and 4.6 Å) for the (H_2_O)_332_ cluster (Figure 5B). The first peak in the experimental g_HH_(r) corresponds to the intramolecular H···H contact, and it is close to the computed one (1.53 vs. 1.59 Å, respectively, Figure 5C). The second peak is due to the directly HB molecules, O—H···OH_2_ (2.28 and 2.29 Å, respectively). After that, the experimental g_HH_(r) shows two maxima at 3.96 and 4.38 Å, with shoulders at lower and higher distances. These maxima correlate with hydrogen atoms belonging to molecules of the hexagons, and they are close to the values computationally derived 3.92, and 4.48 Å.

### 3.4 Vibrational Spectra of Ice Ih

**Figure 6:**
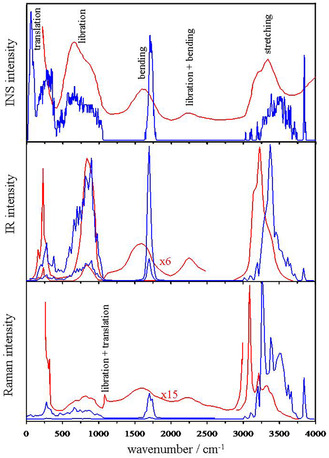
Computed vibrational spectra for the hexagonal (H_2_O)_212_ cluster (blue) and experimental INS, IR and Raman spectra for ice Ih (red). The measured spectra have been digitized from refs. [55] (Figure 3), [56] (Figure 1) and [58] (Figure 1 and 2).

The vibrational frequency changes provide the main manifestation of the tridimensional HB network in different states of water. The spectra of ice present two groups of bands due to high‐energy intramolecular vibrations (>1600 cm^−1^, **Figure** **6**) and low‐energy intermolecular vibrations (<1050 cm^−1^). The intensity of the INS spectrum is directly proportional to the phonon density of states,^[^ ^53^, ^54^, ^55^ ^]^ and all vibrations produce signals, while IR, Raman, and hyper‐Raman bands are modulated by the dipole, polarizability, and hyperpolarizabilty, respectively, which are often not easily predictable from simple selection rules due to proton disorder.^[^ ^56^, ^57^, ^58^, ^59^, ^60^, ^61^ ^]^ Unfortunately, the computation of the vibrational spectra of the largest (H_2_O)_332_ cluster is highly demanding. However, the (H_2_O)_212_ cluster is representative enough of the Ih structure, therefore, the present analysis is based on this system.

There are 424 stretching modes of the O—H covalent bonds. In the range 3834‐3863 cm^−1^, there are 43 stretches of the free O—H bonds related to AAD molecules of the surface (see computed density of states in Figure 6). Generally, they are localized on one molecule, but sometimes, they weakly couple in phase or out of phase with other spatially and energetically close modes. The free O—H are the strongest covalent bonds because they are not weakened by hydrogen bonds and the frequency is close to the stiffer symmetric and antisymmetric stretches of isolated water (3812 and 3936 cm^−1^, respectively). Experimentally, free O—H stretching on the cluster surface is observed as a sharp peak at ≈3700 cm^−1^.^[^ ^11^, ^62^ ^]^ At lower frequency (≈3600 cm^−1^), there are two O—H stretches of the DDA molecules, always coupled with the modes of the HB molecules. Experimentally, for small‐sized clusters, the DDA group is observed at ≈3550 cm^−1^, while for large clusters, it is responsible for the broadening of the main IR absorption band in the high‐energy wing.^[^ ^11^, ^62^ ^]^ The weaker O—H stretches of AAD and DDA molecules, almost always coupled with the modes of the HB molecules, are computed in the 3027–3300 cm^−1^ region. They appear in IR spectra of small clusters from 2950 to 3100 cm^−1^^[^ ^62^ ^]^ for medium clusters at 3213 cm^−1^, while for large clusters, they widen the main peak in the low frequency region.^[^ ^11^, ^62^ ^]^

Beyond these details at both ends of the O—H stretching band, the curves obtained for the tetracoordinate molecules display at the center, the band shape of INS, IR, and Raman spectra similar to those experimentally reported (Figure 6 and **Table** **2**). The computed IR spectrum exhibits a peak at 3380 cm^−1^ with shoulders at ≈3290 and ≈3440 cm^−1^ in analogy to the experimental one (3220, ≈3150, and ≈3380 cm^−1^).^[^ ^56^ ^]^ The computed Raman spectrum displays three overlapping bands at 3260, 3380, and 3510 cm^−1^, while experimental bands occur at 3083, 3209, and 3323 cm^−1^.^[^ ^58^ ^]^ The computed density of the states of bond frequencies show a large distribution (about 3300–3700 cm^−1^) centered at ≈3500 cm^−1^, similarly to the experimental features.^[^ ^54^ ^]^ It is interesting to note that the maximum shifts at lower frequencies, ongoing from INS to IR and Raman spectra, as experimentally observed. Because the selection rules of hyper‐Raman scattering and IR absorption derive from the odd orders of the electric dipole moment, the obtained spectra are similar to some extent, different from Raman scattering, which derives from the second‐order effect (see Figure 5 in ref. [61]). Thus, the experimental hyper‐Raman spectrum shows a broad band centered at 3307 cm^−1^ with a shoulder at 3197 cm^−1^ (Table 2). The main difference with experimental spectra concerns the blueshift (≈170 cm^−1^) in computed stretching frequencies due to the M06‐2X/6‐31+G* theory level, and basis set improvement will reduce this discrepancy.^[^ ^63^ ^]^ An eigenvector analysis of these vibrations shows that all monomer oscillations mix strongly and involve large regions of the cluster. This is in agreement with previous molecular dynamic interpretations^[^ ^64^ ^]^ and with 2D IR spectroscopic findings.^[^ ^65^, ^66^ ^]^ However, present computations suggest that high frequency vibrations are dominated by the antisymmetric stretch of the various monomers, while the low frequency region is characterized by symmetric stretches.

**Table 2:** Computed and experimental wavenumbers of ice Ih. Sp = sharp peak; p = peak; bp = broad peak; sh = shoulder; b = bump.

	INS		IR		Raman		Hyper‐Raman
	Calc.	Exper.a)	Calc.	Exper.b)	Calc.	Exper.c)	Exper.d)
Stretching	3500 bp	3307 bp	3440 sh 3380 p 3290 sh	3380 sh 3220 p 3150 sh	3510 p 3380 p 3260 sp	3323 p 3209 p 3083 sp	3307 sh 3197 p
Bending	1715 p	1613 p	1700 p	1590 p	1700 p	1600 p	1646 p
Librations	660 bp 880 sh	658 p 880 sh	900 bp	860 p	500‐1000 bp	800 bp	825 p 515 sp
Translations (optic)	380–180 bp	306 sp 229 sp 153 bp	275 p 240 sh 210 p	229 p 164 p	340 bp 280 p	315 p 225 p	289 p 210 p
Translations (acoustic)	60 p	57 p	60 b	60 p	60 p	58 p	55 b

a) See ref. [54].

b) See ref. [56, 57].

c) See ref. [58, 59].

d) See ref. [61].

The 212 bending modes fall within a rather narrow range, 1640–1781 cm^−1^, and are hardly perturbed by hydrogen bonding (Figure 6). In the low frequency region (1640–1680 cm^−1^), there is the bending of the AAD molecules on the surface, which are freer to oscillate, and the frequency is quite close to the value of the isolated water molecule (1667 cm^−1^). They are localized over a well‐defined region of the cluster. At higher energy, we find the bending of tetracoordinated molecules. These oscillators couple strongly over a large region of the cluster. In agreement with experimental INS, IR, Raman, and hyper‐Raman spectra,^[^ ^54^, ^56^, ^58^, ^61^ ^]^ a single band is predicted but the band center is overestimated by about 100 cm^−1^ due to the medium quality of the basis set adopted in the present computations (Figure 6 and Table 2).

The modes below 1050 cm^−1^ represent librational (hindered rotation) and hindered translational collective motions of molecules (Figure 6). In a simplified view, normal mode analysis shows that only the hydrogen atoms move in librations, while in translations, the entire molecule moves. In agreement with these results, the INS and hyper‐Raman spectra of H_2_O and D_2_O show small variations for the translational bands, given the modest change in the mass of the moving monomer.^[^ ^54^, ^61^ ^]^ While the change in mass has a large effect on the librations, where the vibrations almost exclusively involve hydrogen atoms.^[^ ^54^, ^61^ ^]^

The librations can be classified as: i) out‐of‐plane deformations (twisting and wagging) and ii) in‐plane deformations (rocking). These oscillations involve large regions of the clusters and are largely coupled. At high frequencies, there are twisting motions but as the frequency is reduced (≈980 cm^−1^), the motions show wagging components, and after ≈850 cm^−1^, vibrational modes show rocking components. Librations with frequencies lower than 600 cm^−1^ are localized on the cluster surface. In computed NIS, IR, and Raman spectra, librations appear as a large, broad peak similar to the experimental shapes (Figure 6). In the calculated and experimental INS spectra, the maximum is at about 660 cm^−1^, and a shoulder appears at ≈880 cm^−1^, while the higher resolution spectrum displays well‐separated librational modes.^[^ ^55^ ^]^ Experimental IR and Raman spectra show a less broad band centered at ≈850 cm^−1^ in agreement with computations, which broadens at low frequencies due to the librations of the molecules on the surface of the cluster. The hyper‐Raman spectrum shows a large band at 825 cm^−1^, similar to the IR spectrum, and a sharp, prominent band at 515 cm^−1^.^[^ ^61^ ^]^ This 515 cm^−1^ band has no counterparts in IR, Raman, and INS. Tentatively, a small peak appears in the computed density of states at 495 cm^−1^, which, because of some third‐order effect of electric dipole moment, enhances its intensity.

**Figure 7:**
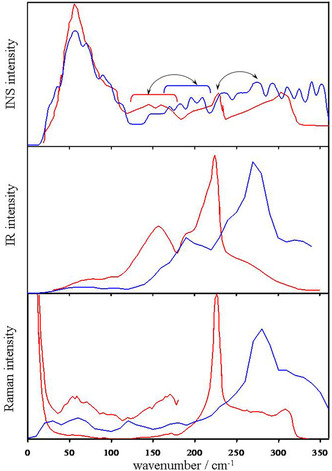
Computed vibrational spectra in the low frequency region for the hexagonal (H_2_O)_212_ cluster (blue) and experimental INS, IR, and Raman spectra for ice Ih (red). The measured spectra have been digitized from ref. [55] (Figure 4), [57] (Figure 1) and [59] (Figure 1).

The translational modes are classified in optical bands (130–330 cm^−1^ frequency region) and acoustic bands at lower frequencies, and in some degree, they are viewed as “hydrogen bond stretching modes”.^[^ ^28^ ^]^ The high‐resolution INS methodology is particularly useful in the optical region and three well‐defined bands (306 and 229 cm^−1^ sharp peaks and 153 cm^−1^ low intensity peak, **Figure** **7**) have been assessed,^[^ ^53^, ^54^, ^55^ ^]^ while IR and Raman spectra^[^ ^57^, ^59^ ^]^ show the sharp peak at 225 cm^−1^ and the other two absorptions appear as shoulders of the main peak. Instead, the hyper‐Raman spectrum shows two bands at 289 and 210 cm^−1^.^[^ ^61^ ^]^ The four vibrational spectroscopies provide complementary information unique to each technique that needs to be discussed individually. Present computed IR and Raman spectra reproduce the experimental band shape but computed frequencies are overestimated by about 50 cm^−1^, probably due to a slight overestimation of the HB strength in the M06‐2X/6‐31+G*/PCM theory level (Figure 7). Thus, the computed IR spectrum shows a sharp peak centered at 275 cm^−1^ with a shoulder at 240 cm^−1^ and one band at 210 cm^−1^ resembling the experimental features at 225, 190, and 161 cm^−1^, respectively. Computed Raman spectrum displays a sharp band at 280 cm^−1^ and moderate scattering intensity up to about 340 cm^−1^, in fair agreement with the experimental profile (225 and 315 cm^−1^, respectively). Computed INS spectrum shows broad absorptions from 150 to 400 cm^−1^ without a clear separation of various components as experimentally observed. A modest experimental/computational comparison has also been reported in previous calculations. Bowman et al.^[^ ^29^ ^]^ reproduced the experimental profile considering only the 105‐mer Ih‐core and projecting out the 87 surface molecules from their (H_2_O)_192_ cluster. Xantheas et al.^[^ ^28^ ^]^ results showed the two bands well‐separated with the calculated position of the higher‐energy peak close to the experiment, while the lower‐energy peak is overestimated by ≈37 cm^−1^. Several possible causes for the discrepancy have been considered, however, they proposed anharmonicity as a cause of this shortcoming. As far as the present study, it is likely that the density of states of larger clusters with a better surface‐area‐to‐volume ratio would allow for results that are more reliable.

It is very interesting to note that the sharp and intense acoustic band observed in the INS spectrum at 57 cm^−1^ is well reproduced^[^ ^55^ ^]^ through the computed phonon density of states, while this band is barely noticeable in the computed and experimental IR and Raman spectra (Figure 7).^[^ ^57^, ^59^ ^]^

## 4 Conclusions and Future Developments

In the present study, a step further toward the application of the bottom‐up/quantum chemical methodology to inquire into phenomena in water science is reported. Based on the “chemical structure intuition”, a new and simple approach has been proposed for the construction of low‐energy structures of hexagonal water clusters. This matter is complex given the enormous number of configurations that water molecules can assume and their energetic closeness. At present, the global minimum is known only for small clusters (*n* ≤ 55). Even though the ice Ih arrangement creates the strongest hydrogen bonding, the bi‐ and three‐coordinate molecules on the basal facet require a minimal surface reconstruction to preserve crystallinity and to get low energy minima.

Computations show that, when the size grows, all molecular properties smoothly converge toward experimental bulky counterparts. Thus, thermodynamic quantities (interaction energies and entropy), crystallographic parameters, and spectroscopic vibrational signatures of ice Ih are satisfactorily predicted. The reliability and the robustness of the approach require realistic molecular models and the need for a conspicuous number of computational resources.

This is not a conclusive point of arrival, now we are gathering new stimuli to obtain even important results in sectors, where experimental support is not available or hard to be interpreted, and molecular dynamics based on empirically derived potentials do not give poised and confident answers. There are several questions, closely related to the present study, that are desirable to be addressed. As an extension of the present study, it would be best to inquire molecular properties convergence by increasing the hexagonal cluster size with minimal basal surface reconstruction. In this context, the theory level improvement would also be valuable, and in particular, the basis set enlargement would benefit experimental/computational comparison.

As far as the water clusters themselves, it would be interesting to analyze surface relaxation (reconstruction) in order to get spheroidal structures as those prepared by rapid expansion of water vapor, and widely analyzed spectroscopically.^[^ ^9^ ^]^ This is also important in view of the possible formation of cubic ice (Ice Ic), the second solid phase that can be formed as microcrystals in our atmosphere.^[^ ^67^ ^]^

Another interesting development could concern the Ih transition to the ferroelectric structure ice‐XI, i.e., the proton ordered phase of ice Ih, which up to now is not entirely understood.^[^ ^60^ ^]^

## Conflict of Interest

The author declares no conflict of interest.

## Supporting information

Supplementary Material

## Data Availability

The data that support the findings of this study are available in the supplementary material of this article.
