# Supplementary material for: Quantifying Molecular Properties of Hexagonal Water Clusters
Source: ChemistryOpen. 2025 Jun 1;14(10):e202500149. doi: 10.1002/open.202500149 (PMC12518027; doi:10.1002/open.202500149)
Supplement: Supplementary file 1 — Supplementary Material [file OPEN-14-e202500149-s001.pdf]

Supporting Information:

Quantifying molecular properties of hexagonal water clusters

Giuseppe Lanza

Dipartimento di Scienze del Farmaco e della Salute, Università di Catania, Viale A. Doria 6, Catania, 95125 Italy

*E-mail: giuseppe.lanza@unict.it

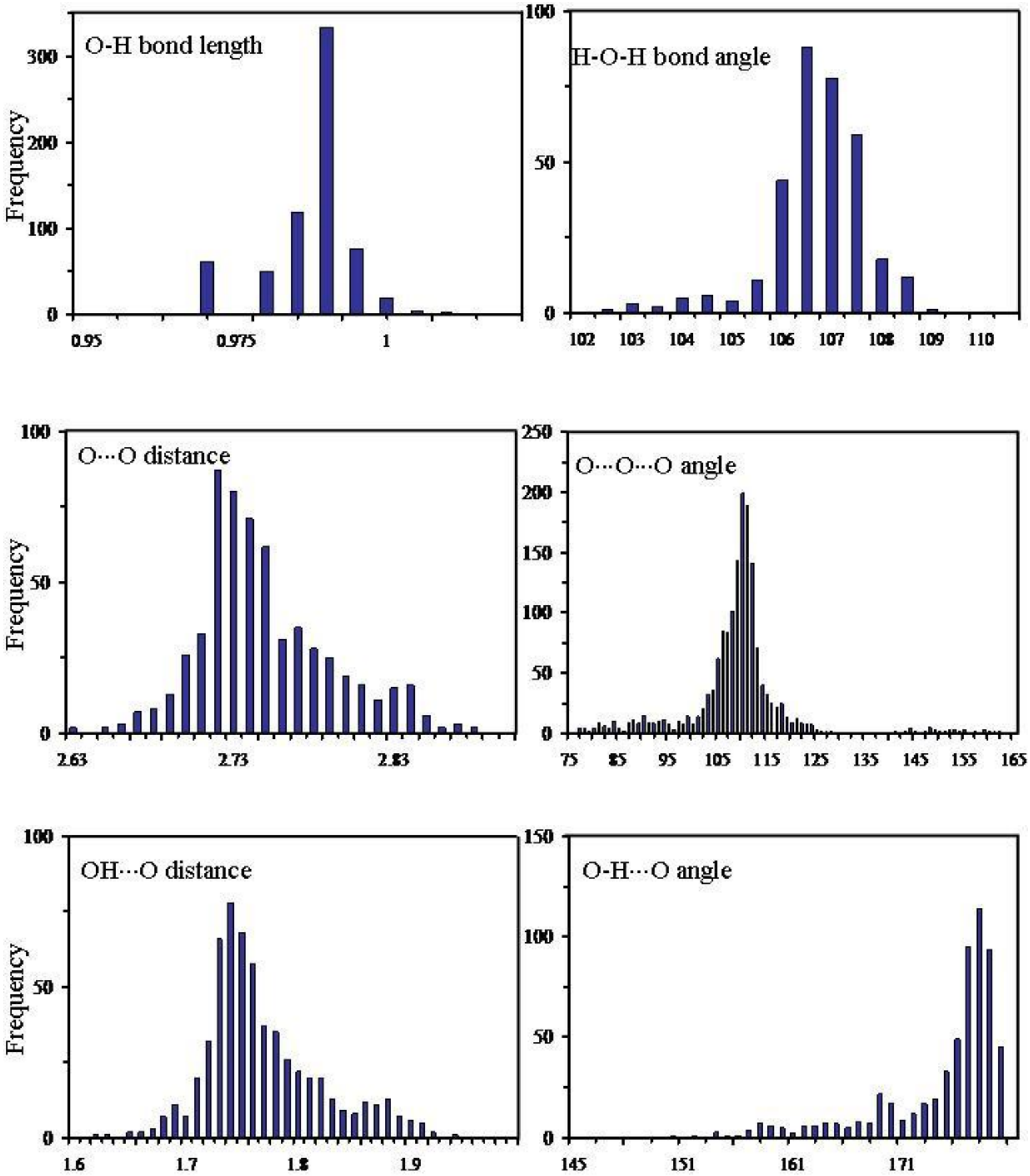

Figure S1. Computed geometrical parameter distributions for the $(H_2O)_{332}$ hexagonal cluster. Distances are in Å and angles are in degree.

Table S1. M06-2X/6-31+G*/PCM optimized Cartesian coordinates of the $(H_2O)_{96}$ hexagonal cluster.

O	-7.792403	-2.166299	2.908007
H	-7.285156	-1.379009	2.606355
H	-8.731531	-1.960971	2.789073
O	-5.322806	2.664116	5.142382
H	-5.734535	3.104505	4.372093
H	-4.424852	3.048158	5.222732
O	-6.218240	3.943450	2.764842
H	-6.949971	3.822864	2.123339
H	-5.800985	4.802471	2.499229
O	-4.726264	1.988358	1.621766
H	-5.138566	2.732745	2.122515
H	-3.742216	2.090954	1.659364
O	-5.780358	2.285538	-0.814372
H	-5.309148	2.198153	0.055087
H	-6.680278	2.582911	-0.566520
O	-4.966904	4.267210	-2.487132
H	-5.146519	3.509306	-1.879165
H	-4.002206	4.468780	-2.393798
O	-7.775420	3.780957	0.418715
H	-7.462537	4.604890	-0.021767
H	-8.742564	3.783172	0.363062
O	-5.320060	3.124177	-4.974846
H	-5.760569	3.762683	-5.555626
H	-5.235520	3.569640	-4.095495
O	-6.489381	-2.426149	5.415781
H	-5.986868	-1.586282	5.450245
H	-7.080086	-2.332798	4.640999
O	-5.023552	-0.073624	4.937270
H	-5.192400	0.891655	5.065160
H	-5.218389	-0.240488	3.983436
O	-5.772967	-0.421173	2.338285
H	-5.450774	0.452614	2.012475
H	-5.313663	-1.109673	1.800542
O	-4.851006	-2.480605	0.837459
H	-5.336220	-2.335649	-0.004722
H	-5.290157	-3.245006	1.287663
O	-5.942497	-1.947280	-1.623401
H	-5.419702	-1.148917	-1.892221
H	-6.788517	-1.854160	-2.106860
O	-4.754248	0.311406	-2.403389
H	-5.118604	1.021903	-1.828241
H	-3.764033	0.366932	-2.406651
O	-6.057030	0.412834	-4.739613
H	-6.024013	1.351909	-5.016023
H	-5.498479	0.395548	-3.921829
O	-5.031104	-1.667841	-6.266963
H	-5.368314	-0.872490	-5.797234
H	-5.419145	-2.434406	-5.796429
O	-6.385628	5.844012	-0.772716
H	-6.823432	6.611905	-1.169694
H	-5.863060	5.407877	-1.491767
O	-5.213137	-4.723729	4.517664
H	-5.520073	-3.890573	4.939535
H	-4.225939	-4.729012	4.551034
O	-6.283321	-4.406138	2.070739

H	-7.010779	-3.785370	2.283947
H	-5.849606	-4.602539	2.945570
O	-5.272628	-5.922074	-0.133319
H	-5.258202	-5.379359	-0.949159
H	-5.736047	-5.404720	0.554421
O	-7.767528	-1.458893	-3.696572
H	-7.366497	-0.671043	-4.133677
H	-8.728422	-1.336177	-3.716147
O	-4.966524	-4.336845	-2.473247
H	-5.225207	-3.439422	-2.150932
H	-5.452830	-4.404317	-3.321024
O	-6.429718	-3.627668	-4.765529
H	-6.948577	-4.228662	-5.321212
H	-7.064078	-2.964229	-4.409220
O	-5.186938	6.236838	1.745555
H	-5.473099	6.185053	0.810713
H	-4.210713	6.321986	1.744343
O	-1.334104	1.697264	6.697744
H	-1.567907	1.596315	7.632965
H	-0.332361	1.715540	6.659578
O	-2.703918	3.705992	5.470868
H	-2.178464	3.018426	5.951060
H	-2.747719	4.469657	6.066791
O	-1.326360	4.302488	3.138859
H	-1.815712	4.147754	3.979258
H	-1.705914	5.112658	2.727747
O	-2.038997	2.176193	1.572326
H	-1.723188	2.954491	2.090941
H	-1.762267	2.304500	0.631053
O	-1.342812	2.474814	-1.044435
H	-1.607979	1.677287	-1.564393
H	-0.353627	2.503419	-1.095355
O	-2.289153	4.754343	-2.198572
H	-1.935793	3.917787	-1.805333
H	-1.906797	4.818082	-3.116230
O	-1.382395	4.934836	-4.713225
H	-1.785917	4.177311	-5.201400
H	-0.410511	4.856746	-4.802452
O	-2.664334	2.846743	-5.932099
H	-3.579578	2.851541	-5.583499
H	-2.247815	2.004411	-5.647384
O	-1.439209	-2.855921	6.160069
H	-1.745382	-1.982403	5.831584
H	-0.460919	-2.832398	6.147893
O	-2.333218	-0.407987	5.188827
H	-3.316990	-0.300382	5.150608
H	-1.988555	0.333655	5.735545
O	-1.435807	-0.259419	2.627972
H	-1.670215	0.627953	2.263230
H	-1.771136	-0.290675	3.558630
O	-2.145885	-2.352999	1.031393
H	-3.128931	-2.393611	0.958856
H	-1.932036	-1.585867	1.616358
O	-1.306999	-2.126538	-1.538479
H	-1.578238	-2.184135	-0.588356
H	-0.317102	-2.156475	-1.543220
O	-2.074453	0.296834	-2.512408
H	-1.774098	-0.581936	-2.169638

H	-1.793392	0.351068	-3.459432
O	-1.360748	0.515060	-5.132238
H	-1.679106	-0.260557	-5.649019
H	-0.378419	0.538871	-5.221926
O	-2.374783	-1.649221	-6.524478
H	-3.368016	-1.665866	-6.432174
H	-2.194489	-1.586683	-7.474698
O	-1.392583	6.769680	-0.661784
H	-1.733939	6.041490	-1.249448
H	-1.695392	7.600209	-1.059875
O	-2.481302	-4.870162	4.606782
H	-2.080676	-4.146774	5.142887
H	-2.065330	-4.823283	3.718677
O	-1.352854	-4.772299	2.052455
H	-1.620215	-3.895714	1.686521
H	-1.829222	-5.449227	1.504810
O	-2.669640	-6.602954	0.551206
H	-3.594138	-6.341329	0.333521
H	-2.203075	-6.682416	-0.307222
O	-1.313252	-6.623472	-1.900220
H	-1.607565	-7.326918	-2.498543
H	-1.640143	-5.771876	-2.295150
O	-2.249440	-4.297317	-2.888234
H	-3.230573	-4.304373	-2.773406
H	-1.915029	-3.519307	-2.375706
O	-1.289350	-4.000409	-5.471549
H	-1.673733	-3.173836	-5.835042
H	-1.657879	-4.102412	-4.565311
O	-2.389143	6.519521	1.867961
H	-2.012419	6.622106	0.958384
H	-2.177344	7.340559	2.338078
O	1.307471	1.655742	6.617749
H	1.761208	2.446033	6.253125
H	1.629740	0.892478	6.085269
O	2.693698	3.784087	5.464774
H	2.235892	3.983171	4.606552
H	2.690415	4.609225	5.972744
O	1.429277	4.217751	3.113944
H	0.448849	4.280644	3.190326
H	1.605476	3.451201	2.514901
O	2.006844	2.124685	1.476820
H	1.708407	2.217489	0.539552
H	1.699773	1.247318	1.814879
O	1.353618	2.475013	-1.138310
H	1.738824	3.293896	-1.537824
H	1.644835	1.707107	-1.690838
O	2.348137	4.778600	-2.203056
H	1.978222	4.900724	-3.105972
H	2.010362	5.530513	-1.644119
O	1.429014	4.927342	-4.820234
H	1.899971	4.182580	-5.268477
H	1.728847	5.736413	-5.262031
O	2.795186	2.828689	-5.918749
H	2.892504	2.917490	-6.879344
H	3.718977	2.864253	-5.543080
O	1.388913	-2.878341	6.152003
H	1.729032	-3.012053	7.049832
H	1.726141	-3.635700	5.615652

O	2.249795	-0.452296	5.108764
H	1.956419	-1.318020	5.470991
H	1.894456	-0.397635	4.187610
O	1.268574	-0.250853	2.572469
H	0.279953	-0.290232	2.611494
H	1.556887	-1.020157	2.022081
O	2.127441	-2.340122	1.052570
H	1.850106	-3.206960	1.434821
H	3.115029	-2.304617	1.113044
O	1.398886	-2.140143	-1.560242
H	1.690899	-2.190567	-0.616354
H	1.661258	-1.250442	-1.903750
O	2.086154	0.293349	-2.571058
H	1.795433	0.366007	-3.513760
H	3.075524	0.268125	-2.594771
O	1.392090	0.531862	-5.196645
H	1.871534	1.333579	-5.503192
H	1.806504	-0.243308	-5.658597
O	2.507882	-1.607503	-6.413439
H	2.118405	-2.460162	-6.124248
H	3.477133	-1.683391	-6.287920
O	1.436872	6.770934	-0.656001
H	0.459186	6.759774	-0.605716
H	1.776745	6.667882	0.267084
O	2.311590	-4.949006	4.603033
H	2.017737	-5.799168	4.965355
H	1.934127	-4.897172	3.682364
O	1.358868	-4.760082	2.104090
H	0.371804	-4.844143	2.045866
H	1.756410	-5.459462	1.534839
O	2.652532	-6.608654	0.512458
H	2.611990	-7.501499	0.888356
H	2.170017	-6.649280	-0.366983
O	1.476340	-6.594374	-1.846754
H	0.494489	-6.601460	-1.838915
H	1.744653	-5.752502	-2.282308
O	2.353157	-4.249129	-2.986044
H	1.999853	-3.465504	-2.497021
H	2.039478	-4.168211	-3.916053
O	1.374306	-4.017648	-5.558631
H	0.375433	-4.012143	-5.528612
H	1.623927	-4.761921	-6.126720
O	2.458256	6.493660	1.857603
H	2.111074	5.692459	2.305586
H	3.438485	6.396085	1.834824
O	7.717441	-2.119007	2.892449
H	7.229067	-1.334217	2.572773
H	7.349269	-2.888360	2.416127
O	5.332290	2.774446	5.269524
H	4.408899	3.095169	5.340594
H	5.278214	1.796622	5.275530
O	6.074177	3.826670	2.824957
H	5.969686	3.521472	3.756340
H	5.464483	3.237684	2.317266
O	4.699058	2.084595	1.310339
H	3.710224	2.088458	1.345586
H	4.982750	2.174418	0.367065
O	5.830264	2.273574	-1.117007

H	5.496724	3.047121	-1.632526
H	5.523848	1.475199	-1.610264
O	5.044432	4.479855	-2.516012
H	4.086247	4.660944	-2.356092
H	5.557405	5.128888	-1.985469
O	7.649168	3.666009	0.497466
H	7.291903	3.660290	1.410188
H	7.158507	2.977133	0.004285
O	5.271154	3.029722	-4.916219
H	5.299729	3.530401	-4.074088
H	5.740117	2.181604	-4.782275
O	6.376740	-2.371277	5.321608
H	6.941692	-2.293392	4.519556
H	5.830125	-3.166924	5.161228
O	4.968510	-0.036629	5.130192
H	4.001890	-0.231756	5.140946
H	5.432407	-0.882486	5.347300
O	5.833650	-0.056313	2.538913
H	5.474942	0.731412	2.062784
H	5.482291	0.002802	3.460876
O	4.816992	-2.130787	1.160581
H	5.110905	-1.364519	1.717335
H	5.236289	-2.939060	1.545043
O	5.843133	-1.996238	-1.355492
H	5.500882	-2.840590	-1.739034
H	5.542539	-1.992487	-0.413542
O	4.777379	0.187884	-2.515218
H	5.092083	-0.658102	-2.106782
H	5.225868	0.250007	-3.391248
O	6.326522	0.410055	-4.746249
H	7.063824	-0.033045	-4.276386
H	5.986316	-0.250229	-5.392636
O	5.309595	-1.707270	-6.201778
H	5.669675	-1.852038	-7.090069
H	5.714175	-2.406952	-5.621308
O	6.561974	5.856963	-0.646859
H	7.080630	5.102265	-0.261428
H	7.204832	6.536020	-0.900664
O	5.064114	-4.769325	4.554933
H	4.081028	-4.851541	4.579965
H	5.409863	-5.526959	5.050808
O	6.139869	-4.328186	2.039124
H	5.742374	-4.637209	2.883260
H	5.900973	-4.976475	1.339995
O	5.242010	-5.909832	-0.090065
H	4.323227	-6.192090	0.144945
H	5.743310	-6.728707	-0.225925
O	7.825441	-1.267660	-3.070828
H	8.759687	-1.245393	-2.815607
H	7.305964	-1.444167	-2.253323
O	5.022613	-4.361174	-2.435492
H	5.128115	-4.984629	-1.683708
H	4.061137	-4.346405	-2.664405
O	6.599574	-3.355426	-4.517779
H	7.164655	-2.718296	-4.033725
H	6.077295	-3.824734	-3.833544
O	5.211839	6.275030	1.825264
H	5.517848	5.456311	2.275763

H	5.612472	6.241925	0.933115

Table S2. M06-2X/6-31+G*/PCM optimized Cartesian coordinates of the $(H_2O)_{102}$ hexagonal cluster.

O	-7.749298	-1.615109	3.438797
H	-7.326848	-0.870540	2.956869
H	-8.707025	-1.500048	3.348073
O	-5.231512	3.613835	4.543135
H	-5.651102	3.875412	3.706069
H	-4.318072	3.972157	4.526507
O	-6.518970	4.628140	1.974824
H	-7.129114	4.246158	1.294653
H	-7.072897	5.197251	2.533805
O	-4.847320	2.440341	1.266536
H	-5.173244	3.278732	1.635968
H	-3.854201	2.487238	1.238051
O	-5.928771	1.951474	-1.151748
H	-5.454972	2.169172	-0.311453
H	-6.846100	2.242633	-0.978725
O	-5.144062	3.508034	-3.238317
H	-5.389787	2.914826	-2.486737
H	-5.645807	4.327224	-3.043862
O	-7.878006	3.692144	-0.222034
H	-7.621179	4.372107	-0.889159
H	-8.846850	3.675394	-0.196381
O	-5.364076	1.970843	-5.532073
H	-5.821488	2.457293	-6.234443
H	-5.355238	2.571270	-4.748373
O	-6.351051	-1.357319	5.895175
H	-5.877498	-0.516039	5.731271
H	-6.971143	-1.441269	5.142338
O	-5.027068	0.880551	4.844672
H	-5.172091	1.856415	4.818816
H	-5.292530	0.562971	3.947430
O	-5.904314	0.131327	2.375844
H	-5.612582	0.946785	1.910971
H	-5.423962	-0.625574	1.961772
O	-4.924828	-2.170102	1.336132
H	-5.430622	-2.250484	0.498297
H	-5.300822	-2.845353	1.955254
O	-6.083374	-2.265679	-1.159467
H	-5.542384	-1.560885	-1.599346
H	-6.912047	-2.293217	-1.679837
O	-4.846759	-0.245247	-2.381905
H	-5.196330	0.550235	-1.922793
H	-3.857487	-0.192643	-2.432419
O	-6.101425	-0.633982	-4.719613
H	-6.054766	0.218289	-5.200107
H	-5.579260	-0.462829	-3.896029
O	-5.011377	-2.982364	-5.746047
H	-5.355337	-2.107132	-5.459288
H	-5.405583	-3.634139	-5.130050
O	-6.637015	5.470121	-1.918627

H	-7.144791	6.176515	-2.346360
H	-6.064508	5.907697	-1.245536
O	-5.007358	-3.725376	5.447545
H	-5.337164	-2.835510	5.708647
H	-4.028609	-3.703755	5.483846
O	-6.144705	-3.905581	3.002517
H	-6.907340	-3.304725	3.132558
H	-5.674974	-3.918164	3.877953
O	-5.108734	-5.834602	1.139700
H	-5.162524	-5.469561	0.231734
H	-5.557564	-5.200673	1.732754
O	-7.823659	-2.281220	-3.358241
H	-7.420031	-1.598091	-3.943579
H	-8.785003	-2.192316	-3.440302
O	-5.003154	-4.731059	-1.465115
H	-5.334876	-3.808604	-1.336193
H	-5.451989	-5.004304	-2.291928
O	-6.414687	-4.598702	-3.887764
H	-6.905809	-5.322226	-4.305553
H	-7.071994	-3.889883	-3.700386
O	-5.207174	6.417749	0.255239
H	-5.589605	5.829125	0.944777
H	-5.512867	7.311458	0.477984
O	-1.221831	2.683072	6.240031
H	-1.381998	2.244096	7.101331
H	-0.236034	2.719514	6.135433
O	-2.577271	4.559528	4.717099
H	-2.098085	3.943129	5.319664
H	-2.606921	5.409026	5.183787
O	-1.313780	4.792667	2.191469
H	-1.715327	5.493080	1.618539
H	-1.763870	4.811346	3.063951
O	-2.184327	2.452384	1.095411
H	-1.792818	3.289812	1.445352
H	-1.875680	2.331193	0.161660
O	-1.359693	2.004921	-1.426309
H	-1.632354	1.150767	-1.846192
H	-0.371856	2.052418	-1.503319
O	-2.448145	3.956033	-2.889333
H	-2.087957	3.210624	-2.344445
H	-3.420547	3.821888	-2.978655
O	-1.466234	3.987904	-5.413598
H	-1.858372	3.198477	-5.860247
H	-1.770757	3.912709	-4.476330
O	-2.652547	1.688323	-6.347868
H	-3.583041	1.707364	-6.042706
H	-2.240180	0.888294	-5.957172
O	-1.376186	-1.347717	6.526122
H	-1.760471	-0.738592	5.849158
H	-1.665458	-2.252833	6.274915
O	-2.344506	0.582726	4.913261
H	-3.329797	0.698728	4.925904
H	-1.944717	1.393643	5.307996
O	-1.408905	0.243744	2.440772
H	-1.695279	1.044603	1.935907
H	-1.754625	0.370355	3.361198
O	-2.209204	-2.149234	1.507804
H	-3.192142	-2.167407	1.424166

H	-1.968560	-1.256201	1.862705
O	-1.300869	-2.260068	-1.015955
H	-1.603158	-2.180392	-0.075213
H	-0.310013	-2.287107	-0.997251
O	-2.180562	-0.273614	-2.596996
H	-1.838552	-1.031776	-2.058319
H	-1.885536	-0.406936	-3.532023
O	-1.416899	-0.579008	-5.198282
H	-1.715639	-1.457647	-5.525100
H	-0.430181	-0.581208	-5.241150
O	-2.354652	-3.087068	-6.024828
H	-3.346511	-3.055589	-5.926992
H	-2.184792	-3.223995	-6.969338
O	-1.414626	6.359034	-2.198436
H	-1.788687	5.455829	-2.339749
H	-1.463781	6.746488	-3.102132
O	-2.196296	-3.836873	5.540860
H	-1.855697	-4.023922	4.627775
H	-1.939815	-4.597453	6.083955
O	-1.308161	-4.267539	2.997132
H	-1.616012	-3.490673	2.470112
H	-1.701628	-5.061434	2.550520
O	-2.453674	-6.393759	1.732904
H	-3.395273	-6.189021	1.527188
H	-2.036988	-6.648522	0.884583
O	-1.206904	-6.769798	-0.795400
H	-1.349449	-7.377874	-1.548260
H	-1.558523	-5.909394	-1.139172
O	-2.305237	-4.573954	-1.893323
H	-3.282195	-4.600365	-1.748985
H	-1.966917	-3.705359	-1.555177
O	-1.383674	-5.115588	-4.363452
H	-1.732945	-4.486912	-5.033977
H	-1.747130	-4.812018	-3.494728
O	-2.384488	6.616698	0.450889
H	-2.024895	6.635669	-0.460057
H	-3.352641	6.509801	0.354855
O	1.485255	2.708403	6.092611
H	1.869995	3.516227	5.686443
H	1.811574	1.953510	5.541190
O	2.651754	4.775044	4.624069
H	2.206295	4.814979	3.738017
H	2.634104	5.677277	4.977085
O	1.453515	4.790638	2.176540
H	0.470431	4.832455	2.230397
H	1.659637	3.923625	1.747465
O	2.098431	2.424162	0.992479
H	1.775273	2.307885	0.063799
H	1.792625	1.627067	1.492946
O	1.316778	2.075524	-1.561500
H	1.704162	2.790636	-2.127455
H	1.642468	1.218133	-1.936484
O	2.359871	4.033264	-3.071698
H	1.935954	3.959196	-3.961175
H	2.000526	4.871303	-2.683991
O	1.244623	4.088545	-5.558078
H	1.427014	5.047917	-5.618211
H	0.258088	4.004230	-5.509765

O	2.558556	1.635377	-6.210683
H	2.121702	2.495181	-6.049286
H	3.495766	1.743682	-5.928000
O	1.368219	-1.240532	6.623781
H	0.387136	-1.297915	6.527210
H	1.510400	-0.579802	7.342351
O	2.408230	0.513888	4.813721
H	2.034021	-0.209378	5.371353
H	1.996407	0.421401	3.917677
O	1.281715	0.266617	2.391252
H	0.292332	0.230441	2.429372
H	1.594363	-0.607566	2.048411
O	2.250923	-2.091383	1.526983
H	1.975343	-2.854345	2.092041
H	3.237724	-2.022941	1.569005
O	1.383292	-2.283581	-1.012914
H	1.727271	-2.177259	-0.090663
H	1.687475	-1.506912	-1.547509
O	2.161518	-0.255833	-2.594553
H	1.832814	-0.386428	-3.519793
H	3.150608	-0.270914	-2.636064
O	1.334875	-0.573222	-5.159535
H	1.783510	0.185546	-5.610403
H	1.761854	-1.413319	-5.458396
O	2.477246	-2.985406	-5.817394
H	2.141712	-3.792695	-5.377552
H	3.454304	-3.012269	-5.743780
O	1.342819	6.361799	-2.139353
H	0.362635	6.311701	-2.038060
H	1.726788	6.529157	-1.237988
O	2.522033	-3.593738	5.579755
H	2.138082	-2.773495	5.953235
H	2.090520	-3.759246	4.713340
O	1.444964	-4.200095	3.062882
H	0.461487	-4.284404	3.019221
H	1.823765	-5.002288	2.639474
O	2.661035	-6.357142	1.744446
H	2.609187	-7.179728	2.255198
H	2.208347	-6.539723	0.876252
O	1.543273	-6.677249	-0.690154
H	0.557265	-6.650460	-0.646526
H	1.823645	-5.831006	-1.120452
O	2.458102	-4.508423	-2.029260
H	2.070341	-3.667757	-1.673796
H	2.081986	-4.637194	-2.933126
O	1.369866	-5.203947	-4.431304
H	0.390014	-5.089471	-4.395807
H	1.513369	-6.156671	-4.219431
O	2.386719	6.707085	0.332803
H	2.053639	6.045719	0.977355
H	3.370526	6.640535	0.355537
O	7.857969	-1.457341	3.163075
H	7.370303	-0.756891	2.685738
H	7.468108	-2.307900	2.882331
O	5.330659	3.840061	4.524143
H	4.399116	4.140843	4.566014
H	5.312961	2.880579	4.719989
O	6.054308	4.406598	1.914528

H	5.943782	4.296421	2.887699
H	5.484685	3.694720	1.532787
O	4.782536	2.329944	0.782263
H	3.793461	2.338409	0.828213
H	5.058941	2.240060	-0.162758
O	5.920230	2.045798	-1.634897
H	5.579988	2.695250	-2.296855
H	5.611630	1.162707	-1.950005
O	5.068692	3.910363	-3.433085
H	4.102913	4.046888	-3.281782
H	5.540274	4.677227	-3.039596
O	7.662104	3.814832	-0.322381
H	7.298249	3.987611	0.571052
H	7.202877	3.018923	-0.659625
O	5.215578	1.978602	-5.489300
H	5.307716	2.637275	-4.771903
H	5.694232	1.171573	-5.216611
O	6.569354	-1.151380	5.619880
H	7.121475	-1.250451	4.811836
H	6.022318	-1.964241	5.643493
O	5.099929	1.041921	4.926977
H	4.146192	0.793688	4.916066
H	5.588975	0.277164	5.320506
O	5.991378	0.512974	2.402062
H	5.609145	1.175686	1.777378
H	5.652239	0.762625	3.296393
O	4.932175	-1.803374	1.531575
H	5.234502	-0.938031	1.909479
H	5.355216	-2.513915	2.073917
O	5.981343	-2.158345	-0.959600
H	5.645573	-3.068266	-1.152647
H	5.679322	-1.962488	-0.039091
O	4.852562	-0.291264	-2.549248
H	5.187472	-1.023922	-1.973159
H	5.280401	-0.419486	-3.428929
O	6.320728	-0.567504	-4.826489
H	7.079600	-0.897276	-4.301453
H	5.961644	-1.353845	-5.297740
O	5.288329	-2.964234	-5.746154
H	5.618899	-3.291190	-6.596696
H	5.730140	-3.515972	-5.045735
O	6.528668	5.696441	-1.893354
H	7.065288	5.049629	-1.363746
H	7.154447	6.321531	-2.288778
O	5.189258	-3.599947	5.406529
H	4.194271	-3.586587	5.472691
H	5.494077	-4.253344	6.053831
O	6.228221	-3.775913	2.857819
H	5.830230	-3.870980	3.753071
H	5.958770	-4.554614	2.322770
O	5.278606	-5.751665	1.102783
H	4.355163	-5.988372	1.363636
H	5.779329	-6.581742	1.126957
O	7.894845	-1.829479	-2.869602
H	8.837865	-1.753344	-2.661275
H	7.408094	-1.813598	-2.014310
O	5.120381	-4.683716	-1.505041
H	5.199942	-5.145682	-0.641914

H	4.156870	-4.645396	-1.721784
O	6.659739	-4.194992	-3.789950
H	7.224032	-3.460666	-3.470412
H	6.149091	-4.498223	-3.010082
O	5.136217	6.568395	0.423765
H	5.452574	5.865218	1.034297
H	5.557791	6.369374	-0.436783
O	-1.381460	0.769837	8.309943
H	-1.514138	-0.096474	7.863718
H	-1.944517	0.767111	9.098546
O	1.426596	0.949888	8.313884
H	0.464543	0.961709	8.495071
H	1.577525	1.692957	7.695597
O	-1.348592	-7.724447	-3.471862
H	-1.906028	-8.400930	-3.884114
H	-1.495127	-6.890115	-3.973217
O	1.454774	-7.750762	-3.349311
H	0.494119	-7.929033	-3.421646
H	1.599166	-7.541644	-2.403492
O	-1.299461	6.802148	-4.950699
H	-0.331666	6.916490	-5.045982
H	-1.483298	5.903942	-5.290849
O	1.513522	6.812700	-4.849581
H	2.063566	7.527473	-5.203096
H	1.619568	6.826568	-3.870842

Table S3. M06-2X/6-31+G*/PCM optimized Cartesian coordinates of the $(H_2O)_{160}$ hexagonal cluster.

O	9.861912	-5.717366	0.229363
H	10.829124	-5.751610	0.191918
H	9.517284	-6.491890	-0.291176
O	7.284218	-5.072911	4.884165
H	7.780624	-5.441419	5.631120
H	7.726873	-5.416880	4.075498
O	9.583828	0.288674	3.489276
H	10.546109	0.297079	3.597331
H	9.180647	0.279793	4.397181
O	6.681524	2.548582	4.993539
H	6.899398	2.363123	4.051150
H	5.704629	2.448261	5.084372
O	8.081420	0.215519	5.718431
H	7.470740	0.974292	5.586274
H	7.554125	-0.605873	5.663869
O	6.778639	-2.280115	5.158245
H	6.996066	-3.236933	5.155640
H	5.790592	-2.223506	5.165301
O	7.830479	-1.728341	2.735921
H	7.423287	-1.840405	3.631886
H	8.633601	-1.179233	2.867843
O	6.794237	-3.939186	1.475135
H	7.230203	-3.163832	1.894455
H	7.220864	-4.736565	1.872364
O	8.194569	-6.058520	2.438781

H	8.962161	-5.917753	1.840954
H	7.778358	-6.895451	2.122011
O	7.165852	-8.323570	1.265055
H	7.537904	-9.143289	1.624266
H	6.171054	-8.431211	1.301975
O	8.542566	-7.656268	-1.081411
H	7.921560	-7.939339	-0.376924
H	8.011219	-7.213026	-1.774827
O	6.923322	5.416944	4.869480
H	6.885690	4.438563	4.923102
H	5.991421	5.729536	4.952302
O	7.903931	6.393989	2.508958
H	7.616956	6.151142	3.424708
H	7.468329	7.238775	2.256395
O	6.600461	4.315388	1.391728
H	7.015694	5.124622	1.782057
H	7.052931	3.551043	1.816354
O	7.683881	2.055815	2.488892
H	8.545750	1.658255	2.734711
H	7.218220	1.346224	1.976202
O	6.553645	0.032913	1.135043
H	6.885014	-0.694183	1.713889
H	5.565945	0.042765	1.208125
O	7.691611	0.013859	-1.355700
H	7.347406	0.025640	-0.431615
H	7.321905	-0.805406	-1.764879
O	6.819522	-2.228538	-2.594899
H	7.333708	-2.914123	-2.113487
H	7.222338	-2.131145	-3.493296
O	7.950622	-4.100113	-0.958984
H	8.814580	-4.467232	-0.674832
H	7.438897	-4.005415	-0.117925
O	7.056406	-5.943233	-2.761869
H	7.272159	-5.262028	-2.082370
H	7.222747	-5.495770	-3.628965
O	9.417539	0.324695	-3.607143
H	8.996957	0.338174	-2.725202
H	9.061559	1.101728	-4.084267
O	6.827707	8.670782	1.350510
H	7.157552	9.505032	1.716824
H	5.832014	8.745447	1.346949
O	8.193840	8.031918	-1.043836
H	8.763515	7.339634	-0.638318
H	7.627189	8.333168	-0.303368
O	6.804293	6.310055	-2.667789
H	7.275846	7.066215	-2.240548
H	7.037494	5.545007	-2.080327
O	7.603939	4.249997	-1.118926
H	7.118581	4.228986	-0.260122
H	7.300023	3.455500	-1.622106
O	6.587975	2.159672	-2.511031
H	6.893224	1.322748	-2.076575
H	5.598733	2.173233	-2.491860
O	7.824739	2.323173	-4.884388
H	7.273857	2.280354	-4.063054
H	7.803651	3.270063	-5.129755
O	6.956505	0.166344	-6.422767
H	7.213934	0.992178	-5.956813

H	5.976697	0.147258	-6.432003
O	8.138425	-1.775817	-4.917532
H	7.626449	-1.177415	-5.518068
H	8.766111	-1.157271	-4.476684
O	7.362904	-4.550863	-5.117086
H	7.759226	-3.658906	-5.081314
H	6.445736	-4.429700	-5.460635
O	9.489174	5.898947	0.197832
H	9.079138	5.949561	1.084262
H	9.028718	5.176063	-0.272893
O	7.166820	5.091299	-5.123816
H	7.109768	5.603590	-4.280724
H	7.603275	5.675667	-5.761609
O	3.302773	-4.392521	6.583384
H	3.673961	-4.384370	7.478863
H	3.799846	-5.099154	6.076605
O	4.662644	-6.157484	5.148027
H	5.551615	-5.788822	4.964863
H	4.217729	-6.294689	4.279421
O	2.935344	4.498392	6.678345
H	3.210119	4.500937	7.607557
H	1.948117	4.491805	6.680272
O	3.948249	2.333309	5.260515
H	3.590440	3.092432	5.772123
H	3.637024	1.511759	5.730661
O	3.093164	0.131358	6.528094
H	3.427177	-0.670494	6.068621
H	2.107819	0.076462	6.500471
O	4.063495	-2.111260	5.219113
H	3.740501	-2.910477	5.693765
H	3.679880	-2.141501	4.308111
O	3.054278	-2.129611	2.688474
H	3.316281	-1.306919	2.205259
H	2.064799	-2.158251	2.685765
O	4.079681	-4.244871	1.343423
H	5.054081	-4.111162	1.413787
H	3.671789	-3.471086	1.807817
O	3.388736	-6.505172	2.720148
H	3.600217	-5.687934	2.207706
H	2.403249	-6.577621	2.740180
O	4.532583	-8.611953	1.367916
H	4.096514	-7.867223	1.843679
H	4.146475	-8.635035	0.466146
O	3.527020	-8.723805	-1.236110
H	3.854224	-9.527361	-1.668057
H	3.901645	-7.963743	-1.750239
O	4.348920	6.374151	5.120605
H	3.799264	5.770615	5.659783
H	3.869461	6.497930	4.271601
O	3.079050	6.704299	2.670249
H	3.339494	5.898228	2.162163
H	2.094073	6.719715	2.683364
O	3.891087	4.465942	1.344506
H	4.877274	4.408846	1.353933
H	3.608833	4.455997	0.396528
O	3.020714	2.289662	2.699858
H	3.366056	2.330061	3.626490
H	3.336502	3.102426	2.231542

O	3.866103	0.083652	1.350949
H	3.556519	0.905416	1.805954
H	3.528997	0.100199	0.420962
O	3.089056	0.004950	-1.244137
H	3.455140	-0.803560	-1.680564
H	2.101922	-0.062405	-1.279056
O	4.099400	-2.193058	-2.497667
H	5.085695	-2.234443	-2.506178
H	3.792992	-3.024645	-2.057580
O	3.267585	-4.458082	-1.249912
H	3.588434	-4.422822	-0.315015
H	3.690827	-5.236232	-1.686440
O	4.409990	-6.553701	-2.587845
H	5.379429	-6.360952	-2.662422
H	4.055342	-6.585888	-3.506058
O	3.407219	-6.668091	-5.161762
H	3.700743	-7.467753	-5.624162
H	2.418377	-6.672855	-5.206958
O	4.165878	8.867765	1.356844
H	3.753762	8.104771	1.824330
H	3.787464	8.872543	0.451773
O	3.102231	8.863775	-1.235879
H	3.502797	8.087457	-1.709648
H	3.383147	9.651238	-1.726234
O	4.094221	6.691378	-2.481954
H	5.075272	6.575708	-2.522176
H	3.733149	5.882778	-2.042030
O	3.059623	4.490553	-1.250009
H	3.335410	3.665191	-1.720946
H	2.071161	4.507059	-1.275372
O	3.885183	2.245946	-2.527973
H	3.556435	1.415367	-2.101543
H	3.620107	2.220541	-3.480129
O	3.266927	2.329171	-5.176229
H	3.617025	1.545579	-5.658211
H	3.748947	3.130521	-5.516412
O	4.160046	0.042832	-6.445292
H	3.860605	-0.770321	-5.956682
H	3.821428	-0.044450	-7.349076
O	3.370697	-2.163476	-5.122274
H	3.605529	-2.145205	-4.161594
H	2.385875	-2.236221	-5.164978
O	4.781607	-4.368769	-6.066650
H	4.273228	-5.157515	-5.788848
H	4.264172	-3.593771	-5.755693
O	3.202492	6.755103	-5.114887
H	3.465870	6.766435	-4.168911
H	2.217172	6.731251	-5.127977
O	4.522374	4.550174	-6.033856
H	5.436505	4.690762	-5.711133
H	4.011009	5.345094	-5.742131
O	0.568997	-4.459921	6.614820
H	1.557632	-4.460619	6.613393
H	0.302840	-4.477846	7.546564
O	-0.429399	-6.650708	5.176966
H	-0.103262	-5.891540	5.703472
H	-1.408954	-6.604688	5.194171
O	0.192946	4.453546	6.567068

H	-0.210604	4.397096	7.447074
H	-0.117891	3.646778	6.066464
O	-0.634236	2.302398	5.221650
H	-0.309354	2.286859	4.287241
H	-1.622970	2.287691	5.181648
O	0.348253	0.025393	6.495251
H	-0.023616	0.804318	6.027493
H	0.013770	-0.769802	6.027043
O	-0.529961	-2.308457	5.229907
H	-0.167351	-3.072675	5.730839
H	-1.515045	-2.380901	5.256131
O	0.351654	-2.258170	2.652484
H	0.004422	-2.315153	3.576676
H	0.089661	-3.088375	2.183890
O	-0.323088	-4.536756	1.313604
H	0.045724	-5.325209	1.787058
H	0.021988	-4.561468	0.387720
O	0.647912	-6.672197	2.685304
H	0.242482	-6.671266	3.589593
H	0.312610	-7.483408	2.221377
O	-0.275640	-8.832574	1.345476
H	0.058503	-8.820653	0.416699
H	-1.253072	-8.839188	1.292452
O	0.701986	-8.812439	-1.215001
H	1.678006	-8.732615	-1.240321
H	0.343333	-8.067043	-1.744442
O	-0.646916	6.702716	5.255846
H	-0.329794	5.906500	5.748053
H	-0.317397	7.470843	5.746827
O	0.311774	6.706226	2.655815
H	-0.045412	6.723669	3.572911
H	-0.058025	7.492041	2.191326
O	-0.513226	4.454952	1.309446
H	-0.206556	5.249244	1.809038
H	-0.194080	3.660743	1.806168
O	0.314976	2.245107	2.665804
H	1.305167	2.248748	2.689222
H	0.051382	1.418667	2.190507
O	-0.442274	-0.010178	1.326894
H	-0.146704	-0.823882	1.804267
H	-1.431741	-0.037590	1.343108
O	0.392107	-0.038026	-1.270909
H	0.064233	0.002037	-0.338593
H	0.095201	0.782715	-1.734960
O	-0.248426	-2.304073	-2.628695
H	0.007453	-1.501539	-2.111699
H	0.061265	-3.093824	-2.119147
O	0.549023	-4.527892	-1.276199
H	1.537383	-4.559522	-1.288817
H	0.229021	-5.315222	-1.781658
O	-0.365394	-6.691902	-2.672859
H	-0.033511	-6.693395	-3.599514
H	-1.352513	-6.698544	-2.717460
O	0.675765	-6.692073	-5.239551
H	0.372659	-7.473578	-5.726885
H	0.311859	-5.894795	-5.733572
O	-0.711541	8.871110	1.259859
H	-0.411382	9.699847	1.664324

H	-0.317697	8.856064	0.337526
O	0.293509	8.828899	-1.189135
H	1.273920	8.793909	-1.219846
H	-0.037747	8.066399	-1.717674
O	-0.649125	6.670140	-2.628444
H	-0.344993	5.864502	-2.144814
H	-1.631193	6.621169	-2.674090
O	0.329192	4.494265	-1.295283
H	0.017764	4.446640	-0.359039
H	0.057562	3.654634	-1.740159
O	-0.367371	2.208439	-2.623579
H	-0.016822	2.235584	-3.545413
H	-1.354032	2.190621	-2.702610
O	0.546665	2.300255	-5.227238
H	1.537179	2.322729	-5.241807
H	0.274339	1.471061	-5.682633
O	-0.311001	-0.014104	-6.507757
H	0.005983	-0.034516	-7.424027
H	-1.297657	-0.058457	-6.560892
O	0.638568	-2.292320	-5.206730
H	0.316082	-2.276467	-4.272328
H	0.290300	-1.481651	-5.639375
O	-0.257457	-4.577888	-6.498290
H	0.041119	-3.758625	-6.043722
H	-1.246732	-4.561210	-6.485518
O	0.447953	6.729468	-5.177162
H	0.054645	6.701757	-4.276684
H	0.119927	5.932789	-5.640851
O	-0.437319	4.429551	-6.552870
H	-0.066221	3.632781	-6.087718
H	-0.043845	4.431623	-7.438975
O	-4.550922	-4.622768	6.170055
H	-5.468557	-4.640992	5.809522
H	-4.128212	-3.800699	5.834243
O	-3.227599	-6.689198	5.168339
H	-3.721027	-5.923870	5.586639
H	-3.520034	-7.488309	5.632374
O	-4.677841	4.460838	6.259456
H	-4.217672	3.676606	5.890688
H	-4.198874	5.245590	5.903490
O	-3.362175	2.244472	5.164260
H	-3.659583	1.413739	5.597641
H	-3.648458	2.190041	4.219443
O	-4.180957	-0.080454	6.456872
H	-3.855182	-0.068607	7.370104
H	-5.168871	-0.073729	6.516287
O	-3.265635	-2.375538	5.177663
H	-3.602494	-1.554507	5.601157
H	-3.522601	-2.330857	4.223857
O	-3.996347	-2.352986	2.547212
H	-3.628193	-3.145634	2.082876
H	-4.978062	-2.450811	2.524382
O	-3.039112	-4.549898	1.259251
H	-2.049231	-4.567262	1.256657
H	-3.329107	-4.515753	0.314400
O	-4.072661	-6.706552	2.554612
H	-3.732991	-6.699062	3.481281
H	-3.676290	-5.920918	2.100338

O	-3.110172	-8.885257	1.285098
H	-3.414715	-9.668765	1.768105
H	-3.480692	-8.100786	1.770931
O	-4.034326	-8.892670	-1.288328
H	-3.766301	-9.726640	-1.703229
H	-3.688565	-8.920786	-0.362169
O	-3.468321	6.669327	5.159406
H	-2.490176	6.682127	5.187648
H	-3.718131	6.629859	4.209890
O	-4.322368	6.510659	2.525859
H	-3.905955	5.752795	2.046454
H	-4.059349	7.321258	2.038812
O	-3.230053	4.378862	1.230859
H	-2.241155	4.413523	1.240214
H	-3.510441	4.341333	0.284223
O	-4.104788	2.159570	2.544071
H	-3.789486	2.964774	2.063846
H	-5.091322	2.154846	2.478589
O	-3.148012	-0.067331	1.319287
H	-3.489218	-0.876497	1.773703
H	-3.517454	0.720806	1.789838
O	-3.820418	-0.086956	-1.305485
H	-3.512324	-0.039092	-0.366710
H	-3.518272	0.731754	-1.769691
O	-2.954689	-2.274704	-2.660872
H	-1.964323	-2.284953	-2.654187
H	-3.233788	-1.472116	-2.154445
O	-3.888444	-4.447429	-1.334509
H	-3.527542	-3.658580	-1.811396
H	-4.871153	-4.364548	-1.386623
O	-3.111292	-6.680234	-2.690791
H	-3.426285	-7.464854	-2.188736
H	-3.395243	-5.887328	-2.172244
O	-4.404013	-6.433614	-5.146549
H	-4.004523	-6.536999	-4.254981
H	-5.284701	-6.025465	-5.024019
O	-3.445032	8.809365	1.182517
H	-2.455414	8.845235	1.213121
H	-3.755954	9.606217	1.639573
O	-4.462352	8.696653	-1.356985
H	-4.068314	8.764553	-0.453293
H	-4.278028	9.542077	-1.794022
O	-3.417656	6.533047	-2.719007
H	-3.805201	7.322801	-2.276533
H	-3.836610	6.462502	-3.605591
O	-4.073258	4.255098	-1.366877
H	-3.860510	5.094902	-1.842104
H	-5.055527	4.152204	-1.397819
O	-3.072275	2.127117	-2.702888
H	-3.440100	2.928325	-2.251972
H	-3.428093	2.122679	-3.623118
O	-4.012290	2.098290	-5.284995
H	-3.677997	1.303499	-5.762545
H	-3.674952	2.879301	-5.779863
O	-3.047880	-0.138254	-6.571165
H	-3.388034	-0.935153	-6.075293
H	-3.389882	-0.214205	-7.475302
O	-3.922618	-2.248061	-5.196194

H	-3.568387	-2.245783	-4.271571
H	-4.905240	-2.300304	-5.119028
O	-2.982882	-4.515189	-6.517904
H	-3.443009	-5.252075	-6.052192
H	-3.302787	-3.700613	-6.073586
O	-4.628205	6.164624	-5.208652
H	-4.670371	7.005028	-5.691179
H	-4.049454	5.571720	-5.747262
O	-3.150081	4.355534	-6.632535
H	-2.159991	4.407231	-6.625834
H	-3.414260	4.325328	-7.564996
O	-9.429884	-0.100472	3.662189
H	-9.038553	-0.049174	2.768588
H	-9.121565	0.697296	4.136952
O	-7.113777	-4.847961	5.186411
H	-7.568288	-3.995753	5.044100
H	-7.069631	-5.313103	4.323066
O	-9.746144	5.638099	-0.200415
H	-9.420291	6.446646	0.247373
H	-9.379750	5.679679	-1.105071
O	-7.880903	1.936318	4.941275
H	-7.394781	1.927966	4.079627
H	-7.796680	2.879840	5.225022
O	-6.904011	-0.060232	6.455305
H	-7.304966	-0.023523	7.337315
H	-7.247269	0.732793	5.958821
O	-8.016777	-2.124550	4.918415
H	-7.520374	-1.543613	5.534906
H	-8.674642	-1.512436	4.510253
O	-6.719875	-2.472315	2.558242
H	-7.211803	-3.184876	2.091490
H	-7.109120	-2.392399	3.462709
O	-7.774996	-4.436646	0.990693
H	-8.575277	-4.913800	0.667976
H	-7.236235	-4.332893	0.167342
O	-6.794074	-6.264047	2.746699
H	-5.820991	-6.409645	2.672214
H	-7.023480	-5.564275	2.088469
O	-8.030914	-8.087095	1.115944
H	-7.571985	-7.549581	1.808390
H	-8.431709	-8.844650	1.567462
O	-6.817611	-8.505193	-1.394431
H	-5.848612	-8.638585	-1.338627
H	-7.141504	-8.420812	-0.474074
O	-7.265106	4.582675	5.340979
H	-6.335586	4.547540	5.673672
H	-7.218817	5.058356	4.485389
O	-7.011705	5.947769	2.820084
H	-6.054620	6.161691	2.714052
H	-7.201263	5.211417	2.191336
O	-7.887107	4.041668	1.062869
H	-7.361379	3.979658	0.227937
H	-8.724593	4.460309	0.755721
O	-6.804034	1.973266	2.471064
H	-7.303471	2.683954	2.007565
H	-7.087778	1.122663	2.045844
O	-7.736913	-0.268851	1.346086
H	-7.300362	-1.063580	1.737319

H	-7.369041	-0.180638	0.436171
O	-6.511319	0.003714	-1.096525
H	-5.523134	-0.011178	-1.163279
H	-6.834466	0.736568	-1.671864
O	-7.644576	-1.971070	-2.503262
H	-8.507061	-1.570262	-2.740852
H	-7.173180	-1.274019	-1.976724
O	-6.604883	-4.266497	-1.430324
H	-7.040743	-3.478128	-1.826166
H	-7.025452	-5.051263	-1.865608
O	-7.867170	-6.328202	-2.617253
H	-7.398906	-7.156670	-2.347791
H	-7.643622	-6.119343	-3.548059
O	-6.984370	-5.226554	-5.067206
H	-7.430696	-5.490839	-5.885962
H	-6.926888	-4.242791	-5.099015
O	-8.388724	7.663323	1.199862
H	-7.887865	7.150957	1.883885
H	-8.843827	8.381574	1.663944
O	-7.233133	8.200567	-1.306881
H	-6.268821	8.369233	-1.277441
H	-7.530486	8.084123	-0.380822
O	-8.131805	5.971212	-2.549355
H	-7.732215	6.827156	-2.253841
H	-7.887954	5.813461	-3.485766
O	-6.777358	3.951521	-1.398451
H	-7.231726	4.729832	-1.812786
H	-7.182609	3.163871	-1.823419
O	-7.756033	1.751490	-2.740749
H	-7.338332	1.870235	-3.629237
H	-8.562093	1.214303	-2.894849
O	-6.738248	2.241390	-5.208539
H	-5.752667	2.172966	-5.249013
H	-6.946918	3.200345	-5.263899
O	-8.033712	-0.117491	-5.885015
H	-7.571699	0.748982	-5.801831
H	-8.471939	-0.109319	-6.749640
O	-6.657994	-2.445834	-4.980975
H	-6.930189	-2.249390	-4.049730
H	-7.088777	-1.738740	-5.505191
O	-9.512350	-6.147469	-0.282146
H	-9.139088	-6.936443	0.163167
H	-9.134232	-6.157281	-1.183248
O	-7.168412	5.044742	-5.050171
H	-6.270636	5.446628	-5.127795
H	-7.666444	5.372155	-5.815384
O	-9.533128	-0.237182	-3.568749
H	-10.500466	-0.229042	-3.619633
H	-9.193837	-0.235350	-4.493089

Tble S4. M06-2X/6-31+G*/PCM optimized Cartesian cordinates of the $(H_2O)_{172}$ heoxagonal cluster.

O	-10.555919	-4.406906	-0.459310
H	-11.523259	-4.359236	-0.469159

H	-10.304693	-5.205325	0.078555
O	-7.745459	-4.027799	-5.006060
H	-8.283838	-4.296957	-5.766216
H	-8.240498	-4.328259	-4.211081
O	-9.476127	1.584164	-3.769030
H	-10.421835	1.727780	-3.919665
H	-9.040778	1.499002	-4.657912
O	-6.206867	3.399088	-5.169579
H	-6.460760	3.227071	-4.233870
H	-5.270721	3.115694	-5.274963
O	-7.905871	1.272089	-5.928867
H	-7.216819	1.957788	-5.786567
H	-7.479487	0.398480	-5.822666
O	-6.891091	-1.308628	-5.213899
H	-7.187102	-2.243813	-5.231989
H	-5.906060	-1.324481	-5.133209
O	-8.033793	-0.614139	-2.871170
H	-7.591476	-0.785527	-3.740737
H	-8.753216	0.028109	-3.054408
O	-7.316626	-2.924465	-1.568946
H	-7.655760	-2.113417	-2.009448
H	-7.798553	-3.681214	-1.982645
O	-8.834739	-4.931624	-2.590521
H	-9.613156	-4.724068	-2.026899
H	-8.508481	-5.801746	-2.259453
O	-8.107450	-7.302252	-1.379327
H	-8.582141	-8.060429	-1.752746
H	-7.141888	-7.562934	-1.362782
O	-9.484563	-6.456803	0.906022
H	-8.873855	-6.814133	0.226644
H	-8.933173	-6.069071	1.616373
O	-6.023280	6.284441	-4.920244
H	-6.119445	5.313603	-5.012102
H	-5.054715	6.468678	-4.950718
O	-6.923987	7.415318	-2.590426
H	-6.651814	7.133850	-3.498638
H	-6.367185	8.177249	-2.314555
O	-6.030719	5.109805	-1.509511
H	-6.288893	5.992862	-1.873773
H	-6.580989	4.449960	-1.990467
O	-7.371757	3.060009	-2.714742
H	-8.271135	2.792678	-2.999284
H	-7.058314	2.316598	-2.138608
O	-6.625637	0.974597	-1.194966
H	-7.014586	0.284139	-1.782568
H	-5.641999	0.874471	-1.231624
O	-7.809782	1.075431	1.280803
H	-7.461154	1.064396	0.358778
H	-7.543045	0.208907	1.672264
O	-7.196068	-1.265832	2.485420
H	-7.800961	-1.873677	2.007110
H	-7.565575	-1.128932	3.392977
O	-8.577732	-2.944352	0.820164
H	-9.459026	-3.234253	0.502363
H	-8.024900	-2.914580	0.000521
O	-7.909057	-4.875633	2.627398
H	-8.070865	-4.164382	1.963490
H	-7.992698	-4.433811	3.507519

O	-9.434555	1.568009	3.588415
H	-9.037669	1.548080	2.695917
H	-8.983924	2.293398	4.066217
O	-5.577277	9.513924	-1.366025
H	-5.792035	10.383682	-1.735443
H	-4.582198	9.483989	-1.294595
O	-7.090752	9.020831	0.968204
H	-7.751804	8.438400	0.530080
H	-6.471814	9.259109	0.246670
O	-6.015906	7.030204	2.516538
H	-6.343573	7.872093	2.115058
H	-6.405082	6.334219	1.926087
O	-7.156531	5.149901	0.951340
H	-6.643317	5.055235	0.114088
H	-6.956852	4.345123	1.489064
O	-6.425515	3.028727	2.464856
H	-6.830339	2.234438	2.032016
H	-5.444071	2.904402	2.491619
O	-7.584344	3.347823	4.856534
H	-7.074668	3.235723	4.014650
H	-7.407149	4.276267	5.108574
O	-6.958156	1.083382	6.354046
H	-7.112913	1.940948	5.899589
H	-5.988797	0.941016	6.362676
O	-8.382847	-0.681284	4.849748
H	-7.792604	-0.150915	5.443102
H	-8.947335	0.008991	4.430445
O	-7.945285	-3.516264	5.031186
H	-8.207155	-2.574635	5.018085
H	-7.027079	-3.541350	5.374136
O	-8.669493	7.148288	-0.363954
H	-8.216961	7.147577	-1.231026
H	-8.361493	6.346533	0.102732
O	-6.481381	5.984031	5.031409
H	-6.373881	6.431422	4.156348
H	-6.850889	6.653321	5.626785
O	-3.637178	-3.607120	-6.411770
H	-3.821099	-2.838667	-5.818869
H	-4.219870	-4.334663	-6.078509
O	-5.282340	-5.457404	-5.264890
H	-6.129255	-4.994272	-5.102550
H	-4.907105	-5.675659	-4.382659
O	-2.239242	4.845245	-6.622682
H	-2.486580	5.015743	-7.544282
H	-1.253671	4.773679	-6.611322
O	-3.549644	2.668319	-5.529560
H	-3.064107	3.403125	-5.969004
H	-3.342530	1.849640	-6.033271
O	-2.794765	0.267384	-6.702516
H	-3.011356	-0.332624	-7.456147
H	-1.822873	0.154761	-6.565548
O	-4.181119	-1.351565	-4.997563
H	-3.663166	-0.677858	-5.498287
H	-3.805467	-1.396087	-4.081848
O	-3.230058	-1.572953	-2.506902
H	-3.444676	-0.732716	-2.028897
H	-2.246879	-1.692548	-2.481181
O	-4.672736	-3.598986	-1.477804

H	-5.611926	-3.305349	-1.543594
H	-4.126916	-2.871649	-1.869986
O	-4.205079	-5.986622	-2.732520
H	-4.374798	-5.130591	-2.270555
H	-3.226250	-6.100108	-2.720510
O	-5.538533	-7.990823	-1.356894
H	-5.009427	-7.292149	-1.806319
H	-5.196375	-8.069174	-0.441377
O	-4.589900	-7.990628	1.283576
H	-4.766347	-8.498868	2.109053
H	-4.852525	-7.076631	1.549151
O	-3.340368	6.899792	-5.032755
H	-2.872636	6.204427	-5.537902
H	-2.877324	6.984444	-4.170233
O	-2.167169	7.095932	-2.508014
H	-2.589033	6.301222	-2.100539
H	-1.197301	6.920814	-2.483408
O	-3.332207	4.829901	-1.528190
H	-4.316930	4.913696	-1.528625
H	-3.061560	4.691287	-0.587416
O	-2.697209	2.589600	-2.907756
H	-3.031647	2.612631	-3.835605
H	-2.920232	3.457656	-2.487069
O	-3.935042	0.737063	-1.328412
H	-3.484027	1.440744	-1.855507
H	-3.584022	0.755232	-0.403816
O	-3.135889	0.539812	1.240368
H	-3.593276	-0.236777	1.648137
H	-2.160419	0.380027	1.267562
O	-4.478658	-1.505230	2.415315
H	-5.461997	-1.428027	2.397785
H	-4.261337	-2.341428	1.928438
O	-3.878216	-3.770415	1.102413
H	-4.189078	-3.729915	0.162976
H	-4.414364	-4.470218	1.556032
O	-5.307515	-5.623350	2.391781
H	-6.263137	-5.378201	2.480729
H	-4.954467	-5.764577	3.305163
O	-4.360619	-6.148234	4.880093
H	-4.658413	-5.364047	5.392888
H	-3.374799	-6.160573	4.918249
O	-2.911095	9.421142	-1.203639
H	-2.598014	8.586790	-1.623087
H	-2.573256	9.419725	-0.283221
O	-1.951485	9.128165	1.418182
H	-2.436164	8.290641	1.617536
H	-1.991240	9.620750	2.272424
O	-3.298172	6.908420	2.210345
H	-4.283784	6.942302	2.276657
H	-3.049736	6.060471	1.765838
O	-2.527320	4.592870	1.058470
H	-2.952344	3.902921	1.627785
H	-1.550581	4.476140	1.156655
O	-3.744399	2.755133	2.600265
H	-3.495156	1.893015	2.179690
H	-3.445044	2.731988	3.541937
O	-3.008724	2.798117	5.222367
H	-3.490303	2.081311	5.692687

H	-3.353870	3.671758	5.543316
O	-4.201626	0.578913	6.389258
H	-4.022816	-0.257371	5.885911
H	-3.892001	0.418744	7.293545
O	-3.761697	-1.735375	5.026429
H	-4.022990	-1.641358	4.075359
H	-2.789312	-1.920320	5.017848
O	-5.295387	-3.770257	5.996638
H	-5.297491	-3.712740	6.964188
H	-4.710901	-3.034568	5.679247
O	-2.325853	7.240772	4.730822
H	-2.617589	7.039971	3.809089
H	-1.370245	7.001721	4.775990
O	-3.890822	5.262474	5.941695
H	-4.794385	5.479609	5.628968
H	-3.308303	5.967919	5.579643
O	-0.977450	-4.153587	-6.429486
H	-1.953607	-3.986552	-6.374558
H	-0.688188	-3.715028	-7.255475
O	-0.378818	-6.554778	-5.042654
H	-0.567983	-5.786545	-5.623532
H	0.604998	-6.661076	-5.016011
O	0.488718	4.623873	-6.496032
H	0.883258	4.654849	-7.381270
H	0.706085	3.724460	-6.129464
O	1.042451	2.190576	-5.452848
H	0.679264	2.185180	-4.532386
H	2.025366	2.132093	-5.360180
O	-0.084766	-0.142291	-6.488597
H	0.371410	0.684292	-6.206280
H	0.116027	-0.828099	-5.807546
O	0.403094	-2.319057	-4.931590
H	-0.097840	-3.034692	-5.391675
H	1.365173	-2.537039	-5.006327
O	-0.567529	-1.958110	-2.445543
H	-0.194723	-2.083708	-3.353644
H	-0.403505	-2.809811	-1.964421
O	-0.146053	-4.278400	-1.181169
H	-0.628302	-4.980724	-1.684641
H	-0.568151	-4.230267	-0.284923
O	-1.451180	-6.205717	-2.554854
H	-1.023307	-6.271985	-3.443896
H	-1.212222	-7.026646	-2.059360
O	-0.815419	-8.542638	-1.283941
H	-1.165669	-8.611612	-0.369908
H	0.164630	-8.644772	-1.228187
O	-1.874400	-8.417759	1.350020
H	-2.830024	-8.179120	1.260729
H	-1.820658	-8.923289	2.186849
O	1.595354	6.610595	-4.855655
H	1.182854	5.956963	-5.462212
H	2.560987	6.404319	-4.825656
O	0.524516	6.505045	-2.357083
H	0.914587	6.482567	-3.265582
H	1.001908	7.222371	-1.871890
O	1.060934	4.090915	-1.255142
H	0.876739	4.984292	-1.640162
H	0.657610	3.435626	-1.877459

O	0.000928	2.227699	-2.922000
H	-0.981249	2.345991	-2.927116
H	0.184293	1.428662	-2.366674
O	0.576904	0.164326	-1.266292
H	0.170782	-0.643575	-1.673336
H	1.554103	0.013910	-1.295957
O	-0.448387	0.279249	1.250113
H	-0.028392	0.307419	0.354900
H	-0.023512	0.966001	1.819718
O	-0.012016	-2.061303	2.496957
H	-0.193679	-1.214629	2.016686
H	-0.476428	-2.781973	1.999875
O	-1.214167	-4.102732	1.257639
H	-2.202940	-4.042772	1.233393
H	-0.962675	-4.927463	1.742507
O	-0.470422	-6.366034	2.506810
H	-0.928609	-7.087009	2.012929
H	0.489208	-6.602044	2.550441
O	-1.618714	-6.414274	4.976467
H	-1.178400	-6.248967	4.108579
H	-1.217381	-5.782948	5.613380
O	1.822815	8.594636	-1.190586
H	1.462278	8.754018	-0.285272
H	2.780794	8.386474	-1.096340
O	0.787866	8.748102	1.368315
H	-0.195154	8.818234	1.308125
H	0.983273	7.821333	1.645466
O	1.456795	6.329717	2.429220
H	1.062690	5.545163	1.972090
H	2.431172	6.198817	2.486842
O	0.166259	4.257938	1.290783
H	0.491690	4.079696	0.374351
H	0.363729	3.466216	1.847551
O	0.673670	2.121691	2.893058
H	0.302815	2.179098	3.806147
H	1.644171	1.949634	2.980048
O	-0.330619	2.301326	5.452456
H	-1.302877	2.476999	5.401783
H	-0.216495	1.428783	5.903360
O	0.064339	-0.210757	6.562925
H	-0.319452	-0.840207	5.906100
H	1.038620	-0.355815	6.530420
O	-1.066160	-2.132104	4.976106
H	-0.690791	-2.103026	4.059982
H	-0.789464	-2.991851	5.373702
O	-0.355440	-4.358568	6.421089
H	-0.457116	-3.810711	7.234136
H	0.621649	-4.488281	6.339838
O	0.386313	6.719106	4.889586
H	0.762580	6.452298	4.016184
H	0.573477	5.979719	5.506294
O	0.871278	4.472083	6.537122
H	0.420362	3.664625	6.172887
H	0.498171	4.609284	7.421700
O	3.969757	-5.230819	-6.065279
H	4.888904	-5.354132	-5.753177
H	3.686350	-4.348314	-5.739944
O	2.299376	-7.053812	-4.861585

H	2.877038	-6.465817	-5.406126
H	2.598033	-6.887456	-3.934780
O	5.370540	4.153014	-6.089314
H	4.789200	3.402063	-5.835772
H	4.989088	4.955495	-5.673897
O	3.776463	1.999464	-5.261426
H	3.961657	1.128292	-5.677984
H	4.036311	1.904372	-4.312280
O	4.175706	-0.528337	-6.396599
H	3.833382	-0.520526	-7.304120
H	5.152741	-0.660623	-6.475540
O	3.091768	-2.759314	-5.078368
H	3.482260	-1.977107	-5.527543
H	3.382475	-2.714420	-4.133796
O	3.838922	-2.685062	-2.460564
H	3.344344	-3.369253	-1.942708
H	4.790681	-2.938666	-2.416864
O	2.528172	-4.573086	-1.061861
H	1.541177	-4.491276	-1.064414
H	2.823703	-4.676444	-0.122858
O	3.263947	-6.805959	-2.338502
H	4.245869	-6.834713	-2.432635
H	3.028552	-5.963689	-1.873920
O	1.902422	-8.956847	-1.441137
H	1.889167	-9.440666	-2.301940
H	2.406023	-8.134458	-1.662043
O	2.717459	-9.298626	1.148840
H	2.375927	-10.107066	1.559793
H	2.402641	-9.307901	0.212619
O	4.305762	6.332038	-4.662692
H	4.574201	7.275383	-4.643670
H	4.530351	6.004149	-3.756410
O	5.109144	5.618074	-2.184355
H	4.612813	4.896603	-1.723146
H	6.047742	5.326908	-2.285782
O	3.714212	3.670472	-0.969005
H	2.737771	3.822262	-1.030760
H	3.965014	3.595961	-0.016393
O	4.525829	1.723703	-2.653439
H	4.270114	2.459967	-2.045254
H	5.498955	1.589177	-2.552362
O	3.256349	-0.273892	-1.343638
H	3.488272	-1.146074	-1.748419
H	3.737365	0.417213	-1.862894
O	3.938161	-0.427247	1.264609
H	3.639061	-0.256473	0.337367
H	3.702891	0.347744	1.828779
O	2.642467	-2.472408	2.443698
H	1.664099	-2.310940	2.440218
H	3.073867	-1.728728	1.953662
O	3.401826	-4.858138	1.478264
H	3.123251	-3.996073	1.878266
H	4.388973	-4.874127	1.505564
O	2.199339	-6.984137	2.667586
H	2.371827	-7.792562	2.136887
H	2.660867	-6.241719	2.204763
O	3.408491	-6.823946	5.206647
H	3.006960	-6.931219	4.316978

H	4.347994	-6.585413	5.067345
O	4.543983	8.106448	-1.296010
H	4.751705	7.173491	-1.553357
H	4.748629	8.627184	-2.102344
O	5.445874	8.150887	1.276559
H	5.141304	8.256770	0.341862
H	5.327307	9.013122	1.702804
O	4.199914	6.065313	2.638220
H	4.655610	6.813257	2.189621
H	4.560432	5.998009	3.550178
O	4.595312	3.574268	1.604104
H	4.507174	4.493730	1.953030
H	5.561397	3.365928	1.585991
O	3.336299	1.624825	2.977821
H	3.813825	2.397912	2.585855
H	3.642759	1.525948	3.909205
O	4.103915	1.450609	5.630824
H	3.666771	0.729111	6.138403
H	3.851322	2.301963	6.053646
O	2.789708	-0.740924	6.690568
H	3.062443	-1.383692	5.987703
H	2.844664	-1.269316	7.513189
O	3.571909	-2.634200	4.941757
H	3.252643	-2.548393	4.007163
H	4.540134	-2.822861	4.912131
O	2.368511	-4.588500	6.370156
H	2.706220	-5.459365	6.041516
H	2.768748	-3.918050	5.763479
O	5.223580	5.552954	5.188104
H	5.368619	6.370016	5.690476
H	4.568979	5.029622	5.711955
O	3.544017	3.963304	6.666929
H	2.575904	4.172246	6.636270
H	3.799402	4.016221	7.601135
O	9.421133	-1.273959	-3.717236
H	9.077692	-1.156658	-2.810308
H	9.229229	-0.438419	-4.186924
O	6.648690	-5.651216	-5.154231
H	7.146410	-4.803892	-5.119930
H	7.123695	-6.213833	-5.784661
O	10.407248	4.479712	0.393184
H	10.160361	5.298804	-0.084342
H	10.013057	4.569310	1.282746
O	8.158070	0.993039	-4.959071
H	7.721116	1.076677	-4.073944
H	8.218229	1.922543	-5.266366
O	6.880576	-0.875298	-6.431656
H	7.272323	-0.900966	-7.318271
H	7.332588	-0.132137	-5.949252
O	7.708773	-3.075206	-4.925723
H	7.283739	-2.418922	-5.520786
H	8.466904	-2.574702	-4.538902
O	6.507919	-3.255116	-2.488408
H	6.918334	-4.023747	-2.034841
H	6.858601	-3.233956	-3.410419
O	7.301455	-5.349840	-0.922998
H	7.983722	-5.979020	-0.588522
H	6.762994	-5.160651	-0.114549

O	5.978912	-6.901230	-2.695377
H	6.230702	-6.563321	-3.581879
H	6.408626	-6.286985	-2.048544
O	6.880023	-9.000711	-1.171627
H	6.552920	-8.393666	-1.875970
H	7.134195	-9.827109	-1.608629
O	5.527273	-9.187610	1.298806
H	4.550990	-9.220131	1.224908
H	5.878991	-9.159674	0.385685
O	7.872269	3.788965	-5.178792
H	6.954410	3.927322	-5.543432
H	8.466809	4.294887	-5.753363
O	7.706575	4.878776	-2.600499
H	7.820637	4.544366	-3.519985
H	7.945062	4.129081	-2.000703
O	8.512735	2.948388	-0.875117
H	7.939870	2.927771	-0.069285
H	9.352652	3.340223	-0.536909
O	7.178518	1.164312	-2.463360
H	7.766038	1.770369	-1.959691
H	7.323924	0.269355	-2.057903
O	7.802761	-1.173152	-1.337020
H	7.269558	-1.920063	-1.701169
H	7.467074	-1.025140	-0.422504
O	6.621885	-0.753340	1.112573
H	5.640418	-0.631291	1.147580
H	7.008384	-0.098060	1.740052
O	7.462807	-2.869037	2.537887
H	8.339401	-2.561011	2.850801
H	7.100234	-2.124018	1.993067
O	6.118283	-5.002598	1.470520
H	6.670376	-4.298398	1.877912
H	6.396496	-5.851518	1.900834
O	6.923900	-7.288955	2.636999
H	6.308767	-7.995293	2.320796
H	6.693269	-7.052568	3.559444
O	6.137566	-6.046833	5.056777
H	6.555380	-6.351902	5.876512
H	6.223462	-5.064499	5.063552
O	9.255711	6.571332	-1.102965
H	8.730466	6.092730	-1.787619
H	9.770234	7.249248	-1.565619
O	8.103243	7.240829	1.378393
H	7.180462	7.564173	1.318759
H	8.408712	7.088431	0.460666
O	8.728585	4.945208	2.668290
H	8.412096	5.829750	2.357935
H	8.442678	4.811195	3.596230
O	7.257278	3.010236	1.514933
H	7.760985	3.762314	1.921067
H	7.567125	2.210203	1.994040
O	7.919044	0.756720	2.944816
H	7.441506	0.900836	3.797422
H	8.656282	0.141956	3.147779
O	6.828941	1.347904	5.366320
H	5.847015	1.369064	5.471833
H	7.131347	2.282268	5.401073
O	7.835191	-1.153849	6.033719

H	7.491881	-0.233731	5.954852
H	8.197157	-1.233022	6.929485
O	6.245043	-3.246632	4.930044
H	6.618295	-3.077602	4.029260
H	6.724156	-2.615985	5.507603
O	8.635645	-7.392545	0.336761
H	8.142185	-8.090617	-0.141542
H	8.239930	-7.358524	1.229378
O	7.591702	4.089347	5.129226
H	6.753823	4.608413	5.172514
H	8.103945	4.360909	5.906893
O	9.447400	-1.403361	3.810557
H	10.407308	-1.494563	3.904208
H	9.065307	-1.389575	4.717843
O	1.933295	9.256291	-3.923207
H	1.725934	9.160437	-2.970999
H	1.638652	8.428883	-4.354714
O	4.623564	9.015841	-3.930557
H	3.658101	9.235901	-3.998697
H	5.112846	9.741816	-4.345034
O	-4.708814	-8.819834	3.923738
H	-4.802956	-7.949484	4.358857
H	-3.768406	-9.054146	4.064399
O	-1.903626	-9.053044	4.124581
H	-1.471573	-9.780499	4.596888
H	-1.684357	-8.230831	4.616706
O	-3.127722	-1.796118	-8.505807
H	-2.175332	-1.978096	-8.642162
H	-3.443253	-2.524458	-7.931839
O	-0.341172	-2.169144	-8.362184
H	0.252413	-2.216970	-9.126497
H	-0.085789	-1.366199	-7.854871
O	-1.888732	9.964195	4.059496
H	-2.179257	9.099445	4.420314
H	-0.911129	9.934409	4.163848
O	0.840153	9.434397	4.139514
H	0.804322	8.548376	4.552462
H	0.952796	9.273769	3.178902
O	-0.209239	-2.418396	8.405673
H	-0.232770	-1.580408	7.900007
H	0.737630	-2.552225	8.619590
O	2.557513	-2.890928	8.507007
H	2.608559	-3.654739	7.884764
H	3.062617	-3.134141	9.296908
O	-1.033742	-9.165543	-4.077214
H	-1.171959	-9.022504	-3.120548
H	-1.021216	-8.281316	-4.493974
O	1.692513	-9.720213	-4.086389
H	0.715141	-9.633454	-4.157245
H	2.037014	-8.912401	-4.518524

Table S5. M06-2X/6-31+G*/PCM optimized Cartesian coordinates of the $(H_2O)_{212}$ hexagonal cluster.

O	-1.477175	9.602503	-5.898352

H	-1.553453	10.567209	-5.860402
H	-0.853611	9.382908	-6.638010
O	2.162463	7.357794	-5.979357
H	3.001740	7.590134	-5.523847
H	2.176262	6.379526	-6.122467
O	0.228867	8.538262	-7.696539
H	-0.336676	7.806941	-8.019803
H	0.986652	8.135019	-7.226569
O	-1.886776	6.848981	-8.483693
H	-2.269154	7.275196	-9.266505
H	-2.490324	7.073005	-7.726402
O	-3.330936	7.535815	-6.299571
H	-2.926787	6.977912	-5.586051
H	-2.942530	8.423502	-6.147360
O	-5.931529	6.426199	-6.100581
H	-5.110416	6.958334	-6.111701
H	-6.491292	6.754963	-5.370393
O	8.449112	8.823079	-1.948071
H	9.245756	9.364825	-2.054891
H	8.756780	7.885137	-1.941014
O	6.411814	9.646466	-3.751980
H	5.912897	10.175098	-3.096346
H	7.154629	9.266999	-3.239786
O	4.473486	7.775115	-4.377572
H	5.217834	8.422041	-4.381060
H	4.083815	7.916192	-3.479442
O	3.515060	8.426122	-1.905863
H	3.982129	8.002036	-1.143925
H	2.590215	8.083018	-1.880949
O	1.081457	7.223577	-1.944067
H	0.635944	7.504149	-2.778272
H	1.303703	6.263925	-2.035893
O	0.042319	7.875833	-4.350600
H	0.804190	7.710821	-4.960096
H	-0.453840	8.644530	-4.706370
O	-2.126088	6.234878	-4.272749
H	-1.241073	6.665396	-4.292081
H	-1.993426	5.254618	-4.320515
O	-3.762161	7.110105	-2.239691
H	-3.141155	6.787204	-2.930255
H	-3.318305	6.951179	-1.375922
O	-6.011718	5.675979	-2.245426
H	-5.162597	6.198816	-2.272641
H	-6.590829	6.036044	-2.957692
O	-7.754900	6.888963	-3.972544
H	-8.056380	7.563905	-3.327884
H	-8.532153	6.297063	-4.095764
O	-10.030113	5.314241	-4.050582
H	-10.613181	5.538927	-4.791749
H	-9.841233	4.332235	-4.133126
O	-10.860349	6.415315	-1.681514
H	-11.791876	6.678132	-1.731009
H	-10.660786	5.931099	-2.519885
O	7.205332	8.648731	2.865847
H	6.924580	9.059330	2.025566
H	6.413404	8.517596	3.423346
O	6.767195	9.534442	0.156123
H	7.459883	9.286502	-0.495960

H	6.158724	10.128130	-0.334730
O	4.863762	7.602428	0.277402
H	5.677511	8.156438	0.233909
H	5.137784	6.651179	0.311482
O	3.459130	8.470157	2.448335
H	4.044403	8.214030	3.203447
H	3.953143	8.212439	1.633054
O	1.321595	6.857922	2.356380
H	0.810952	7.210409	1.590143
H	2.152917	7.397360	2.402218
O	-0.158877	7.888774	0.308028
H	0.291104	7.685810	-0.556139
H	-0.236897	8.853091	0.366184
O	-2.569905	6.584568	0.186039
H	-1.701780	7.044258	0.245320
H	-2.377927	5.615144	0.186033
O	-4.056534	7.158122	2.403628
H	-3.515269	7.043940	1.585928
H	-4.833661	6.558116	2.289100
O	-6.301282	5.671463	2.235587
H	-6.763358	6.050747	1.453705
H	-6.781886	5.989708	3.042620
O	-7.446748	6.482408	-0.112647
H	-7.840746	7.300252	-0.482634
H	-6.883910	6.125087	-0.846793
O	-9.724291	5.114309	0.530804
H	-10.328004	5.514634	-0.130231
H	-8.846098	5.498923	0.294739
O	1.568976	9.856568	4.051639
H	2.119584	9.519676	3.317781
H	0.772475	9.290236	4.078201
O	4.921909	10.716310	-1.600114
H	4.508775	11.589764	-1.533034
H	4.196015	10.053675	-1.686178
O	5.034159	7.869694	4.582623
H	4.387158	8.136466	5.278588
H	5.135458	6.890313	4.653787
O	-8.819177	8.256304	-1.753667
H	-9.021396	9.200722	-1.678946
H	-9.675610	7.771908	-1.699707
O	0.714478	7.096770	6.848817
H	0.382950	7.302204	5.936891
H	-0.088728	7.120652	7.410399
O	-0.231111	7.707349	4.393837
H	0.308963	7.266980	3.694757
H	-1.104782	7.245660	4.387598
O	-2.541288	6.270279	4.435418
H	-3.096799	6.502270	3.647488
H	-2.381481	5.294528	4.420480
O	-4.028475	7.177342	6.484168
H	-3.500276	6.738688	5.769325
H	-4.506254	7.877432	5.986071
O	-6.408625	5.701345	6.675737
H	-5.524813	6.129391	6.679220
H	-6.250430	4.734591	6.711350
O	-7.586459	6.630813	4.404239
H	-8.459643	6.238257	4.158452
H	-7.267353	6.192489	5.231947

O	-9.805009	5.363931	3.387753
H	-9.816274	5.350378	2.409003
H	-9.755216	4.418931	3.666246
O	2.970971	8.762513	6.195586
H	2.323142	8.159908	6.610706
H	2.462379	9.234933	5.497464
O	-1.839906	6.981216	8.160632
H	-1.918586	7.455412	9.002151
H	-2.634347	7.232368	7.632645
O	4.773335	3.978033	-6.619067
H	5.168418	4.372403	-7.411431
H	4.943233	3.004418	-6.675520
O	2.094873	4.657162	-6.431624
H	3.027854	4.351326	-6.479200
H	1.676487	4.175052	-5.677215
O	0.962697	3.516480	-8.718171
H	1.329623	3.947702	-7.916038
H	0.005702	3.723270	-8.726931
O	-1.785911	4.134910	-8.772861
H	-1.851543	5.119330	-8.705498
H	-2.171341	3.896790	-9.629821
O	-3.081230	2.853099	-6.677825
H	-2.660241	3.324611	-7.431081
H	-4.025025	3.161901	-6.628777
O	-5.641789	3.673659	-6.473093
H	-5.749531	4.627695	-6.260946
H	-6.099115	3.159751	-5.770769
O	9.234353	6.185829	-1.927103
H	10.199060	6.089286	-1.945818
H	8.896455	5.740573	-2.744884
O	8.215332	4.996282	-4.149845
H	8.592354	5.381539	-4.955713
H	7.235121	5.148485	-4.206766
O	5.537258	5.268250	-4.293241
H	5.167177	6.186348	-4.349134
H	5.259600	4.802011	-5.114297
O	4.467140	4.044802	-2.070988
H	4.872317	4.475072	-2.861155
H	4.675653	3.080282	-2.139294
O	1.774089	4.573111	-2.108910
H	2.746682	4.400118	-2.086953
H	1.439436	4.114624	-2.918945
O	0.878519	3.324525	-4.370774
H	-0.107517	3.390243	-4.404227
H	1.081684	2.357148	-4.384684
O	-1.824880	3.568542	-4.387022
H	-2.303549	3.265023	-5.198368
H	-2.287113	3.192339	-3.597601
O	-3.045904	2.623454	-2.149026
H	-2.680037	3.060583	-1.340997
H	-2.834992	1.658864	-2.086734
O	-5.745701	2.989829	-2.165800
H	-5.839993	3.974457	-2.199897
H	-4.771639	2.819341	-2.154156
O	-6.867003	2.132693	-4.498359
H	-6.444108	2.408372	-3.649073
H	-6.704838	1.160749	-4.573368
O	-9.545414	2.729883	-4.304925

H	-8.588968	2.498660	-4.371516
H	-9.908740	2.217624	-3.550954
O	-10.658451	1.360414	-2.144346
H	-11.620412	1.478068	-2.149551
H	-10.333982	1.784814	-1.309572
O	8.830296	6.394167	2.639942
H	8.233928	7.175054	2.669344
H	8.624355	5.911394	1.810165
O	8.241143	4.911236	0.352453
H	8.580997	5.353911	-0.455825
H	7.255267	4.930468	0.286230
O	5.522493	4.991922	0.266291
H	5.126032	4.610269	-0.554171
H	5.106515	4.544153	1.045252
O	4.438689	3.884001	2.490641
H	3.453247	3.971393	2.454953
H	4.634652	2.914214	2.506328
O	1.750063	4.211561	2.366712
H	1.596800	5.189914	2.378243
H	1.308908	3.853041	3.176900
O	0.599921	3.386870	0.060599
H	1.046152	3.831446	-0.700913
H	1.033647	3.711510	0.889421
O	-2.080268	3.881826	0.080426
H	-1.105412	3.717066	0.099438
H	-2.458904	3.412945	0.864111
O	-3.176111	2.587937	2.222273
H	-4.157985	2.708923	2.216849
H	-3.004019	1.614507	2.192780
O	-5.868924	2.983970	2.229824
H	-6.058913	3.953549	2.201594
H	-6.277073	2.592660	1.417978
O	-6.963880	1.879801	-0.001737
H	-6.567482	2.308407	-0.801070
H	-7.931184	2.076434	-0.002298
O	-9.644812	2.417225	0.131198
H	-9.715410	3.391773	0.288185
H	-10.062486	1.967509	0.901425
O	-10.849709	1.110354	2.245424
H	-11.814682	1.196782	2.219612
H	-10.660316	0.138675	2.259454
O	8.127605	4.801710	4.750717
H	8.439780	5.345593	3.986895
H	7.150462	4.915827	4.764667
O	5.390571	5.153061	4.713410
H	5.027978	4.678003	3.926324
H	4.979138	4.743250	5.508224
O	4.253687	4.012694	6.955240
H	3.283069	4.228336	6.937165
H	4.602453	4.394887	7.774636
O	1.618652	4.511122	6.824666
H	1.317039	5.452854	6.814258
H	1.229201	4.075822	6.027233
O	0.590306	3.249243	4.631430
H	-0.392862	3.346141	4.580420
H	0.772975	2.277700	4.670732
O	-2.100894	3.593187	4.496365
H	-2.514039	3.187137	3.693997

H	-2.556458	3.218071	5.289232
O	-3.239065	2.646522	6.789935
H	-4.213410	2.784830	6.807718
H	-2.844864	3.217644	7.502391
O	-5.986638	2.941418	6.727917
H	-6.368412	2.549543	5.896719
H	-6.440869	2.509858	7.467015
O	-7.017136	1.929189	4.463347
H	-6.575904	2.300269	3.659363
H	-6.870097	0.952052	4.438018
O	-9.667228	2.739024	4.224416
H	-10.087427	2.132844	3.581374
H	-8.741200	2.426005	4.322814
O	0.485500	3.361409	9.088231
H	0.935610	3.724205	8.294615
H	0.657154	2.390642	9.083781
O	-2.087629	4.178654	8.688033
H	-2.003718	5.132074	8.481737
H	-1.169683	3.853407	8.860957
O	5.171957	1.291931	-6.671546
H	5.645851	0.985300	-7.459815
H	4.302950	0.801382	-6.656440
O	2.849421	-0.019589	-6.591967
H	2.322586	0.174496	-5.777676
H	3.014489	-0.992987	-6.606866
O	1.373471	0.792238	-8.821953
H	1.241900	1.766828	-8.753998
H	1.895323	0.526164	-8.035075
O	-1.029833	-0.502125	-8.817341
H	-0.150602	-0.049665	-8.820698
H	-0.848929	-1.463501	-8.843963
O	-2.497486	0.158445	-6.651888
H	-2.710154	1.120209	-6.694188
H	-1.955736	-0.055050	-7.456832
O	-4.922382	-1.160599	-6.778399
H	-4.048061	-0.709115	-6.745935
H	-4.732829	-2.121535	-6.807131
O	10.253857	1.440814	-1.921467
H	9.735221	1.730345	-2.707702
H	9.744675	1.735256	-1.134735
O	8.835766	2.223077	-4.145657
H	8.577115	3.166616	-4.135386
H	8.006328	1.702776	-4.225257
O	6.531133	0.676679	-4.337506
H	6.058337	0.884891	-5.174189
H	6.755944	-0.284974	-4.380616
O	5.061933	1.378691	-2.126318
H	5.561263	1.094103	-2.929707
H	4.211689	0.874141	-2.119651
O	2.666027	0.069942	-2.081409
H	2.123974	0.323615	-1.293246
H	2.794472	-0.909088	-2.029651
O	1.359849	0.628529	-4.400739
H	1.804138	0.400370	-3.546559
H	0.479959	0.178541	-4.396963
O	-1.108071	-0.579148	-4.423141
H	-1.609920	-0.310259	-5.233353
H	-0.991664	-1.559137	-4.466819

O	-2.551349	-0.041833	-2.175259
H	-2.047366	-0.242071	-3.002893
H	-3.423619	-0.501971	-2.249634
O	-4.966027	-1.299345	-2.338182
H	-5.477521	-1.025151	-3.141153
H	-5.532435	-1.101786	-1.552810
O	-6.356698	-0.562698	-4.564138
H	-5.839640	-0.797797	-5.377969
H	-7.183983	-1.110225	-4.579626
O	-8.623424	-2.044137	-4.522015
H	-9.148006	-1.829630	-3.713785
H	-8.439893	-3.004852	-4.491612
O	-10.089700	-1.402542	-2.296772
H	-10.254135	-0.440177	-2.216790
H	-9.639303	-1.679238	-1.468796
O	10.138159	1.437288	2.547285
H	9.649507	1.685310	3.361579
H	10.263443	0.466128	2.578882
O	8.803602	2.220537	0.319862
H	8.659958	3.195698	0.364307
H	9.285346	1.955340	1.144609
O	6.458978	0.858069	0.177544
H	5.982199	1.079886	-0.658555
H	7.299305	1.382338	0.195253
O	4.987587	1.224760	2.447537
H	5.536360	1.077128	1.639177
H	5.540829	0.984074	3.230494
O	2.511786	0.053626	2.359070
H	3.403946	0.477318	2.368649
H	2.050672	0.351307	1.536289
O	1.171423	0.740907	0.088005
H	0.934399	1.701492	0.054168
H	0.320003	0.237552	0.065933
O	-1.136880	-0.698874	0.046917
H	-1.657647	-0.485434	-0.766974
H	-0.896434	-1.654790	-0.020806
O	-2.647445	-0.080263	2.234016
H	-2.072836	-0.341647	1.473246
H	-2.159140	-0.297633	3.065005
O	-5.079266	-1.312170	2.197661
H	-4.209661	-0.842521	2.177687
H	-5.553483	-1.093372	1.357116
O	-6.449714	-0.789530	-0.094574
H	-6.692269	0.169260	-0.074627
H	-7.295140	-1.300269	-0.054970
O	-8.778782	-2.223562	0.016123
H	-9.312785	-2.017003	0.816752
H	-8.604607	-3.196220	0.028650
O	-10.291222	-1.560657	2.240300
H	-11.113939	-2.073594	2.251798
H	-9.779824	-1.830193	3.064084
O	8.703805	2.219557	4.801654
H	8.504635	3.201299	4.762696
H	9.206063	2.079172	5.618325
O	6.438432	0.636178	4.692947
H	7.237415	1.209758	4.751739
H	5.860976	0.875780	5.465494
O	4.866830	1.264979	6.799045

H	4.625284	2.213364	6.852859
H	4.021763	0.761966	6.839605
O	2.479405	-0.131984	6.901487
H	1.983277	0.079735	6.074625
H	2.622354	-1.106431	6.903330
O	1.105648	0.575787	4.646763
H	1.592599	0.379620	3.809926
H	0.232353	0.115805	4.590875
O	-1.330336	-0.651105	4.565762
H	-1.841081	-0.414775	5.376298
H	-1.160350	-1.624708	4.617037
O	-2.742400	-0.033659	6.849577
H	-2.933661	0.938303	6.865317
H	-3.607884	-0.499722	6.801023
O	-5.116146	-1.477466	6.703655
H	-5.677978	-1.306992	7.475444
H	-4.955303	-2.453270	6.695923
O	-6.557398	-0.769213	4.421662
H	-6.006562	-0.966109	3.624533
H	-6.021085	-1.022279	5.205278
O	-8.913144	-2.234134	4.376113
H	-8.067225	-1.733050	4.395248
H	-8.672007	-3.193704	4.355828
O	1.001999	0.655682	9.111778
H	1.524944	0.371979	8.328888
H	0.175144	0.133046	9.087501
O	-1.411370	-0.807261	9.064044
H	-1.924135	-0.516972	8.263600
H	-1.944325	-0.551074	9.832380
O	5.888088	-3.498761	-6.415717
H	6.350620	-3.027937	-5.686639
H	6.054521	-4.456297	-6.299083
O	3.304784	-2.721062	-6.642046
H	4.249505	-3.019702	-6.581818
H	2.911352	-3.158208	-7.429141
O	2.086822	-3.928617	-8.844877
H	2.528873	-3.657973	-9.664648
H	2.152311	-4.916501	-8.810612
O	-0.579223	-3.302373	-8.894706
H	-0.951792	-3.663397	-9.713668
H	0.384148	-3.519188	-8.915421
O	-1.784357	-4.417838	-6.668002
H	-1.327285	-4.053923	-7.460432
H	-1.372185	-3.985897	-5.877805
O	-4.421086	-3.916894	-6.789566
H	-3.442436	-4.091737	-6.784220
H	-4.772009	-4.355887	-7.579338
O	10.730673	-1.245692	-1.921732
H	10.530582	-0.276899	-1.899944
H	10.315041	-1.641007	-1.124626
O	9.783786	-2.487557	-4.059697
H	10.286872	-2.183264	-4.830073
H	10.145755	-1.984347	-3.272300
O	7.073730	-2.011093	-4.381559
H	8.037382	-2.181655	-4.266509
H	6.637830	-2.291955	-3.540812
O	5.796382	-2.771613	-2.104216
H	4.813814	-2.659630	-2.093418

H	6.150779	-2.401887	-1.257257
O	3.092898	-2.607583	-2.094202
H	2.697348	-2.980639	-2.920663
H	2.736362	-3.123029	-1.331123
O	2.030847	-3.596246	-4.397035
H	2.476807	-3.234788	-5.201973
H	2.227459	-4.566010	-4.403495
O	-0.675180	-3.287948	-4.457194
H	0.307020	-3.406211	-4.444419
H	-1.023142	-3.747635	-3.655140
O	-1.700926	-4.525183	-2.233369
H	-2.674283	-4.350717	-2.237401
H	-1.572652	-5.501208	-2.171419
O	-4.373730	-3.964455	-2.309194
H	-4.580542	-2.997060	-2.328347
H	-4.806118	-4.327446	-1.495946
O	-5.382991	-5.154869	-4.534190
H	-5.048948	-4.655453	-5.316353
H	-5.013612	-4.706418	-3.731564
O	-8.061189	-4.825571	-4.530617
H	-8.393078	-5.206776	-5.357995
H	-7.077225	-4.969622	-4.536419
O	-9.178015	-6.039602	-2.370667
H	-10.141643	-5.953404	-2.432946
H	-8.805032	-5.587998	-3.169655
O	10.595855	-1.340882	2.539119
H	11.556467	-1.461012	2.482307
H	10.221943	-1.705502	1.689766
O	9.530809	-2.387645	0.320019
H	8.559837	-2.200900	0.284827
H	9.626331	-3.365485	0.411969
O	6.866548	-1.822593	0.201214
H	6.737474	-0.841256	0.223061
H	6.396895	-2.193114	0.988288
O	5.623242	-2.948157	2.350287
H	4.649405	-2.772713	2.386222
H	6.012548	-2.617247	3.194643
O	2.942467	-2.623740	2.338962
H	2.727588	-1.657529	2.343218
H	2.541039	-3.018938	3.151486
O	2.133093	-3.934934	0.096145
H	2.446063	-3.479580	0.916616
H	2.394470	-4.881509	0.167519
O	-0.532802	-3.349387	-0.063907
H	-0.943040	-3.788781	-0.848874
H	0.420956	-3.604015	-0.045962
O	-1.792252	-4.164507	2.191737
H	-1.313899	-3.782343	1.415763
H	-1.398109	-3.799035	3.021478
O	-4.496519	-3.960325	2.179481
H	-4.724943	-2.996997	2.194126
H	-3.509063	-4.007705	2.170539
O	-5.498860	-5.060339	-0.094513
H	-5.121539	-4.669129	0.732465
H	-5.222037	-6.010239	-0.111187
O	-8.210930	-4.906985	-0.017212
H	-8.553233	-5.293478	-0.853201
H	-7.224549	-4.967957	-0.068178

O	-8.939698	-6.434650	2.194521
H	-8.673332	-5.946692	1.384526
H	-8.348020	-7.210457	2.269940
O	9.461145	-2.949110	4.418192
H	9.925644	-2.342978	3.786472
H	9.911067	-2.844057	5.271003
O	6.827008	-2.058522	4.649812
H	6.783390	-1.069246	4.670573
H	7.773810	-2.317665	4.583248
O	5.701053	-3.119651	6.864165
H	6.123693	-2.702369	6.068626
H	6.125275	-2.719292	7.638010
O	2.959820	-2.849516	6.863166
H	3.936605	-2.971520	6.900610
H	2.567312	-3.383156	7.590862
O	1.860645	-3.784655	4.551651
H	2.291521	-3.463784	5.381297
H	1.972086	-4.766964	4.534497
O	-0.803540	-3.309758	4.572081
H	0.171212	-3.479550	4.605784
H	-1.205088	-3.793837	5.333322
O	-1.919618	-4.600754	6.718612
H	-2.895813	-4.469366	6.710997
H	-1.592117	-4.178335	7.544716
O	-4.647614	-4.183375	6.630730
H	-4.979092	-4.599251	5.786247
H	-5.103457	-4.641338	7.353723
O	-5.515239	-5.251549	4.342907
H	-5.134255	-4.781476	3.559821
H	-5.318877	-6.212741	4.219526
O	-8.275400	-4.883707	4.370808
H	-8.579257	-5.405411	3.591688
H	-7.300884	-5.001776	4.385528
O	1.677317	-4.400236	8.771937
H	2.111931	-4.385170	9.638450
H	0.775103	-4.026941	8.915454
O	-0.900281	-3.481107	9.038088
H	-1.075174	-2.506361	9.069921
H	-1.335726	-3.859370	9.817559
O	6.355369	-6.321398	-6.294000
H	6.928574	-6.523573	-7.049166
H	6.881243	-6.547491	-5.487536
O	3.780035	-7.380149	-6.502945
H	4.715261	-7.077085	-6.476682
H	3.385029	-7.055977	-7.342449
O	2.251215	-6.641339	-8.701651
H	2.551952	-7.028158	-9.538405
H	1.418677	-7.135373	-8.452943
O	0.224938	-8.178044	-7.904263
H	0.724827	-8.695880	-7.230672
H	-0.542927	-7.797644	-7.425661
O	-1.772555	-7.147932	-6.178145
H	-1.777060	-6.184079	-6.379522
H	-1.095774	-7.265860	-5.467664
O	1.783151	-9.336751	-5.926671
H	2.599423	-8.805447	-6.008978
H	1.278611	-8.945615	-5.184047
O	10.708208	-6.544005	-1.355161

H	10.020446	-7.231412	-1.466571
H	10.623715	-5.983931	-2.162311
O	10.222022	-5.242480	-3.740350
H	10.080924	-4.275039	-3.816994
H	9.355890	-5.659937	-3.934429
O	7.774602	-6.591439	-3.976576
H	7.989671	-7.378066	-3.434995
H	7.209225	-6.056404	-3.364251
O	6.211653	-5.419544	-2.125659
H	6.055159	-4.439591	-2.121286
H	6.660401	-5.647770	-1.271291
O	4.082840	-7.023002	-2.239521
H	4.426351	-7.918951	-2.378740
H	4.875277	-6.418682	-2.223129
O	2.520188	-6.253198	-4.380423
H	3.001054	-6.586062	-5.178126
H	3.056893	-6.535670	-3.604318
O	0.190564	-7.673223	-4.339858
H	0.948237	-7.042715	-4.330556
H	-0.243786	-7.582706	-3.458148
O	-1.141265	-7.218845	-2.013623
H	-1.916173	-7.825847	-2.035144
H	-0.611944	-7.446586	-1.215725
O	-3.424818	-8.616427	-2.391138
H	-3.725223	-8.275384	-3.269209
H	-3.853758	-9.495081	-2.254136
O	-4.205277	-7.633838	-4.816217
H	-4.599644	-6.736196	-4.707320
H	-3.389266	-7.514846	-5.356116
O	-6.290025	-9.471751	-4.105284
H	-5.645002	-8.832654	-4.466808
H	-6.952659	-8.964857	-3.593880
O	-8.272452	-8.634260	-2.275896
H	-8.635811	-7.717383	-2.308920
H	-9.016922	-9.220387	-2.481781
O	8.380553	-8.143408	-1.698769
H	8.340958	-9.095625	-1.525807
H	7.894537	-7.699748	-0.966805
O	9.746673	-5.087950	0.705239
H	10.278166	-5.626828	0.065661
H	9.946082	-5.361177	1.626256
O	7.400443	-6.326465	0.096060
H	8.225327	-5.831224	0.322531
H	6.793519	-6.167767	0.859746
O	5.914926	-5.637664	2.251382
H	5.756856	-4.661122	2.280342
H	5.052724	-6.106541	2.345547
O	3.845330	-7.356511	2.554029
H	4.375591	-8.014737	3.052656
H	3.174189	-7.022368	3.201612
O	2.784906	-6.631788	0.132802
H	3.297247	-6.837865	-0.684489
H	3.281135	-6.989108	0.902476
O	0.383740	-7.798055	0.189221
H	1.297466	-7.408769	0.140145
H	0.493129	-8.746181	0.357976
O	-1.193293	-6.773502	2.174343
H	-1.381374	-5.800988	2.140676

H	-0.585900	-7.010750	1.437847
O	-3.299797	-8.427024	1.975133
H	-3.799949	-8.237096	1.146458
H	-2.576799	-7.753496	2.004387
O	-4.744656	-7.657835	-0.221106
H	-5.527127	-8.262878	-0.212211
H	-4.256622	-7.869869	-1.052783
O	-6.793802	-9.420948	-0.071670
H	-7.492257	-9.106044	-0.685795
H	-7.089236	-9.266230	0.851341
O	-7.307591	-8.729108	2.617363
H	-6.526116	-8.510935	3.180458
H	-7.831763	-9.365277	3.127239
O	9.778406	-5.554606	3.483693
H	9.694153	-4.637832	3.837636
H	10.598104	-5.907501	3.862396
O	7.294956	-6.627849	4.349401
H	6.822320	-6.187637	3.599229
H	8.242101	-6.439454	4.183478
O	6.023685	-5.899621	6.720608
H	5.922496	-4.926698	6.778640
H	6.547883	-6.074939	5.908604
O	3.827514	-7.461379	6.399522
H	4.278877	-8.127218	5.840639
H	4.547825	-6.809904	6.599914
O	2.170918	-6.485719	4.479525
H	2.721952	-6.784119	5.244384
H	1.353088	-7.044609	4.479287
O	-0.130686	-7.933697	4.349221
H	-0.583488	-7.661358	5.184411
H	-0.545671	-7.409620	3.620853
O	-1.164432	-7.260029	6.778169
H	-1.450203	-6.317082	6.763490
H	-0.289890	-7.266221	7.233878
O	-3.313925	-8.979990	5.899846
H	-2.656894	-8.389393	6.318224
H	-2.807502	-9.487783	5.225414
O	-5.031596	-7.926755	3.992710
H	-4.379092	-8.059685	3.257085
H	-4.566320	-8.273141	4.791035
O	-5.091318	-10.746847	-1.967767
H	-5.598483	-10.443521	-2.755617
H	-5.634553	-10.484165	-1.199651
O	1.385946	-7.084706	7.892135
H	1.529856	-6.159910	8.181780
H	2.202253	-7.348802	7.424545
O	-1.878327	-10.076088	3.783840
H	-1.120305	-9.465206	3.903332
H	-2.385709	-9.710734	3.031501
O	-5.445544	8.529196	4.482149
H	-4.913291	8.263035	3.706378
H	-6.281433	8.028334	4.403238
O	5.458607	-8.685430	4.421748
H	6.271654	-8.129101	4.429173
H	5.753791	-9.607487	4.383335

Table S6. M06-2X/6-31+G*/PCM optimized Cartesian coordinates of the $(H_2O)_{254}$ hexagonal cluster.

O	9.165665	8.837611	-2.657257
H	9.679540	9.483070	-3.167018
H	8.266740	9.234007	-2.555733
O	11.550910	6.917430	1.769266
H	12.518664	6.948355	1.730131
H	11.220071	7.448497	1.011784
O	8.796792	-9.031478	3.968320
H	9.099038	-9.295725	3.075329
H	7.817627	-9.066499	3.964965
O	10.056426	-9.431251	1.465238
H	10.639835	-8.643875	1.572028
H	10.641967	-10.203683	1.470392
O	8.762106	-0.875486	8.765589
H	9.145474	-1.111629	9.623868
H	9.106691	0.031333	8.548105
O	9.793605	-2.234409	6.590722
H	10.534103	-1.628102	6.375221
H	9.404629	-1.863113	7.415414
O	9.059513	-4.952053	6.181514
H	9.375884	-4.032270	6.287853
H	8.081971	-4.927942	6.238191
O	9.707797	-6.478924	4.011369
H	9.564479	-5.948476	4.833229
H	9.339867	-7.386741	4.152403
O	8.323351	-5.152426	2.111302
H	8.701951	-5.678051	2.858169
H	7.336136	-5.197624	2.162146
O	9.469871	-6.194641	-0.057094
H	8.957318	-5.807587	0.701462
H	10.346250	-6.381897	0.334182
O	8.574678	-8.654500	-0.788739
H	9.079971	-9.181461	-0.134169
H	8.807451	-7.725942	-0.541743
O	11.324187	-7.007867	1.874607
H	12.285037	-6.943911	1.981553
H	10.920726	-6.702823	2.721593
O	8.852408	-8.644654	-3.618175
H	9.289060	-7.817980	-3.907217
H	8.863294	-8.651432	-2.638433
O	9.953267	1.417093	8.003117
H	10.582221	1.030768	7.358592
H	9.458015	2.104788	7.511624
O	8.534782	3.068150	6.198255
H	8.656411	3.955869	5.798706
H	8.798206	2.411490	5.508882
O	9.444485	1.136366	4.483825
H	8.933961	0.287994	4.455666
H	9.334384	1.540862	3.590462
O	8.367225	-1.332676	4.430393
H	8.727891	-1.739976	5.251088
H	7.378947	-1.380702	4.469645
O	9.422700	-2.641618	2.240257
H	9.014362	-3.536657	2.229095
H	9.038800	-2.179756	3.018922

O	8.635789	-1.464885	-0.032726
H	8.938001	-1.891187	0.815072
H	8.982548	-0.544085	-0.018110
O	9.598367	-2.554186	-2.345422
H	9.339703	-2.171805	-1.471685
H	9.163772	-1.989514	-3.029526
O	8.455025	-4.984448	-2.295096
H	8.900517	-5.410182	-1.528060
H	8.812069	-4.058804	-2.324558
O	9.635058	-6.053329	-4.498545
H	9.099121	-5.691667	-3.751938
H	10.400421	-5.437435	-4.516878
O	8.599614	-4.883796	-6.838801
H	8.882726	-5.369146	-6.033811
H	7.613442	-4.938405	-6.858596
O	11.370318	0.019881	6.005546
H	10.886309	0.467697	5.271006
H	12.316562	0.085177	5.809384
O	8.671244	5.522861	4.700054
H	8.900425	5.232759	3.786934
H	9.172507	6.359403	4.779280
O	9.608582	4.941991	2.174262
H	10.506490	5.325734	2.137234
H	9.161973	5.335966	1.382666
O	8.827925	2.345217	2.131656
H	9.172538	3.269578	2.144477
H	7.839158	2.406003	2.162060
O	9.537717	1.119783	-0.113386
H	9.330774	1.591448	0.739329
H	10.490436	1.212598	-0.264535
O	8.258155	2.342620	-2.184679
H	8.583387	1.864161	-1.388037
H	7.266145	2.380967	-2.166659
O	9.657077	1.397480	-4.257466
H	10.481361	1.922135	-4.178247
H	9.076709	1.726133	-3.525237
O	8.610319	-1.143584	-4.432645
H	9.083643	-0.282574	-4.428085
H	8.980280	-1.657809	-5.195648
O	9.744303	-2.454489	-6.493433
H	9.244297	-3.276497	-6.733957
H	9.534302	-1.750956	-7.149923
O	8.904921	-0.274630	-8.010935
H	8.894060	0.622437	-7.622892
H	7.980621	-0.480697	-8.265467
O	10.142537	7.858921	3.964696
H	10.771776	7.508840	3.292528
H	10.686270	8.306597	4.631411
O	9.052750	9.719623	2.208276
H	9.359752	9.104165	2.914957
H	9.484735	10.567987	2.392205
O	10.031985	8.406848	-0.020794
H	9.618899	9.023915	0.623272
H	9.835875	8.722371	-0.930574
O	8.674591	6.060686	-0.089501
H	9.109466	6.947659	-0.069928
H	9.094314	5.567512	-0.830417
O	9.612226	4.642131	-2.254196

H	9.049753	3.825634	-2.222348
H	10.446458	4.353675	-2.681024
O	8.709052	6.417001	-4.099662
H	8.956996	5.775383	-3.388674
H	8.928015	7.299362	-3.728561
O	9.955932	4.835496	-6.021879
H	9.492375	5.517434	-5.481315
H	10.425185	5.317970	-6.719574
O	8.736990	2.295729	-6.650572
H	9.108118	3.201036	-6.672362
H	9.008533	1.952865	-5.764110
O	11.425486	3.542078	-4.070195
H	12.391952	3.583589	-4.121288
H	11.073856	4.029660	-4.851008
O	6.615001	9.871676	-2.343420
H	6.628718	10.840898	-2.313266
H	6.092879	9.626811	-3.149282
O	5.234417	8.960393	-4.495948
H	5.550008	9.339487	-5.330664
H	5.501578	7.999935	-4.510110
O	5.986157	-9.072181	4.123776
H	5.740404	-9.638091	4.871727
H	5.627203	-9.516722	3.316079
O	5.058394	-10.263608	1.852881
H	5.360769	-11.182982	1.795498
H	5.411539	-9.807058	1.043236
O	5.983605	-1.250181	8.860833
H	6.953745	-1.154928	8.767737
H	5.669077	-1.763694	8.085612
O	4.986438	-2.688261	6.687557
H	5.358952	-3.597109	6.634133
H	4.000772	-2.765361	6.713927
O	6.228922	-5.160792	6.450377
H	6.117113	-5.680122	7.261456
H	5.774103	-5.677213	5.737518
O	4.975327	-6.512960	4.444645
H	5.298630	-7.436942	4.337201
H	3.986092	-6.553201	4.458302
O	5.627449	-5.216279	2.135827
H	5.345627	-5.647812	2.978285
H	5.324873	-4.274147	2.166426
O	4.827396	-6.453633	-0.153629
H	5.132305	-6.043474	0.692637
H	5.207660	-7.363437	-0.194217
O	5.896076	-8.973403	-0.355286
H	6.870200	-8.882865	-0.505320
H	5.533028	-9.450496	-1.139821
O	4.883603	-10.318282	-2.518195
H	5.138471	-11.253006	-2.509505
H	3.882353	-10.300386	-2.521274
O	6.213286	-8.795234	-4.470942
H	7.147697	-8.726890	-4.165700
H	5.749529	-9.359012	-3.817351
O	5.056870	1.332222	8.869154
H	5.351876	0.391370	8.865291
H	4.071158	1.309681	8.880190
O	5.843788	2.603412	6.635476
H	6.818790	2.733389	6.585525

H	5.626561	2.120226	7.477540
O	5.010721	1.282314	4.426408
H	5.328022	1.727043	5.253448
H	5.273787	0.331612	4.482578
O	5.678478	-1.355796	4.440447
H	5.371320	-1.867902	5.229831
H	5.359940	-1.825048	3.630806
O	4.871470	-2.610495	2.159369
H	5.226608	-2.154031	1.357411
H	3.883791	-2.572158	2.107288
O	5.899015	-1.348484	-0.043827
H	6.887031	-1.397400	-0.053768
H	5.582486	-1.775294	-0.878298
O	4.933964	-2.497276	-2.315185
H	5.236254	-3.439069	-2.349841
H	5.292867	-2.047762	-3.119555
O	5.747466	-5.085901	-2.327377
H	6.734489	-5.074290	-2.265696
H	5.419149	-5.565243	-1.527801
O	5.000419	-6.377637	-4.618374
H	5.434276	-7.269175	-4.575816
H	5.273304	-5.907999	-3.793635
O	5.868269	-5.025207	-6.890855
H	5.553469	-5.479785	-6.078411
H	5.538578	-4.101282	-6.838522
O	4.864682	5.188062	6.755683
H	5.143082	4.245048	6.748737
H	3.876704	5.190802	6.731036
O	5.979992	6.202604	4.535570
H	6.938579	5.996939	4.627088
H	5.539605	5.834533	5.349491
O	5.105770	5.009973	2.262328
H	5.418073	5.436619	3.100977
H	5.464651	4.089295	2.264151
O	6.098960	2.455171	2.223122
H	5.734741	2.010609	3.029285
H	5.680736	2.000111	1.451374
O	4.954089	1.212366	0.075305
H	5.285222	0.282215	0.016603
H	3.965708	1.169708	0.084499
O	5.597084	2.507625	-2.210856
H	5.301291	2.068397	-1.374409
H	5.332590	3.460237	-2.171640
O	4.884383	1.330713	-4.545760
H	5.113503	1.767887	-3.689573
H	3.895785	1.328110	-4.595447
O	5.876765	-1.191993	-4.517654
H	6.861028	-1.118758	-4.500253
H	5.522999	-0.267233	-4.521645
O	5.034619	-2.381506	-6.827480
H	5.321133	-1.963013	-5.979050
H	4.046693	-2.362294	-6.837013
O	6.317378	-1.054101	-8.799433
H	6.367226	-1.621944	-9.582756
H	5.824389	-1.570383	-8.109543
O	5.172575	8.869485	4.399699
H	5.503842	7.945583	4.423308
H	5.567524	9.287206	3.599270

O	6.251270	10.087175	2.183193
H	7.219445	9.945949	2.169505
H	5.901986	9.681154	1.359508
O	5.228205	8.891604	-0.108324
H	5.677220	9.252042	-0.903917
H	5.497950	7.941842	-0.052413
O	5.957468	6.274133	0.017592
H	6.938489	6.180274	-0.024988
H	5.662823	5.822703	0.848443
O	4.926129	5.134955	-2.220781
H	5.297467	5.569499	-1.413261
H	5.315660	5.594659	-3.004506
O	5.979446	6.391844	-4.408478
H	6.964514	6.359816	-4.349400
H	5.713379	5.924935	-5.247874
O	5.182275	5.203994	-6.671881
H	5.466803	4.261497	-6.702264
H	4.202289	5.200490	-6.708610
O	5.995444	2.573951	-6.714781
H	6.974365	2.464014	-6.666269
H	5.615662	2.120768	-5.924156
O	5.074839	1.446605	-9.069253
H	5.462127	0.547009	-9.057151
H	5.389702	1.866515	-8.237619
O	1.487668	10.344747	-2.264117
H	1.814785	9.875737	-1.464596
H	0.510404	10.342614	-2.204836
O	2.416772	9.039590	-4.478226
H	3.395568	9.055910	-4.445106
H	2.094027	9.523944	-3.679848
O	1.309913	-9.143735	4.214047
H	1.654389	-9.664672	4.955600
H	1.675192	-9.566464	3.382302
O	2.243362	-10.247951	1.990930
H	3.220012	-10.224714	1.912192
H	1.884703	-9.815539	1.180286
O	1.317950	-1.387970	8.917640
H	1.616789	-1.829488	9.727267
H	0.331227	-1.405998	8.944943
O	2.258993	-2.699727	6.652842
H	1.915388	-2.265031	7.465244
H	1.895106	-3.627114	6.648559
O	1.340405	-5.221582	6.639813
H	1.641235	-5.696964	5.834577
H	0.353022	-5.242034	6.621692
O	2.250431	-6.538587	4.364126
H	1.905098	-7.458419	4.307625
H	1.921587	-6.065669	3.560751
O	1.309336	-5.209174	2.157088
H	1.600106	-4.264331	2.164489
H	0.321089	-5.194504	2.179937
O	2.102498	-6.423843	-0.172492
H	3.090788	-6.448057	-0.148392
H	1.815763	-5.972072	0.658560
O	1.240823	-9.024541	-0.258102
H	1.514737	-8.076620	-0.226381
H	0.251632	-9.032777	-0.260823
O	2.234913	-10.264625	-2.509833

H	1.868080	-9.805293	-1.719005
H	1.890644	-9.789457	-3.296353
O	1.324764	-8.925702	-4.782040
H	1.657631	-9.348508	-5.587973
H	1.698059	-8.006111	-4.778574
O	2.298449	1.276216	8.893884
H	1.950418	0.361928	8.883804
H	1.954322	1.709947	8.080547
O	1.367963	2.543165	6.623660
H	1.688254	2.078884	5.813139
H	0.383485	2.502089	6.604631
O	2.288643	1.238205	4.402902
H	3.277112	1.280696	4.393776
H	1.978155	1.704216	3.587930
O	1.362706	-1.333964	4.447758
H	1.709674	-1.801377	5.246522
H	1.723611	-0.413570	4.464003
O	2.157465	-2.612182	2.159432
H	1.852679	-2.124418	2.963120
H	1.822470	-2.123887	1.367216
O	1.327778	-1.318905	-0.088607
H	1.634667	-1.781509	-0.907451
H	0.337981	-1.315354	-0.116083
O	2.208355	-2.529334	-2.366852
H	3.197826	-2.524993	-2.358430
H	1.929808	-3.477215	-2.400236
O	1.342108	-5.118837	-2.446839
H	1.627054	-5.604646	-1.633864
H	1.705574	-5.607946	-3.223248
O	2.291386	-6.406890	-4.683813
H	3.283717	-6.399371	-4.692856
H	1.987071	-5.906175	-5.474142
O	1.306172	-5.039616	-6.896623
H	1.573078	-5.480695	-7.717832
H	0.317405	-5.021709	-6.907069
O	2.133439	5.206226	6.676481
H	1.840612	4.269255	6.625349
H	1.862015	5.629984	5.832115
O	1.413358	6.476558	4.319204
H	1.782955	5.999239	3.539060
H	1.800336	7.392062	4.312661
O	2.372712	5.096127	2.148464
H	3.358398	5.035802	2.185474
H	2.031004	4.168290	2.160924
O	1.400536	2.549142	2.182224
H	1.673635	2.065686	1.363693
H	0.411391	2.563260	2.182061
O	2.238412	1.235230	-0.043646
H	1.882757	0.312657	-0.090778
H	1.955882	1.698296	-0.869520
O	1.409320	2.576734	-2.282453
H	1.703436	2.165205	-3.131680
H	1.716552	3.516372	-2.292915
O	2.174230	1.353774	-4.604258
H	1.838150	0.424168	-4.629270
H	1.854312	1.808677	-5.422494
O	1.317018	-1.233135	-4.603770
H	1.626861	-1.698469	-3.788251

H	0.328046	-1.248178	-4.583851
O	2.294701	-2.425855	-6.851430
H	1.958891	-3.348896	-6.859996
H	1.928135	-2.007087	-6.034413
O	1.352681	-1.133915	-9.143948
H	1.697367	-1.567770	-8.334377
H	1.715687	-0.215839	-9.139823
O	2.436407	8.965301	4.377550
H	3.424387	8.944127	4.358129
H	2.148586	9.475873	3.592205
O	1.500192	10.332196	2.110425
H	1.806349	11.250472	2.066262
H	1.882160	9.876110	1.316153
O	2.475945	9.015905	-0.041488
H	3.460745	8.961670	-0.083017
H	2.131741	8.089599	-0.025649
O	1.427805	6.487152	-0.002528
H	1.754861	5.974581	0.777551
H	0.441905	6.509527	0.060429
O	2.211303	5.192682	-2.281219
H	3.200037	5.199822	-2.243194
H	1.904185	5.655562	-1.463083
O	1.470616	6.536034	-4.538747
H	1.843051	7.458719	-4.486806
H	1.746502	6.067370	-3.712297
O	2.374647	5.270029	-6.736869
H	2.068960	5.782164	-7.500863
H	2.041013	5.749230	-5.931931
O	1.376683	2.685776	-6.840600
H	1.701432	3.613842	-6.802025
H	0.389293	2.730743	-6.865327
O	2.321745	1.427578	-9.150202
H	3.306827	1.433189	-9.109370
H	2.009663	1.884391	-8.339664
O	-1.337404	10.383908	-2.179526
H	-1.646326	11.299443	-2.100735
H	-1.717329	9.895195	-1.398243
O	-2.152050	9.202385	-4.476620
H	-1.846790	9.661198	-3.651907
H	-1.761930	9.692030	-5.217284
O	-1.430723	-9.106342	4.273651
H	-0.441894	-9.129193	4.255838
H	-1.693465	-9.617215	5.054493
O	-2.455937	-10.283791	1.931758
H	-2.120825	-9.876000	2.757440
H	-3.433952	-10.246734	1.979325
O	-1.433821	-1.372997	8.893278
H	-1.814310	-1.848594	9.647898
H	-1.776221	-1.831902	8.075569
O	-2.317314	-2.606930	6.689951
H	-2.005470	-2.141754	5.874835
H	-3.305710	-2.620412	6.657188
O	-1.396788	-5.235941	6.642291
H	-1.731147	-4.312694	6.639446
H	-1.755104	-5.661324	5.833292
O	-2.390486	-6.494234	4.356549
H	-2.088340	-7.429972	4.346162
H	-3.375357	-6.507435	4.406160

O	-1.427290	-5.188230	2.144894
H	-1.806136	-5.680677	2.912692
H	-1.724357	-5.650227	1.323466
O	-2.218064	-6.407346	-0.177557
H	-1.930041	-7.353193	-0.237545
H	-1.886084	-5.950527	-0.988736
O	-1.485070	-9.019777	-0.267652
H	-1.853062	-9.490750	0.522336
H	-1.849205	-9.470429	-1.074593
O	-2.424865	-10.218783	-2.495848
H	-2.091565	-9.756017	-3.302358
H	-3.402432	-10.190749	-2.542295
O	-1.484740	-8.932648	-4.719020
H	-0.505330	-8.909665	-4.743147
H	-1.783028	-7.996599	-4.710996
O	-2.197117	1.260617	8.946519
H	-1.911075	0.314902	8.940845
H	-1.809382	1.655150	9.742368
O	-1.375258	2.545385	6.644383
H	-1.691831	2.126654	7.477074
H	-1.626107	3.496584	6.679814
O	-2.244789	1.268649	4.390078
H	-1.930285	1.714421	5.213175
H	-1.923038	0.334276	4.428856
O	-1.371970	-1.322785	4.463046
H	-0.382892	-1.329355	4.462602
H	-1.654335	-1.787665	3.637084
O	-2.205594	-2.563092	2.174792
H	-1.942207	-3.515609	2.158760
H	-3.195412	-2.546271	2.155151
O	-1.373870	-1.282086	-0.079225
H	-1.685511	-1.727896	0.747130
H	-1.684960	-0.343818	-0.045013
O	-2.262684	-2.495000	-2.346138
H	-1.922569	-2.080803	-1.515191
H	-1.934588	-3.427577	-2.359550
O	-1.390033	-5.085363	-2.424080
H	-0.403658	-5.112815	-2.485046
H	-1.741180	-5.529349	-3.234465
O	-2.394970	-6.308216	-4.657584
H	-2.108822	-5.827332	-5.466391
H	-3.383067	-6.306094	-4.654730
O	-1.442410	-4.968744	-6.899204
H	-1.785625	-5.373381	-7.710834
H	-1.782198	-4.030666	-6.894121
O	-1.980573	5.240852	6.671631
H	-1.516326	5.698724	7.388977
H	-1.698427	5.687107	5.823313
O	-1.289363	6.443246	4.396727
H	-0.300766	6.465886	4.317478
H	-1.630659	5.962258	3.602884
O	-2.255869	5.147853	2.195361
H	-1.934348	4.212357	2.193620
H	-3.242890	5.106077	2.224630
O	-1.320591	2.592633	2.192187
H	-1.652526	2.089766	2.975981
H	-1.643263	2.116980	1.387098
O	-2.197024	1.312375	-0.043874

H	-1.911226	1.785642	-0.863772
H	-3.186096	1.326481	-0.031268
O	-1.317917	2.626938	-2.283106
H	-0.329153	2.584042	-2.285488
H	-1.616204	2.188919	-3.118410
O	-2.158235	1.419662	-4.585558
H	-1.839882	1.889175	-5.396208
H	-3.145991	1.440482	-4.635602
O	-1.406687	-1.204324	-4.598458
H	-1.734510	-1.633524	-3.770734
H	-1.690984	-0.257434	-4.572475
O	-2.329563	-2.443609	-6.847890
H	-2.022938	-1.989947	-6.024696
H	-3.318388	-2.394431	-6.846478
O	-1.386701	-1.099396	-9.109947
H	-0.399071	-1.118059	-9.105337
H	-1.679550	-1.581149	-8.306923
O	-2.325002	9.016697	4.389510
H	-1.947436	8.108986	4.386766
H	-1.949484	9.474962	3.598922
O	-1.322289	10.303688	2.196059
H	-0.344431	10.286362	2.133306
H	-1.664581	9.879120	1.379337
O	-2.335154	9.054537	-0.079536
H	-2.012214	8.120470	-0.046886
H	-3.323471	9.023525	-0.086637
O	-1.318347	6.522433	0.020139
H	-1.680018	6.010252	0.784257
H	-1.577853	6.035819	-0.799400
O	-2.035335	5.255748	-2.294713
H	-1.751122	4.308581	-2.331680
H	-1.703657	5.699207	-3.111723
O	-1.237932	6.575213	-4.562584
H	-0.246436	6.600145	-4.571699
H	-1.544121	7.508904	-4.513208
O	-2.245039	5.350944	-6.755416
H	-1.936795	5.844487	-7.530499
H	-1.862580	5.814976	-5.965838
O	-1.351489	2.721922	-6.839165
H	-1.688203	3.645962	-6.839906
H	-1.739362	2.265820	-7.635217
O	-2.339038	1.448045	-8.983399
H	-1.997968	0.520511	-9.035494
H	-3.316823	1.388869	-9.007502
O	-6.319115	9.959718	-2.283702
H	-5.816794	9.724649	-3.090806
H	-7.223122	9.601223	-2.404580
O	-4.895237	9.183934	-4.583911
H	-3.908841	9.227017	-4.559769
H	-5.168642	9.658508	-5.383459
O	-6.148831	-8.927836	4.218129
H	-5.915255	-9.388872	5.038075
H	-5.773933	-8.010160	4.297457
O	-5.269812	-10.210894	1.980939
H	-5.593546	-9.773742	2.807158
H	-5.632078	-11.110022	1.991043
O	-6.059367	-1.160746	8.814974
H	-5.701274	-1.650556	8.043820

H	-5.633894	-0.270995	8.809262
O	-5.053623	-2.601567	6.633135
H	-5.508472	-3.471118	6.576577
H	-5.302294	-2.109937	5.811187
O	-6.412388	-5.047292	6.449856
H	-6.302200	-5.542549	7.276004
H	-7.394730	-4.931183	6.336081
O	-5.148293	-6.428845	4.382956
H	-5.585210	-5.931225	5.111847
H	-5.358552	-5.938922	3.546254
O	-5.711699	-5.079071	2.111525
H	-5.411917	-5.541418	1.289733
H	-6.701072	-5.029974	2.057135
O	-4.932067	-6.359906	-0.163668
H	-3.942801	-6.376081	-0.206821
H	-5.246175	-5.844086	-0.947636
O	-6.035847	-8.834209	-0.294797
H	-5.794405	-9.315663	0.529334
H	-5.605222	-7.943329	-0.239312
O	-5.246737	-10.159015	-2.502768
H	-5.589180	-11.063824	-2.439133
H	-5.522717	-9.699834	-1.665165
O	-6.462033	-8.724419	-4.483814
H	-6.425716	-9.199659	-5.327805
H	-6.007268	-9.300846	-3.821096
O	-5.012267	1.367444	8.872771
H	-4.033783	1.368829	8.893120
H	-5.276288	1.858405	8.063890
O	-5.812707	2.756340	6.597391
H	-5.479358	2.282315	5.796354
H	-5.355027	3.625435	6.628234
O	-4.966806	1.362046	4.407498
H	-3.979303	1.356414	4.351617
H	-5.307820	1.784848	3.582296
O	-5.750762	-1.251617	4.383713
H	-5.455443	-0.307996	4.404915
H	-6.740565	-1.225705	4.414394
O	-4.920676	-2.480918	2.101679
H	-5.216359	-3.425140	2.126417
H	-5.240980	-2.054593	2.935179
O	-5.953799	-1.220110	-0.086614
H	-5.597630	-1.675867	0.716016
H	-5.595138	-0.298993	-0.060816
O	-4.980517	-2.430403	-2.319863
H	-3.991120	-2.441597	-2.294416
H	-5.290818	-1.985631	-1.492146
O	-5.882372	-4.989034	-2.312285
H	-5.553625	-4.055185	-2.314724
H	-6.866565	-4.933939	-2.247072
O	-5.134168	-6.277989	-4.593546
H	-5.570131	-7.159087	-4.570755
H	-5.420176	-5.808356	-3.771333
O	-5.974169	-4.873240	-6.832235
H	-5.681812	-5.372802	-6.037279
H	-6.953995	-4.868185	-6.802565
O	-4.689295	5.283108	6.783072
H	-3.700029	5.310102	6.804023
H	-4.995287	5.729437	7.587242

O	-5.838782	6.374435	4.519733
H	-5.386636	5.998667	5.309098
H	-5.576793	7.325022	4.491412
O	-4.991355	5.088685	2.246189
H	-5.311888	5.582878	3.038423
H	-5.278173	5.591456	1.444459
O	-5.982673	2.554846	2.158183
H	-5.692990	3.499987	2.188205
H	-6.969722	2.548375	2.139955
O	-4.919015	1.304717	-0.021673
H	-5.312149	1.794666	0.741780
H	-5.138109	1.813786	-0.839200
O	-5.464753	2.666127	-2.306977
H	-5.225210	2.242418	-3.169177
H	-5.170734	3.610802	-2.344747
O	-4.871858	1.442520	-4.643373
H	-5.220037	0.516325	-4.618442
H	-5.273668	1.883241	-5.432447
O	-5.824510	-1.092132	-4.530276
H	-5.528792	-1.568781	-3.715224
H	-6.809507	-1.043277	-4.492828
O	-5.061989	-2.330619	-6.842503
H	-5.398924	-3.264172	-6.824591
H	-5.331275	-1.924933	-5.983593
O	-6.458382	-0.847029	-8.718122
H	-7.339139	-0.612510	-8.347597
H	-5.991580	-1.394489	-8.045849
O	-4.971716	8.988380	4.442589
H	-3.969173	9.003680	4.420303
H	-5.231285	9.483546	5.234185
O	-6.075092	10.139333	2.153419
H	-5.681724	9.748175	2.963077
H	-7.042882	9.995916	2.223260
O	-5.072612	8.980373	-0.072097
H	-5.525035	9.372567	-0.860906
H	-5.447880	9.424330	0.732612
O	-5.747492	6.357270	-0.040231
H	-5.502117	7.317586	-0.047169
H	-5.347248	5.959571	-0.852918
O	-4.742921	5.269586	-2.311414
H	-3.754608	5.312977	-2.346069
H	-5.093270	5.757353	-3.097262
O	-5.824847	6.576576	-4.447360
H	-5.476257	7.492908	-4.527310
H	-5.555770	6.104686	-5.281857
O	-5.053090	5.326429	-6.692000
H	-4.073546	5.292847	-6.715597
H	-5.369630	4.395949	-6.732835
O	-5.968908	2.717884	-6.785365
H	-5.693487	2.293474	-7.631790
H	-6.953038	2.647160	-6.737681
O	-5.148899	1.431401	-9.068492
H	-5.622010	0.552596	-8.989590
H	-5.452556	1.833126	-9.896318
O	-8.966163	8.982002	-2.573220
H	-9.519502	9.674345	-2.965597
H	-9.319980	8.833991	-1.659705
O	-11.467404	7.061157	1.889213

H	-10.967329	6.219281	1.943666
H	-11.054995	7.555790	1.151248
O	-8.944536	-8.810459	3.846117
H	-7.973967	-8.854052	3.964651
H	-9.198713	-7.871510	3.984256
O	-10.303613	-9.214561	1.507160
H	-9.690302	-9.169259	2.278540
H	-9.755332	-9.132618	0.700435
O	-8.757744	-0.838069	8.723809
H	-7.771583	-0.920338	8.738216
H	-8.972387	0.098226	8.541773
O	-9.851171	-2.101779	6.612019
H	-10.598297	-1.500864	6.415622
H	-9.423558	-1.708092	7.419202
O	-9.090646	-4.784309	6.223574
H	-9.504049	-5.213590	5.444612
H	-9.462530	-3.881448	6.299898
O	-9.865479	-6.182821	3.932166
H	-10.683420	-6.330375	3.413052
H	-9.263408	-5.733988	3.286659
O	-8.386120	-4.918741	2.052413
H	-8.760274	-5.331695	1.237379
H	-8.754877	-3.993572	2.099457
O	-9.649107	-6.126871	-0.033344
H	-9.302444	-7.034332	-0.230600
H	-10.497677	-6.253504	0.443769
O	-8.745246	-8.570897	-0.754603
H	-7.776778	-8.667385	-0.599157
H	-8.881357	-8.548662	-1.733447
O	-11.585069	-6.875379	1.816671
H	-11.252966	-7.807814	1.713771
H	-12.549183	-6.903708	1.730302
O	-9.084571	-8.405168	-3.488295
H	-8.199524	-8.478652	-3.903486
H	-9.482044	-7.584366	-3.840936
O	-9.904473	1.654798	7.962673
H	-10.553621	1.206609	7.372437
H	-10.416947	2.024759	8.697485
O	-8.464639	3.294204	6.193637
H	-7.512363	3.096810	6.373872
H	-8.964218	2.926517	6.953721
O	-9.411964	1.368847	4.472377
H	-9.270210	1.710425	3.554702
H	-9.004762	2.049261	5.061075
O	-8.442868	-1.175586	4.461328
H	-8.851897	-1.586850	5.260524
H	-8.726552	-0.229130	4.482751
O	-9.461968	-2.481448	2.240480
H	-9.096836	-1.991250	3.010596
H	-9.203643	-1.976901	1.436572
O	-8.702030	-1.232403	-0.088895
H	-7.713691	-1.219967	-0.106209
H	-8.994323	-0.292924	-0.114948
O	-9.624494	-2.348024	-2.392474
H	-9.154502	-1.822720	-3.085194
H	-9.373526	-1.939118	-1.527486
O	-8.599194	-4.823431	-2.213296
H	-8.916407	-3.884671	-2.257461

H	-9.056373	-5.230134	-1.442497
O	-9.805821	-5.793653	-4.422489
H	-10.603922	-5.226717	-4.397394
H	-9.275600	-5.461331	-3.653864
O	-8.775310	-4.805631	-6.740572
H	-9.152881	-5.306292	-7.479920
H	-9.101712	-5.246449	-5.914128
O	-11.307056	0.246788	6.036092
H	-10.816782	0.666253	5.289243
H	-12.251009	0.393797	5.874611
O	-8.521104	5.748645	4.738363
H	-7.563530	5.978221	4.682801
H	-8.586181	4.962375	5.323694
O	-9.557234	5.043584	2.287255
H	-9.136219	5.315056	3.137764
H	-9.010187	5.420883	1.557154
O	-8.740062	2.475007	2.113926
H	-9.099143	3.397383	2.150842
H	-9.087300	2.072879	1.285187
O	-9.554605	1.366277	-0.241048
H	-10.482826	1.457370	-0.503755
H	-9.012091	1.832502	-0.931136
O	-8.119675	2.627477	-2.099018
H	-7.127924	2.641886	-2.159367
H	-8.442388	3.560039	-2.148633
O	-9.487030	1.580831	-4.147752
H	-8.904918	1.929035	-3.424845
H	-10.328042	2.073989	-4.044971
O	-8.544049	-0.968528	-4.458747
H	-8.969386	-0.083019	-4.400886
H	-8.974344	-1.441850	-5.211444
O	-9.951850	-2.308336	-6.356762
H	-10.639228	-2.626950	-5.732560
H	-9.474458	-3.125670	-6.624752
O	-8.981364	-0.045198	-7.890624
H	-9.480289	-0.680734	-7.343640
H	-9.012862	0.826975	-7.449674
O	-9.995796	8.080955	4.008365
H	-10.630865	7.710485	3.353105
H	-9.505986	7.316305	4.373247
O	-8.846961	9.786339	2.259595
H	-9.272651	10.643060	2.415987
H	-9.180927	9.180976	2.974114
O	-9.753864	8.402996	0.000575
H	-9.404740	9.012759	0.687612
H	-9.184634	7.595548	0.055396
O	-8.420853	6.062512	0.060937
H	-7.438719	6.137938	-0.051174
H	-8.790046	5.610577	-0.729226
O	-9.415342	4.972595	-2.271768
H	-10.243109	4.589350	-2.632994
H	-9.063123	5.570319	-2.978318
O	-8.534252	6.692632	-4.165273
H	-7.550398	6.660417	-4.274306
H	-8.737724	7.538839	-3.708323
O	-9.927604	5.022556	-5.899502
H	-9.426476	5.709609	-5.401323
H	-10.428402	5.496897	-6.580664

O	-8.695528	2.511020	-6.566127
H	-9.084666	3.409190	-6.579828
H	-8.900478	2.180604	-5.657116
O	-11.283924	3.685635	-3.899672
H	-12.250846	3.739924	-3.873761
H	-10.989096	4.177930	-4.700719
O	-11.392335	-3.450331	-4.210953
H	-12.349620	-3.364705	-4.086822
H	-10.971267	-3.032150	-3.426747
O	11.212219	-3.734541	-4.398035
H	10.843777	-3.323212	-3.591795
H	10.865946	-3.204139	-5.142814

Table S7. M06-2X/6-31+G*/PCM optimized Cartesian coordinates of the $(H_2O)_{332}$ hexagonal cluster.

O	-8.810322	6.869150	-8.452568
H	-9.018478	7.408500	-7.663508
H	-7.835785	6.870128	-8.559073
O	-9.905277	8.201385	-6.207697
H	-10.289710	9.067631	-6.409886
H	-9.446600	8.292019	-5.338858
O	-9.209803	-9.475577	2.656216
H	-9.525480	-10.339222	2.963194
H	-9.531185	-8.821173	3.322776
O	-9.776455	-7.575846	4.556352
H	-10.599004	-7.141403	4.860662
H	-9.202556	-6.825219	4.254146
O	-8.786131	-8.036987	7.123397
H	-9.108661	-7.927953	6.202144
H	-9.123893	-7.262259	7.615232
O	-10.093065	-5.844906	8.382587
H	-10.490141	-6.067202	9.238130
H	-9.621014	-4.987832	8.503643
O	-8.631793	-3.530329	8.217371
H	-8.707651	-2.598583	8.523684
H	-7.676779	-3.777621	8.266918
O	-11.418875	1.987885	7.775313
H	-11.094517	2.891676	7.584489
H	-10.926721	1.391379	7.171826
O	-9.931761	-9.419054	-3.950220
H	-10.557608	-8.907458	-3.399139
H	-9.468896	-8.766466	-4.512924
O	-8.798307	-10.770039	-1.811110
H	-9.034891	-10.323288	-2.654600
H	-7.822288	-10.841029	-1.777179
O	-9.789286	-8.997854	-0.048087
H	-9.677751	-9.205976	0.905674
H	-9.386773	-9.734480	-0.578277
O	-8.370622	-6.739901	-0.547265
H	-8.825418	-7.598417	-0.361158
H	-8.643711	-6.485599	-1.459461
O	-9.372429	-4.922706	1.277371
H	-9.023504	-5.597480	0.653307
H	-9.001512	-5.134289	2.162829

O	-8.354573	-5.421279	3.792270
H	-8.640529	-4.774713	4.477761
H	-7.368034	-5.490370	3.848292
O	-9.560733	-3.822444	5.649997
H	-10.382618	-4.355812	5.704076
H	-9.214605	-3.730303	6.570174
O	-8.887988	-1.390228	4.613304
H	-9.223151	-2.264951	4.915382
H	-7.902454	-1.431164	4.675648
O	-9.491807	0.436538	6.523525
H	-9.014948	1.258606	6.258495
H	-9.354951	-0.212709	5.791555
O	-8.663601	-0.757902	8.818768
H	-8.892758	-0.340545	7.949082
H	-9.196614	-0.239820	9.458480
O	-10.171660	1.283473	10.053173
H	-10.744143	1.509185	9.270507
H	-10.772793	1.102535	10.791888
O	-9.066817	3.815228	10.118854
H	-9.488496	4.318500	10.832048
H	-9.396905	2.889308	10.209000
O	-11.359392	-7.919701	-2.021403
H	-11.004637	-8.287806	-1.179972
H	-12.322725	-7.875927	-1.930117
O	-8.491122	-7.327360	-5.226271
H	-8.528325	-6.737227	-6.010962
H	-7.541678	-7.551853	-5.075384
O	-9.469149	-6.118605	-2.981211
H	-9.109366	-6.548936	-3.796253
H	-10.306693	-6.575012	-2.756966
O	-8.809690	-3.513224	-3.409323
H	-9.173337	-4.396131	-3.168049
H	-9.216072	-2.857239	-2.800440
O	-9.635973	-1.649207	-1.573236
H	-9.107635	-1.992709	-0.807949
H	-10.370017	-1.136535	-1.159555
O	-8.351465	-2.564037	0.589339
H	-8.695351	-3.464695	0.840653
H	-7.363804	-2.590922	0.624509
O	-9.625343	-0.710259	2.074451
H	-9.465840	-0.936243	3.020861
H	-9.110322	-1.385337	1.565525
O	-8.560017	1.721764	1.616912
H	-8.896011	0.810574	1.822419
H	-8.876880	1.912925	0.695866
O	-9.558054	3.469041	3.370794
H	-9.228561	2.809405	2.699812
H	-10.525932	3.426732	3.344854
O	-8.540136	2.901235	5.865848
H	-9.010036	3.494623	6.503958
H	-8.908447	3.108080	4.975684
O	-10.002776	4.428162	7.562545
H	-9.628252	4.392602	8.469150
H	-9.867523	5.345787	7.213764
O	-9.412014	6.880646	6.542418
H	-9.728261	7.182687	5.668465
H	-8.429924	6.969583	6.537531
O	-9.859798	-4.383053	-9.509946

H	-10.292161	-4.966489	-10.151462
H	-9.342168	-3.728390	-10.037190
O	-8.424605	-5.401184	-7.320385
H	-8.941011	-5.238208	-8.138821
H	-7.480365	-5.249936	-7.570306
O	-9.356240	-3.134955	-6.051490
H	-8.956828	-3.940992	-6.456168
H	-9.228270	-3.231006	-5.077590
O	-8.358973	-0.740467	-6.858725
H	-8.663736	-1.637238	-6.570884
H	-8.782185	-0.583666	-7.735975
O	-9.383637	1.149324	-5.124394
H	-9.029548	0.471043	-5.742322
H	-9.039976	0.902457	-4.235379
O	-8.395731	0.510682	-2.650082
H	-8.725379	-0.353877	-2.305451
H	-7.407579	0.498580	-2.608545
O	-9.582755	2.137864	-0.842672
H	-9.092315	1.598211	-1.511639
H	-9.407225	3.077591	-1.087220
O	-8.847552	4.664588	-1.543616
H	-9.168365	4.893617	-2.446655
H	-7.860922	4.715947	-1.573411
O	-9.572691	6.548604	0.263574
H	-9.107166	6.308150	1.098628
H	-9.391452	5.818687	-0.376626
O	-8.560721	5.960160	2.742728
H	-9.027716	6.647322	3.277345
H	-8.935891	5.087435	3.002564
O	-10.040766	7.907566	3.946949
H	-10.709339	7.911469	3.219094
H	-9.569424	8.762944	3.838736
O	-8.881137	10.365487	3.311848
H	-9.262474	11.078811	3.846651
H	-7.905501	10.395125	3.476810
O	-8.648796	-2.300585	-10.857182
H	-8.964511	-2.241965	-11.771837
H	-7.653899	-2.188588	-10.901019
O	-9.827971	-0.453598	-9.152954
H	-10.512152	-1.052240	-8.768649
H	-9.382407	-1.003214	-9.836398
O	-9.009108	2.245368	-9.418927
H	-9.427874	1.378697	-9.247839
H	-9.462844	2.915466	-8.870816
O	-9.896520	4.453569	-7.876330
H	-10.642853	4.774161	-7.329221
H	-9.452011	5.287331	-8.176101
O	-8.412399	3.550197	-5.770129
H	-8.868302	3.823148	-6.602378
H	-8.769095	2.648306	-5.535287
O	-9.496085	5.365029	-4.095612
H	-9.016650	4.712633	-4.669204
H	-10.359940	5.489146	-4.542409
O	-8.557154	7.929008	-3.830108
H	-8.688277	8.287278	-2.923525
H	-8.813815	6.977059	-3.807325
O	-11.502848	8.035018	1.608949
H	-11.026937	7.383202	1.053332

H	-11.173941	8.899174	1.285780
O	-8.702334	8.737682	-1.111019
H	-8.958670	7.927739	-0.602188
H	-9.173007	9.458523	-0.642392
O	-10.048442	10.316350	0.792336
H	-9.535180	10.430148	1.625147
H	-10.474318	11.168752	0.616826
O	-6.033383	6.813128	-8.845421
H	-5.823105	7.098426	-9.747706
H	-5.660779	7.504082	-8.243395
O	-5.121262	8.669363	-7.077923
H	-5.456013	9.555214	-7.284760
H	-5.460046	8.450151	-6.168104
O	-6.345853	-9.680921	2.815552
H	-7.310472	-9.549468	2.714855
H	-5.941389	-9.574825	1.926286
O	-5.081523	-8.128572	4.671390
H	-5.506571	-8.711020	3.992931
H	-4.102906	-8.219783	4.586481
O	-6.097579	-8.520206	7.218247
H	-7.065691	-8.338705	7.151035
H	-5.724908	-8.391920	6.316696
O	-5.159960	-6.797037	8.967903
H	-5.517156	-7.070464	9.826821
H	-5.503743	-7.464390	8.297801
O	-5.976931	-4.245096	8.292091
H	-5.743573	-5.171386	8.534554
H	-5.562425	-3.670369	8.977670
O	-4.966716	-2.499396	10.149555
H	-5.324994	-2.645293	11.038107
H	-5.341953	-1.633927	9.835108
O	-4.994990	-10.517950	-4.026240
H	-5.304048	-11.151133	-4.692431
H	-4.007013	-10.560987	-4.040587
O	-6.011063	-11.068528	-1.552076
H	-5.826346	-11.992390	-1.324044
H	-5.601842	-10.918231	-2.438370
O	-5.017003	-9.335409	0.388373
H	-5.359261	-9.943361	-0.304836
H	-5.258600	-8.420051	0.097267
O	-5.685880	-6.815205	-0.353993
H	-6.674988	-6.774905	-0.373664
H	-5.382020	-6.153539	0.315611
O	-4.786375	-4.994141	1.457897
H	-5.077378	-5.214171	2.377263
H	-3.796967	-5.019005	1.460606
O	-5.674029	-5.589945	3.951483
H	-5.430006	-6.499682	4.257637
H	-5.405827	-4.949144	4.655497
O	-5.014717	-3.695685	5.778084
H	-5.325520	-3.889618	6.694617
H	-4.025667	-3.674504	5.803891
O	-6.135839	-1.354207	4.875542
H	-5.740000	-2.211581	5.165041
H	-5.704095	-1.125094	4.015684
O	-5.101916	0.449554	6.675138
H	-5.490079	-0.198462	6.037745
H	-5.431851	0.200460	7.571060

O	-6.002267	-0.221888	9.180289
H	-6.981733	-0.342836	9.116958
H	-5.817789	0.507447	9.818435
O	-5.214347	1.711428	10.968149
H	-5.482586	1.493359	11.873528
H	-4.213187	1.733531	10.974260
O	-6.274387	4.167964	10.172156
H	-7.244166	4.031207	10.147125
H	-5.891238	3.314051	10.467021
O	-4.807554	-7.489526	-7.219343
H	-5.151664	-7.652798	-6.314401
H	-5.136954	-6.596441	-7.464033
O	-5.871672	-7.937669	-4.686815
H	-5.604024	-8.840663	-4.411632
H	-5.520843	-7.311340	-4.006533
O	-4.965990	-6.113722	-2.880082
H	-5.210151	-6.359394	-1.953195
H	-3.977836	-6.085710	-2.896095
O	-6.024293	-3.687246	-3.589335
H	-7.005549	-3.653136	-3.509634
H	-5.725629	-4.589132	-3.317618
O	-4.876975	-1.921717	-1.850288
H	-5.298286	-2.567322	-2.468630
H	-5.164381	-2.167670	-0.935876
O	-5.647397	-2.547021	0.671845
H	-5.307369	-3.419915	0.990547
H	-5.380601	-1.866328	1.338406
O	-4.970485	-0.660455	2.505121
H	-5.281651	0.234001	2.218302
H	-3.982376	-0.619169	2.529536
O	-5.845056	1.794046	1.725004
H	-6.833787	1.792620	1.714300
H	-5.560100	2.477698	2.380977
O	-4.946031	3.643242	3.503180
H	-5.278382	3.433992	4.411380
H	-5.282442	4.543046	3.266286
O	-5.841972	2.987067	5.986008
H	-6.829844	2.965898	5.940175
H	-5.562600	2.074748	6.243335
O	-5.240947	4.948548	7.782749
H	-5.618942	4.670489	8.655675
H	-5.442859	4.211310	7.156923
O	-6.668399	7.126675	6.749004
H	-6.175087	6.363677	7.122467
H	-6.128132	7.460952	6.001107
O	-4.836018	-4.420207	-10.407378
H	-5.103995	-5.100688	-11.043038
H	-3.834734	-4.403008	-10.416922
O	-5.783625	-4.984723	-7.874095
H	-5.468468	-4.746505	-8.775754
H	-5.438239	-4.301116	-7.250210
O	-4.912958	-3.144422	-6.059098
H	-5.289188	-3.337531	-5.168230
H	-3.926274	-3.156392	-5.983883
O	-5.663568	-0.644118	-6.830278
H	-6.653390	-0.671884	-6.848305
H	-5.374883	-1.550222	-6.559218
O	-4.809501	1.219475	-5.058354

H	-5.137326	0.526530	-5.685211
H	-5.132930	0.968473	-4.158089
O	-5.691304	0.568588	-2.568010
H	-5.364212	-0.314782	-2.267878
H	-5.413018	1.232703	-1.888894
O	-4.975935	2.460690	-0.763965
H	-5.281107	2.223099	0.146696
H	-3.987389	2.489825	-0.729313
O	-6.088555	4.839961	-1.526093
H	-5.685801	3.973117	-1.272826
H	-5.712302	5.071697	-2.410660
O	-5.085539	6.693822	0.226608
H	-5.400825	6.008290	-0.411589
H	-4.097216	6.689504	0.213515
O	-5.861968	6.097335	2.765180
H	-6.849715	6.048662	2.760826
H	-5.589139	6.306029	1.837301
O	-5.207604	8.043094	4.539595
H	-5.417279	7.354961	3.859635
H	-4.222450	8.056325	4.617361
O	-6.194729	10.414626	3.771195
H	-5.942266	11.067429	4.441591
H	-5.816689	9.542800	4.071529
O	-6.024281	-1.983459	-11.017173
H	-5.558027	-2.819097	-10.797754
H	-5.652185	-1.286131	-10.428763
O	-4.974528	-0.011592	-9.389853
H	-5.207642	-0.250428	-8.459550
H	-5.434681	0.849652	-9.568168
O	-6.253289	2.335482	-9.848662
H	-7.212448	2.319973	-9.633613
H	-5.847914	3.051940	-9.312860
O	-4.976715	4.288762	-8.314152
H	-5.333594	5.185104	-8.503680
H	-5.245125	4.077539	-7.384660
O	-5.722591	3.674464	-5.781686
H	-6.710205	3.598294	-5.794586
H	-5.383334	2.783181	-5.517066
O	-4.990572	5.532992	-3.936855
H	-5.286189	4.891353	-4.629882
H	-5.316570	6.426280	-4.204756
O	-5.901332	8.037473	-4.586398
H	-6.861750	8.026352	-4.361340
H	-5.493672	8.719901	-3.984836
O	-4.911487	9.916661	-2.956050
H	-5.234482	9.663345	-2.060806
H	-3.931872	9.927847	-2.916179
O	-6.021046	9.140667	-0.562234
H	-6.981146	9.013928	-0.748191
H	-5.679043	8.258480	-0.279887
O	-5.207539	11.096956	1.228347
H	-5.550225	10.855404	2.112931
H	-5.536125	10.405385	0.612383
O	-1.309346	6.853129	-8.948565
H	-1.618971	5.946695	-8.728116
H	-0.330970	6.819702	-8.934472
O	-2.300347	8.649022	-7.137687
H	-3.279223	8.629519	-7.160703

H	-1.977754	7.999680	-7.808577
O	-1.265366	-10.028295	2.835860
H	-1.543632	-10.913488	3.116800
H	-0.276936	-10.026020	2.877417
O	-2.333791	-8.141750	4.584362
H	-1.937330	-8.782133	3.953588
H	-1.983446	-7.250590	4.342811
O	-1.356868	-8.698085	7.133773
H	-1.719696	-8.493925	6.244068
H	-1.750698	-8.046449	7.748860
O	-2.425874	-6.835989	8.974479
H	-3.414860	-6.838322	9.001489
H	-2.131584	-7.079181	9.865402
O	-1.406133	-4.373394	8.210630
H	-1.779233	-5.236576	8.499115
H	-1.705426	-3.696368	8.875155
O	-2.174849	-2.525204	10.006475
H	-3.151407	-2.503825	10.087433
H	-1.908360	-1.618872	9.727060
O	-2.262296	-10.572845	-3.946632
H	-1.864550	-11.202129	-4.568048
H	-1.940556	-10.831587	-3.045951
O	-1.390131	-11.191578	-1.447123
H	-1.696520	-12.064118	-1.156337
H	-1.742392	-10.541426	-0.782477
O	-2.288148	-9.385147	0.339371
H	-3.277329	-9.381225	0.395251
H	-1.949122	-9.614775	1.234415
O	-1.301658	-6.907289	-0.387855
H	-1.645420	-7.797186	-0.138266
H	-0.314659	-6.970985	-0.396868
O	-2.075782	-5.022906	1.429093
H	-1.764319	-5.727538	0.810045
H	-1.766508	-5.263375	2.336770
O	-1.335126	-5.651789	3.983133
H	-1.671892	-4.962666	4.607215
H	-0.349196	-5.637639	4.050403
O	-2.291184	-3.750086	5.712685
H	-1.954048	-3.947828	6.621840
H	-1.944797	-2.857406	5.464933
O	-1.391000	-1.277423	4.994236
H	-1.673650	-1.049061	4.074199
H	-0.401564	-1.260508	4.999041
O	-2.364889	0.584512	6.723910
H	-3.348531	0.503536	6.689979
H	-2.009050	-0.079739	6.082829
O	-1.428408	0.012475	9.239267
H	-1.740435	0.208772	8.323905
H	-0.439710	0.046081	9.224886
O	-2.561915	1.794773	10.995175
H	-2.138463	1.164264	10.366801
H	-2.219628	2.685119	10.764959
O	-1.591450	4.334030	10.340574
H	-1.905228	4.996701	10.974262
H	-1.955912	4.604430	9.458314
O	-2.073377	-7.428296	-7.208643
H	-3.060692	-7.464567	-7.212613
H	-1.802877	-7.645857	-6.289852

O	-1.312971	-8.034281	-4.606388
H	-1.668703	-8.908196	-4.335313
H	-1.665749	-7.365227	-3.969867
O	-2.230349	-6.149901	-2.863801
H	-1.891347	-6.383003	-1.966362
H	-1.848587	-5.267949	-3.100860
O	-1.255189	-3.724697	-3.601472
H	-1.555720	-3.035546	-2.958156
H	-0.265300	-3.720629	-3.583467
O	-2.168839	-1.874623	-1.835857
H	-3.157413	-1.885559	-1.881732
H	-1.882120	-0.958903	-2.076436
O	-1.385830	-2.483183	0.698620
H	-1.659251	-3.404339	0.928085
H	-1.696044	-2.297415	-0.222166
O	-2.249878	-0.628429	2.500457
H	-1.941441	-1.282864	1.826918
H	-1.918863	0.260196	2.218932
O	-1.379910	1.852729	1.776039
H	-1.669121	2.521123	2.445223
H	-0.390217	1.856008	1.786762
O	-2.229415	3.714737	3.584331
H	-3.218897	3.689179	3.568437
H	-1.964414	3.501416	4.512173
O	-1.494401	3.112665	6.154513
H	-1.836452	2.213935	6.384762
H	-1.894210	3.752800	6.789831
O	-2.526230	4.990941	7.888972
H	-3.517147	5.024208	7.854584
H	-2.195231	5.880943	7.631899
O	-1.510566	7.484543	7.200814
H	-1.804047	8.164311	7.826685
H	-0.521856	7.514966	7.207795
O	-2.187009	-4.347829	-10.397033
H	-1.841527	-4.552165	-9.496337
H	-1.848613	-3.457256	-10.631948
O	-1.254168	-4.958680	-7.885635
H	-1.569088	-5.862270	-7.613428
H	-1.607526	-4.319843	-7.222155
O	-2.193784	-3.114283	-6.077865
H	-1.814728	-3.297933	-5.182835
H	-1.871382	-2.222089	-6.352343
O	-1.292324	-0.646936	-6.890442
H	-1.593381	0.042486	-6.249000
H	-1.653294	-0.391887	-7.772607
O	-2.096588	1.237365	-5.074129
H	-3.086659	1.227533	-5.059968
H	-1.811388	1.018372	-4.152993
O	-1.332970	0.630934	-2.520223
H	-1.658348	1.302248	-1.871229
H	-0.344827	0.654170	-2.473121
O	-2.255947	2.473714	-0.730522
H	-1.925263	2.279620	0.181225
H	-1.933023	3.377519	-0.969006
O	-1.406495	4.956577	-1.444361
H	-1.763848	5.636436	-0.821144
H	-1.728653	5.188974	-2.349906
O	-2.358422	6.785791	0.332354

H	-2.040561	6.592015	1.247905
H	-2.019299	7.685457	0.097880
O	-1.466088	6.242551	2.871094
H	-1.722933	5.324441	3.134081
H	-0.478025	6.265916	2.875805
O	-2.467319	8.070974	4.649786
H	-2.119853	7.848721	5.541380
H	-2.101626	7.394804	4.028878
O	-1.501164	10.587124	3.884369
H	-1.853372	9.707761	4.139216
H	-1.866838	10.782899	2.988729
O	-1.252502	-1.807944	-11.108020
H	-1.559065	-1.578348	-11.998212
H	-1.635010	-1.121514	-10.502024
O	-2.248142	0.029901	-9.390637
H	-3.238133	0.062745	-9.416560
H	-1.926282	0.929267	-9.633279
O	-1.270937	2.522706	-10.050396
H	-1.556367	2.799535	-10.934378
H	-1.638255	3.197073	-9.417519
O	-2.237046	4.318101	-8.300098
H	-3.226025	4.310719	-8.292638
H	-1.936088	4.102084	-7.383492
O	-1.310572	3.750590	-5.785741
H	-1.568581	2.833186	-5.519182
H	-0.322374	3.773418	-5.780371
O	-2.260584	5.559862	-3.963579
H	-3.249398	5.531722	-3.975973
H	-1.942502	4.889208	-4.617020
O	-1.373455	8.023964	-4.711813
H	-1.739909	8.226577	-5.616373
H	-1.713384	7.131576	-4.453746
O	-2.123462	9.900655	-2.946691
H	-1.737162	10.742716	-3.230396
H	-1.832764	9.219128	-3.610548
O	-1.417497	9.245201	-0.359099
H	-1.654676	9.471429	-1.287284
H	-0.429342	9.253618	-0.321303
O	-2.452683	11.165104	1.377390
H	-3.434933	11.124826	1.306304
H	-2.103412	10.495584	0.750394
O	1.526883	6.814166	-8.933243
H	1.854665	6.978375	-9.830778
H	1.885448	5.923383	-8.667970
O	2.322067	8.719308	-7.169867
H	2.038376	8.043651	-7.837779
H	1.933731	9.560522	-7.456091
O	1.467524	-9.970576	2.940401
H	1.821814	-10.819130	3.248039
H	1.804673	-9.282260	3.578179
O	2.383983	-8.106630	4.632265
H	2.077559	-7.196507	4.394845
H	3.370526	-8.091966	4.633336
O	1.401216	-8.682097	7.189954
H	0.416408	-8.671583	7.146346
H	1.723230	-8.486354	6.284312
O	2.294634	-6.781824	8.933877
H	1.998130	-7.479937	8.299549

H	3.273152	-6.785958	8.920977
O	1.335916	-4.370527	8.197718
H	0.349670	-4.418220	8.198753
H	1.670070	-5.266094	8.468412
O	2.087948	-2.455619	10.040402
H	1.819821	-3.144945	9.390316
H	3.067354	-2.478309	10.066846
O	2.464208	-10.479694	-3.936563
H	2.092587	-10.713861	-3.054322
H	2.075629	-9.608563	-4.174517
O	1.448906	-11.163783	-1.470960
H	0.470911	-11.147946	-1.444494
H	1.757519	-10.525897	-0.790500
O	2.410347	-9.348336	0.403688
H	2.104337	-9.579963	1.308904
H	3.397900	-9.385241	0.419295
O	1.433146	-6.908744	-0.378866
H	1.813138	-7.769009	-0.076321
H	1.691753	-6.229908	0.291983
O	2.133192	-5.004756	1.456413
H	1.822045	-4.101983	1.198008
H	3.122156	-4.961139	1.454447
O	1.409508	-5.633906	4.009293
H	1.670286	-5.372354	3.091473
H	1.747745	-4.928480	4.613150
O	2.265970	-3.688045	5.742559
H	1.950165	-3.939108	6.647925
H	3.250811	-3.630582	5.784058
O	1.320967	-1.238887	5.035724
H	1.669047	-2.120200	5.321380
H	1.648765	-0.571389	5.688085
O	2.221925	0.631064	6.799566
H	1.895453	0.412861	7.709952
H	1.865023	1.526472	6.583187
O	1.295513	0.012285	9.268477
H	1.591791	-0.890562	9.555786
H	1.635049	0.658078	9.942550
O	2.189452	1.814714	11.064008
H	1.866979	2.713894	10.814457
H	3.167606	1.850970	11.053249
O	1.231211	4.287137	10.373730
H	0.252457	4.300246	10.329681
H	1.553967	4.564956	9.488601
O	2.228294	-7.473584	-7.138330
H	1.983538	-6.556604	-7.402015
H	3.207805	-7.507010	-7.135066
O	1.422644	-8.003516	-4.622643
H	0.435750	-8.018855	-4.616151
H	1.696600	-7.791373	-5.553805
O	2.362553	-6.178595	-2.855386
H	2.038262	-6.451395	-1.962865
H	2.020296	-6.850815	-3.498856
O	1.450537	-3.716664	-3.564369
H	1.788630	-4.613713	-3.320491
H	1.795822	-3.508629	-4.468396
O	2.221415	-1.842886	-1.750450
H	1.919367	-2.531771	-2.392716
H	1.880842	-2.102082	-0.858679

O	1.323823	-2.498222	0.737292
H	0.334532	-2.458947	0.735971
H	1.632133	-1.828858	1.396820
O	2.195088	-0.635555	2.532786
H	1.879181	-0.854732	3.444389
H	3.183225	-0.649171	2.574099
O	1.331830	1.847603	1.809537
H	1.669250	0.948436	2.045834
H	1.688151	2.074212	0.915145
O	2.020629	3.749248	3.637219
H	1.735981	3.042336	3.007785
H	1.702539	3.495114	4.538362
O	1.242563	3.116425	6.171572
H	0.255897	3.137630	6.225627
H	1.582145	3.811306	6.786350
O	2.209241	5.038381	7.877411
H	1.886330	5.933655	7.630462
H	3.196309	5.086194	7.888426
O	1.225385	7.538867	7.173281
H	1.561461	8.222473	7.773257
H	1.577910	7.766513	6.268538
O	2.444883	-4.330408	-10.290179
H	2.155963	-3.429299	-10.573217
H	3.423699	-4.322094	-10.278812
O	1.472262	-4.917977	-7.841779
H	0.483900	-4.916270	-7.884586
H	1.805356	-4.697260	-8.751014
O	2.371423	-3.103038	-6.046864
H	2.027923	-3.752110	-6.713441
H	2.048716	-2.212660	-6.329631
O	1.461479	-0.633432	-6.825849
H	0.474935	-0.640486	-6.873479
H	1.700379	0.050906	-6.153372
O	2.193827	1.266667	-4.999302
H	1.897383	1.041189	-4.083665
H	3.183493	1.257892	-4.973832
O	1.385147	0.652903	-2.466030
H	1.701178	-0.239310	-2.181592
H	1.723145	1.309843	-1.809021
O	2.239146	2.527977	-0.671099
H	1.889549	3.416244	-0.931409
H	3.225215	2.602932	-0.683462
O	1.305835	4.979573	-1.401814
H	0.316114	4.990786	-1.401166
H	1.602225	5.654142	-0.741489
O	2.213907	6.809898	0.395954
H	1.922201	7.716778	0.127289
H	3.201676	6.804511	0.350719
O	1.280851	6.278402	2.914800
H	1.553732	5.359470	3.154620
H	1.640376	6.456311	2.011202
O	2.180868	8.104565	4.736748
H	1.892413	7.428131	4.075065
H	3.168002	8.091880	4.747293
O	1.245589	10.605957	3.880244
H	0.258355	10.576171	3.881198
H	1.551454	9.725003	4.183947
O	1.569866	-1.854401	-11.060565

H	0.590910	-1.815897	-11.054973
H	1.878009	-1.161407	-10.434043
O	2.508704	0.052447	-9.282787
H	2.145591	-0.173822	-8.393760
H	2.143060	0.944957	-9.515698
O	1.540967	2.480227	-9.973545
H	0.561370	2.513300	-9.978637
H	1.841776	3.175039	-9.346733
O	2.462016	4.406242	-8.198112
H	2.148583	4.175808	-7.289077
H	3.450401	4.383655	-8.185796
O	1.438174	3.783106	-5.740184
H	1.720237	2.881068	-5.448036
H	1.745191	4.415556	-5.046016
O	2.265812	5.609513	-3.866272
H	1.893135	5.406999	-2.972187
H	1.914809	6.493418	-4.129329
O	1.328590	8.064902	-4.664493
H	0.339665	8.030428	-4.732705
H	1.667717	8.281239	-5.562661
O	2.023102	9.974336	-2.901503
H	1.617362	10.809438	-3.178612
H	1.743685	9.292462	-3.567875
O	1.313123	9.270848	-0.318732
H	1.585242	9.533102	-1.226986
H	1.653874	9.972509	0.301074
O	2.194203	11.142189	1.382103
H	1.869548	10.948170	2.296693
H	3.172161	11.151115	1.425261
O	6.195708	6.859280	-8.695122
H	5.771110	7.534279	-8.127275
H	7.159186	6.901282	-8.482254
O	5.069010	8.820064	-7.017946
H	4.083714	8.821918	-7.075047
H	5.356864	9.700100	-7.304356
O	6.434344	-9.597890	2.836246
H	6.025045	-9.534381	1.944266
H	7.397390	-9.467194	2.721332
O	5.139242	-8.068968	4.666553
H	5.621544	-8.639689	4.014002
H	5.520465	-8.261342	5.551422
O	6.208843	-8.511683	7.208070
H	6.052720	-9.422657	7.501891
H	7.190847	-8.382225	7.222769
O	5.128588	-6.703088	8.957405
H	5.446627	-6.926576	9.845538
H	5.515807	-7.380323	8.351662
O	5.914813	-4.142202	8.257680
H	5.672348	-5.069851	8.480705
H	5.558216	-3.961717	7.351552
O	4.888498	-2.438592	10.064323
H	5.257721	-3.101056	9.421629
H	5.269492	-2.655901	10.928711
O	5.181328	-10.426164	-3.918678
H	4.190938	-10.403584	-3.931728
H	5.494005	-9.524718	-4.152165
O	6.135202	-11.077518	-1.564287
H	5.859391	-11.985959	-1.367479

H	5.733055	-10.847202	-2.458664
O	5.150525	-9.351496	0.382227
H	5.501319	-9.957290	-0.309548
H	5.356574	-8.432557	0.082006
O	5.734328	-6.790588	-0.310971
H	5.389599	-6.118001	0.327976
H	5.467980	-6.499367	-1.218605
O	4.843768	-4.949025	1.469122
H	5.171146	-5.139030	2.383308
H	5.176719	-4.050336	1.222639
O	5.737440	-5.499740	3.977933
H	5.486931	-6.417604	4.247055
H	6.726577	-5.490545	3.944278
O	5.005820	-3.580126	5.759511
H	5.284903	-4.274504	5.112171
H	5.404873	-2.729514	5.457493
O	6.049571	-1.154369	4.978491
H	5.711531	-0.482761	5.620156
H	7.026905	-1.045098	4.913030
O	4.957546	0.626787	6.751246
H	3.968364	0.612177	6.749912
H	5.214032	1.556522	6.528150
O	5.873690	-0.012743	9.231471
H	5.460845	-0.866849	9.500571
H	5.512239	0.211381	8.336706
O	5.011354	1.796498	11.040885
H	5.361275	1.514973	11.900000
H	5.345635	1.136239	10.376998
O	5.907792	4.282204	10.380418
H	5.662539	4.903873	11.082495
H	5.546720	3.402248	10.651676
O	5.018778	-7.570894	-7.061969
H	5.389648	-8.269545	-7.621891
H	5.380532	-7.722505	-6.147381
O	6.059637	-7.874641	-4.609657
H	5.705924	-7.203070	-3.975502
H	7.027557	-7.695779	-4.694580
O	5.082550	-6.050556	-2.835566
H	4.094817	-6.029290	-2.887577
H	5.421591	-5.145004	-3.040010
O	6.140109	-3.582609	-3.421136
H	5.686993	-2.933002	-2.828050
H	5.800325	-3.399875	-4.330027
O	4.930004	-1.816714	-1.740212
H	3.940274	-1.834366	-1.760570
H	5.200120	-0.895677	-1.982568
O	5.742740	-2.484186	0.756625
H	5.441957	-2.235017	-0.153502
H	6.730785	-2.499978	0.725237
O	4.912078	-0.624819	2.547611
H	5.324626	-0.833442	3.421012
H	5.235646	-1.304894	1.905215
O	5.641717	1.897652	1.863920
H	5.333489	0.978618	2.062072
H	5.375560	2.111584	0.935087
O	4.728827	3.748990	3.636135
H	3.738616	3.731718	3.637750
H	5.024038	3.064164	2.986795

O	5.678252	3.169607	6.119198
H	5.345108	3.384698	5.212696
H	6.666296	3.189776	6.074143
O	4.955117	5.091045	7.897705
H	5.278175	4.803178	8.781033
H	5.216425	4.377890	7.263286
O	6.207057	7.399287	6.992861
H	5.837432	6.560427	7.349310
H	7.175628	7.285784	6.911440
O	5.275318	-4.399771	-10.223889
H	5.594860	-5.034090	-10.883598
H	5.643694	-3.520521	-10.490793
O	6.036451	-5.091705	-7.654658
H	5.615257	-5.968742	-7.495192
H	5.817615	-4.845401	-8.582693
O	5.102343	-3.156056	-5.940846
H	4.113802	-3.179761	-5.966147
H	5.416448	-3.853855	-6.564192
O	5.830328	-0.630542	-6.660884
H	5.530984	-1.549274	-6.451788
H	5.592054	-0.443536	-7.602892
O	4.902123	1.269469	-4.945719
H	5.251245	0.609551	-5.593589
H	5.218996	2.164323	-5.222503
O	5.725688	0.682045	-2.426332
H	5.441649	0.898308	-3.349106
H	6.716590	0.697701	-2.424465
O	4.958649	2.586281	-0.672540
H	5.245759	1.904074	-1.330958
H	5.384041	3.436772	-0.941927
O	6.116725	4.962932	-1.398632
H	5.747873	5.620124	-0.758369
H	5.728129	5.208227	-2.273714
O	4.950107	6.751730	0.317768
H	5.213589	6.574326	1.254638
H	5.266835	7.662381	0.099050
O	5.618570	6.203224	2.895496
H	5.271142	5.313706	3.156919
H	6.603859	6.132129	2.934570
O	4.938574	8.100845	4.709905
H	5.394832	7.838154	5.552141
H	5.183234	7.410248	4.043291
O	5.844621	10.493707	3.945062
H	5.543763	11.132626	4.609282
H	5.502950	9.601412	4.236961
O	6.303932	-1.965299	-10.856570
H	6.211855	-1.754401	-11.798299
H	7.286423	-2.029110	-10.677702
O	5.240030	0.024411	-9.227130
H	4.252688	0.045346	-9.295271
H	5.560586	-0.675316	-9.838913
O	6.526775	2.486449	-9.586996
H	6.125662	1.598793	-9.462247
H	7.492533	2.395971	-9.457577
O	5.197426	4.405730	-8.170910
H	5.578926	5.296814	-8.388749
H	5.687745	3.733704	-8.707039
O	5.738315	3.773346	-5.596973

H	5.511136	4.050558	-6.520525
H	5.424649	4.483349	-4.984010
O	4.993349	5.670939	-3.799609
H	4.004665	5.687868	-3.781495
H	5.290624	6.582120	-4.043305
O	5.886081	8.175757	-4.430758
H	5.663792	8.400290	-5.361242
H	5.438671	8.866346	-3.870390
O	4.811168	10.051890	-2.845380
H	3.830820	10.039476	-2.843121
H	5.100481	9.787064	-1.943324
O	5.811535	9.231522	-0.410159
H	5.634068	9.922943	0.266387
H	6.789450	9.178754	-0.545329
O	5.022886	11.146134	1.422799
H	5.314510	10.939391	2.343552
H	5.366733	12.030245	1.223327
O	8.874780	6.942893	-8.163606
H	9.183078	7.491142	-7.408943
H	9.219188	6.039526	-7.990888
O	10.073188	8.301637	-6.037477
H	10.720358	7.583341	-5.892883
H	9.586320	8.406491	-5.195321
O	9.286651	-9.392563	2.645131
H	9.606643	-10.276423	2.882808
H	9.591106	-9.244019	1.718953
O	9.860657	-7.555317	4.688402
H	9.732367	-8.226785	3.982453
H	9.492202	-7.920921	5.522847
O	8.901368	-8.101896	7.235379
H	9.366726	-8.820778	7.690307
H	9.214972	-7.256266	7.665490
O	9.993208	-5.859712	8.184803
H	10.582444	-5.674719	7.416383
H	9.470150	-5.040000	8.317619
O	8.584104	-3.395571	8.183189
H	7.641321	-3.676409	8.226255
H	8.811255	-3.369688	7.221744
O	11.390484	-5.435860	5.830251
H	11.026394	-6.154802	5.277314
H	10.892673	-4.627652	5.588341
O	10.078859	-9.595958	-3.940443
H	10.547178	-10.234462	-4.498852
H	9.548800	-10.124376	-3.300365
O	8.832106	-10.910771	-1.835757
H	7.847748	-10.999491	-1.754096
H	9.192349	-11.803181	-1.717064
O	9.841622	-9.056565	-0.037868
H	9.439523	-9.773800	-0.578164
H	10.548823	-8.682322	-0.619309
O	8.413313	-6.746412	-0.281300
H	7.422723	-6.797396	-0.259264
H	8.775271	-7.652062	-0.136450
O	9.446443	-4.955068	1.407168
H	10.412723	-5.019575	1.370357
H	9.088718	-5.647321	0.787003
O	8.433704	-5.375599	3.927098
H	8.876303	-6.207545	4.226596

H	8.793956	-5.201479	3.026735
O	9.441721	-3.448495	5.583529
H	8.940151	-4.061844	4.997662
H	9.290049	-2.539491	5.228946
O	8.815121	-0.939130	4.745326
H	9.156803	-0.290689	5.411044
H	9.217155	-0.714612	3.878431
O	9.537881	0.748650	6.724264
H	9.140045	0.269589	7.490996
H	8.976637	1.542850	6.553455
O	8.537617	-0.647609	8.873194
H	7.579986	-0.445267	8.994095
H	8.598519	-1.614446	8.693322
O	9.885465	1.528851	10.146590
H	9.457693	0.716272	9.810819
H	10.515293	1.808955	9.445204
O	8.705702	4.032603	10.214527
H	7.729518	4.076133	10.278446
H	8.962301	3.083588	10.256204
O	11.517296	-2.238933	-7.749404
H	11.232258	-2.976995	-8.324567
H	11.038336	-2.422552	-6.915298
O	8.738837	-7.315981	-4.904111
H	9.206856	-8.171639	-4.802826
H	8.994241	-6.813654	-4.088905
O	9.550212	-6.016973	-2.652116
H	9.026311	-6.236848	-1.846106
H	9.401415	-5.056295	-2.821707
O	8.907419	-3.444545	-3.242467
H	7.920390	-3.415232	-3.254818
H	9.227371	-2.764068	-2.610110
O	9.658948	-1.484409	-1.463259
H	10.454606	-1.084237	-1.055048
H	9.143658	-0.704455	-1.804053
O	8.451480	-2.497507	0.732199
H	8.839264	-3.379054	0.941336
H	8.830912	-2.213126	-0.135866
O	9.622260	-0.507677	2.147391
H	9.165538	-1.257652	1.691320
H	10.386004	-0.283358	1.573616
O	8.332107	1.872985	1.936456
H	7.340410	1.854852	1.925557
H	8.658688	0.951465	2.074564
O	9.383163	3.661215	3.610605
H	10.348665	3.618058	3.539357
H	9.020542	2.973433	2.988314
O	8.383703	3.127895	6.111399
H	8.765854	3.322918	5.224344
H	8.809137	3.767163	6.738051
O	9.706403	4.752188	7.814501
H	9.292992	4.629373	8.707763
H	9.555287	5.677587	7.526186
O	9.066990	7.310321	6.812404
H	9.370441	8.021978	7.396718
H	9.388189	7.553498	5.913991
O	10.135059	-4.339972	-9.073004
H	10.555325	-4.975917	-9.670900
H	9.671041	-4.867991	-8.382147

O	8.688521	-5.392905	-6.989707
H	7.733454	-5.317224	-7.227067
H	8.771231	-6.130635	-6.344893
O	9.619708	-3.046981	-5.842238
H	9.462464	-3.158294	-4.874775
H	9.246385	-3.860034	-6.254330
O	8.521411	-0.626467	-6.434065
H	7.535000	-0.639460	-6.510860
H	8.812366	-1.545089	-6.216310
O	9.484042	1.285260	-4.692551
H	9.137981	0.617425	-5.326805
H	9.101744	2.151119	-4.959683
O	8.414531	0.708931	-2.327049
H	8.827416	0.943193	-3.205741
H	8.769134	1.349439	-1.661828
O	9.552823	2.295781	-0.446724
H	9.037028	2.225137	0.392950
H	9.413889	3.216100	-0.776583
O	8.877300	4.775651	-1.332085
H	7.892812	4.832084	-1.396857
H	9.227147	5.050077	-2.211151
O	9.397071	6.719728	0.494193
H	8.896190	6.456245	1.302450
H	9.312482	5.970340	-0.143784
O	8.318771	6.103169	2.923709
H	8.721777	5.247678	3.200479
H	8.729528	6.807204	3.479047
O	9.681268	8.094652	4.215219
H	10.436817	8.052617	3.589673
H	9.249896	8.962193	4.033763
O	8.554669	10.519196	3.525595
H	7.581429	10.532240	3.693340
H	8.924626	11.252724	4.040337
O	8.900788	-2.149975	-10.362298
H	9.219506	-2.967575	-9.926499
H	9.228877	-1.404263	-9.809622
O	9.917013	-0.184198	-8.693840
H	10.697483	-0.727573	-8.437710
H	9.321742	-0.276684	-7.906347
O	9.396718	2.403559	-9.396300
H	9.707299	2.588693	-10.295537
H	9.718733	1.495047	-9.178829
O	9.880676	4.425830	-7.513479
H	9.848076	3.737301	-8.212706
H	9.274688	4.112309	-6.796866
O	8.421076	3.741902	-5.349709
H	7.435217	3.760589	-5.440202
H	8.674699	4.466332	-4.733068
O	9.548178	5.699973	-3.803245
H	10.372332	5.745349	-4.333472
H	9.166711	6.612171	-3.772115
O	8.529655	8.226131	-3.659066
H	8.562748	8.555802	-2.733220
H	7.581533	8.229907	-3.935346
O	11.471595	5.853834	-5.804164
H	12.425737	5.694413	-5.754381
H	11.114746	5.262671	-6.507697
O	8.486642	8.904421	-0.902078

H	8.719555	8.065718	-0.435625
H	8.973219	9.578672	-0.371211
O	9.872769	10.335054	1.009779
H	10.504419	9.646864	1.302950
H	9.300009	10.486651	1.788068
O	11.415390	-7.995349	-2.030669
H	11.132348	-8.593042	-2.753288
H	10.966704	-7.149936	-2.240057
O	11.277154	8.053603	1.921250
H	10.802369	7.437054	1.314187
H	12.225471	7.885374	1.818446
O	11.375510	2.463574	7.975125
H	10.900484	1.845182	7.381292
H	10.965170	3.338727	7.825129
O	-11.282049	-2.486829	-7.976071
H	-10.791948	-2.646969	-7.143110
H	-10.977105	-3.212865	-8.558604
O	-11.436180	5.909018	-6.006704
H	-12.397612	6.004929	-5.937283
H	-11.067616	6.812185	-6.140649
O	-11.467890	-5.825812	5.987932
H	-11.190155	-5.922287	6.926442
H	-12.429643	-5.708620	5.991455
O	11.296111	0.239392	0.030597
H	12.253618	0.386674	0.030913
H	10.873962	1.096649	-0.209973
O	-11.272277	0.021410	-0.089942
H	-10.904647	-0.197518	0.790333
H	-10.879020	0.885611	-0.327867
